# Visible Light-Harvesting Dinuclear Chromium(III) Complex with Anthracene-Based Phosphorescence for Photon Upconversion and Photocatalysis

**DOI:** 10.1021/jacs.6c13239

**Published:** 2026-10-02

**Authors:** Dennis Fritzler, Katharina L. Deuter, Nikita Vashistha, Serhiy Demeshko, Michael Linseis, Pengyue Jin, Franc Meyer, Benjamin Dietzek-Ivanšić, Rainer F. Winter, Cui Wang

**Affiliations:** † Department of Chemistry, University of Konstanz, Universitätsstraße 10, 78464 Konstanz, Germany; ‡ Leibniz Institute of Photonic Technology, e. V. Jena, Albert-Einstein-Straße 9, 07745 Jena, Germany; § Institute of Inorganic Chemistry, University of Göttingen, Tammannstraße 4, 37077 Göttingen, Germany; ∥ Leibniz Institute of Surface Engineering (IOM), Permoserstraße 15, 04318 Leipzig, Germany

## Abstract

Luminescent 3d^3^ chromium­(III) complexes are emerging as highly attractive, earth-abundant alternatives to precious 4d or 5d metal complexes in photophysical and photochemical applications. However, the Laporte-forbidden nature of their d–d transitions in the visible region severely hampers the light-harvesting efficiency of conventional photoactive Cr^III^ complexes. To overcome this fundamental bottleneck, we anchored two anthracene-based antennas to the polypyridine ligands in a dinuclear Cr^III^ complex, yielding the complex Cr_2_-An. The resulting energetically low-lying π–π* transition in the anthracene antenna enhances visible light absorption at 450 nm by a factor of 30 relative to the anthracene-free reference complex, Cr_2_-ref Both dimers exhibit ferromagnetic ground-state exchange interactions and display room temperature luminescence, albeit originating from different excited states. Specifically, Cr_2_-An exhibits anthracene-based phosphorescence with a lifetime of ∼150 ns in solution at room temperature, which is likely accessible via two pathways: direct intersystem crossing from the singlet excited state of the anthracene fragment, or intramolecular doublet-triplet energy transfer from the spin-flip excited states of the Cr^III^ centers. These exceptional photophysical properties render Cr_2_-An an attractive photosensitizer for triplet–triplet annihilation upconversion (TTA-UC) and singlet oxygen-mediated oxidation reactions, delivering photocatalytic performance that surpasses the benchmark [Ru­(bpy)_3_]­Cl_2_ under visible-light irradiation. These findings offer a powerful molecular design strategy for bypassing the intrinsic optical limitations of first-row transition metals, opening new avenues for sustainable and efficient light energy conversion.

## Graphical abstract

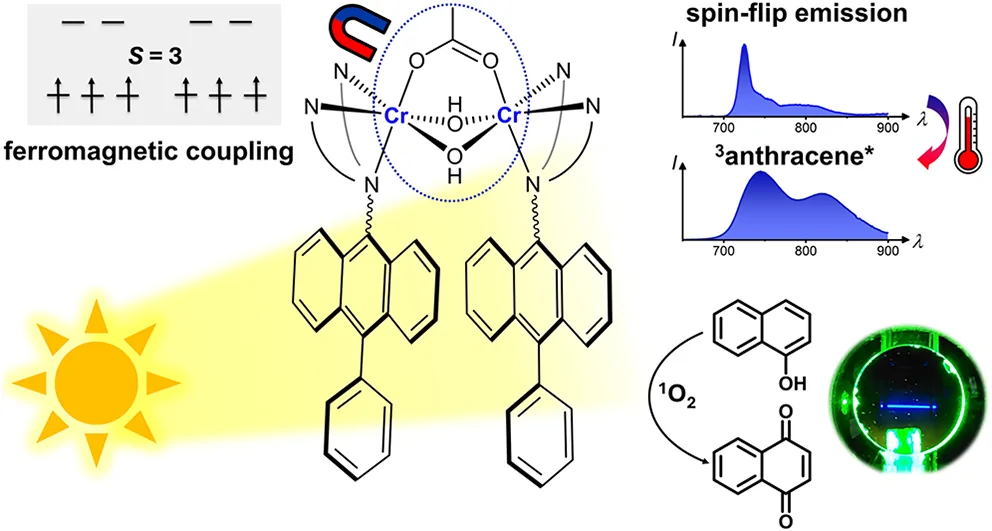

## Introduction

Photoactive coordination complexes are pivotal to the fields of photophysics and photochemistry.^1^ The last decade has witnessed significant breakthroughs in the design of photoactive complexes based on earth-abundant first-row transition metals and unearthed key design principles for making photoactive 3d metal complexes competitive to those based on 4d/5d noble metals.^2–6^ These coordination compounds are emerging luminophores and sensitizers in the fields of bioimaging and sensing,^7–11^ lighting,^12–16^ photon upconversion,^17–29^ and photocatalysis.^30–48^

Recent progress in luminescent 3d^3^ Cr^III^ coordination compounds has focused on mononuclear complexes bearing tridentate chelating polypyridyl ligands.^49–55^ These ligands show predominantly strong σ-donating/π-accepting character and form nearly perfect octahedral geometry with the metal center, with the N^Cr^III^^N bite angles approaching 90°. This leads synergistically to a large ligand-field splitting and enhanced molecular rigidity, giving strong photoluminescence from the spin-flip doublet excited states (^2^T_1_/^2^E) with long lifetimes of up to milliseconds,^49–56^ as shown with selective examples in Figure 1a. Such high photoluminescence performance, paired with reversible redox properties in some cases,^34,35,46^ renders mononuclear Cr^III^ complexes potent photosensitizers for applications based on electron- and energy-transfer processes.^17,34,35,44–46,57–60^

**Figure 1:**
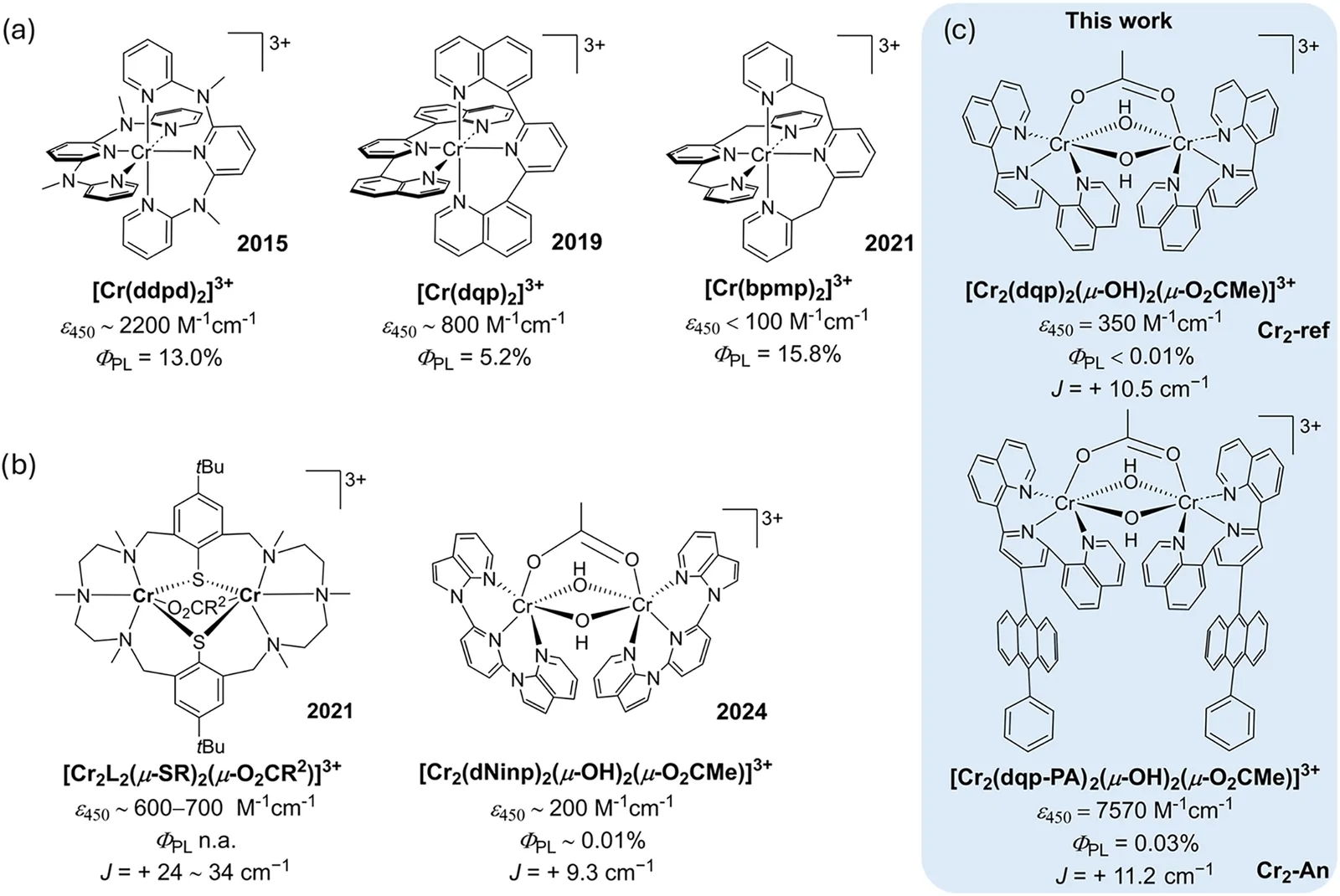
Molecular structures of (a) selected highly luminescent mononuclear Cr^III^ complexes, including [Cr­(ddpd)_2_]^3+^ (ddpd = *N*,*N*′-dimethyl-*N*,*N*′-dipyridin-2-ylpyridine-2,6-diamine),^54^ [Cr­(dqp)_2_]^3+^ (dqp = 2,6-di­(quinolin-8-yl)­pyridine),^49^ and [Cr­(bpmp)_2_]^3+^ (bpmp = 2,6-bis­(2-pyridylmethyl)­pyridine),^52^ (b) recently reported luminescent dinuclear Cr^III^ complexes [Cr_2_L_2_(μ-SR)_2_(μ-O_2_CR^2^)]^3+^ ^65^ and [Cr_2_(dNinp)_2_(μ–OH)_2_(μ-O_2_CMe)]^3+^ (dNinp = 2,6-di­(*N*-7-azaindol-1-yl)­pyridine)^66^ with ferromagnetic exchange interactions, and (c) dinuclear Cr^III^ complexes (dqp-PA = 8,8′-(4-(10-phenylanthracen-9-yl)­pyridine-2,6-diyl)­diquinoline) with triflate counter anions presented in this work, along with their molar extinction coefficients at 450 nm ε_450_ and photoluminescence quantum yields Φ_PL_ in solution at room temperature. The ferromagnetic coupling constants *J* are given for the dinuclear Cr^III^ complexes.

In contrast to the substantial luminescence and photochemical studies of mononuclear complexes, the research of dinuclear Cr^III^ counterparts has predominantly focused on the magnetic properties,^61^ aside from a few early studies.^62–64^ The magnetic exchange interaction between the two Cr^III^ centers determines the alignment of their electron spins, giving either ferromagnetic (*S* = 3) or antiferromagnetic coupling (*S* = 0). The sign and magnitude of the exchange interaction depend on the molecular orbital overlap between the two metal centers, which can be mediated by the precise geometry of the Cr_2_ core and the bridging units.^61,67,68^ Exchange coupling between the two metal centers modifies the electronic structure and can enhance the intensity of otherwise weak spin-forbidden transitions, providing an intrinsic optical advantage for exchange-coupled dinuclear complexes.^63,64,67^ In principle, dinuclear Cr^III^ complexes retain the metal-centered (MC) transitions of their mononuclear analogues, formally involving only one of the two Cr^III^ centers.^67,69^ However, examples of luminescent dinuclear Cr^III^ complexes remain very scarce,^70^ because the ligand character and the coordination geometry in these dimers are not as optimal as those found in their luminescent mononuclear counterparts. We recently established a molecular design concept for constructing room temperature luminescent dinuclear Cr^III^ complexes with ferromagnetic coupling in the electronic ground state.^70^ In this family, the combination of two hydroxo and one acetato bridging groups with tridentate chelating capping ligands has proven to be an effective structural motif for obtaining ferromagnetically coupled Cr^III^ dimers, as exemplarily shown for [Cr_2_(dNinp)_2_(μ–OH)_2_(μ-O_2_CMe)]^3+^ (dNinp = 2,6-di­(*N*-7-azaindol-1-yl)­pyridine)^66^ in Figure 1b.

Despite the progress achieved for the mono- or dinuclear luminescent Cr^III^ complexes mentioned above, fundamental challenges in using these complexes for efficient photochemical energy conversion still remain. An important bottleneck is their weak absorption in the visible range. The low-energy absorption band arises typically from the d–d transition, which is Laporte-forbidden. While increasing the ligand-field splitting enhances the photoluminescence for Cr^III^ complexes, as mentioned above, this inevitably shifts the ground state absorption toward the high-energy blue and UV regions. This has been exemplified by the highly luminescent molecular rubies, whose MC transitions exhibit modest extinction coefficients mostly below 1000 M^–1^ cm^–1^.^49–56^ This is also true for luminescent dinuclear Cr^III^ complexes,^66,70^ as shown in Figure 1 for selected complexes with their extinction coefficients at 450 nm. A few reports show that the visible absorption for luminescent Cr^III^ complexes can be enhanced by introducing intraligand charge transfer (ILCT).^71–73^ A recent study demonstrated that extending π-conjugation induces a bathochromic shift of the intraligand transitions, thereby significantly enhancing visible light absorption in mononuclear Cr^III^ complexes.^74^ Another approach to overcome this limitation is to attach strongly visible light-absorbing chromophores to the complexes, known as the “antenna effect”, which has been frequently employed for lanthanide systems.^75,76^ For mononuclear luminescent Cr^III^ complexes, pendant chromophores based on naphthalene or anthracene have been used to enhance their light-harvesting,^77–79^ but these antennas are mostly limited to high energy UV-A absorption. Using the “antenna effect” to enhance the visible absorption for luminescent dinuclear Cr^III^ complexes could deliver attractive coordination compounds for efficient visible light-harvesting and energy-conversion technologies, but this has remained largely underexplored so far.

In this work, we present two novel ferromagnetically coupled dinuclear Cr^III^ complexes bridged by two hydroxo and one acetato ligands, Cr_2_-ref and Cr_2_-An (Figure 1c). Both dinuclear complexes are coordinated to two tridentate dqp ligands (dqp = 2,6-di­(quinolin-8-yl)­pyridine), while Cr_2_-An contains two more phenyl anthracenes (PAs) on the backbone of the chelating ligands. These anthracene moieties drastically enhance the visible light absorption of Cr_2_-An, presumably due to the π → π* transitions in the dimeric-analogue (PA)_2_ fragment formed upon their π–π stacking interactions, as supported by theoretical calculations. While Cr_2_-ref exhibits room temperature luminescence from the Cr^III^-centered spin-flip excited states, the broader near-infrared (NIR) phosphorescence for Cr_2_-An arises from the triplet excited state of PA, supported by nanosecond- and femtosecond transient absorption studies. These unique photophysical properties make Cr_2_-An a promising photosensitizer for photon upconversion and ^1^O_2_-mediated photocatalysis. Notably, Cr_2_–An exhibits high photostability and superior photocatalytic activity relative to Ru^II^-based benchmark, offering a robust and sustainable alternative for efficient light energy conversion.

## Results and Discussion

### Synthesis and Characterization

Both the dqp ligand and the phenyl anthracene-decorated derivative (dqp-PA) are synthesized by adapting reported procedures (SI, Section 2).^80,81^ Cr_2_-ref was prepared according to a literature report^66^ via stepwise coordination with the corresponding tridentate ligands, in which the chromium­(III) chloride tetrahydrofuran complex was used as the metal precursor (Figure 2a). For the preparation of Cr_2_-An, sodium acetate (NaOAc) and water were added to provide the bridging units between the two Cr^III^ centers (Figure 2b), which leads to an improved yield for complexation (SI, Section 2.5). Mass spectrometry and elemental analysis confirmed the structures and the purities of the obtained Cr^III^ dimers (SI, Sections 2.4 and 2.5). Slow diffusion of diethyl ether into the complex solutions gave orange and dark red single crystals for Cr_2_-ref and Cr_2_-An, respectively, which were suitable for single crystal X-ray diffraction (XRD) analysis. Both dinuclear Cr^III^ complexes show two reversible reductions in their ground states (SI, Section 5). Their utility for photoredox catalysis is currently under exploration, but this is beyond the scope of current study.

**Figure 2:**
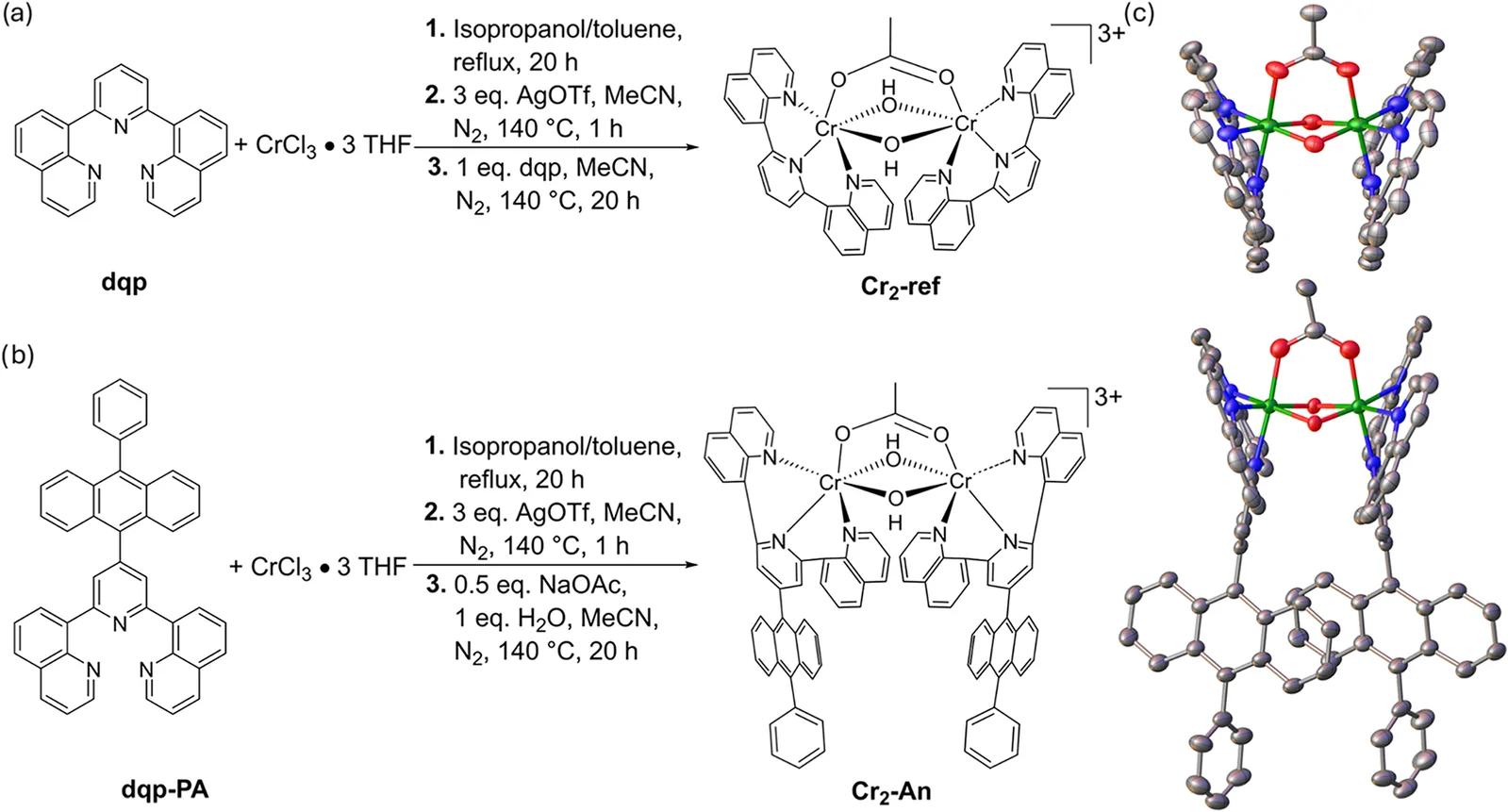
Synthetic procedures for dinuclear (a) Cr_2_-ref and (b) Cr_2_-An, together with (c) the X-ray single crystal structures for Cr_2_-ref (top) and Cr_2_-An (bottom). Hydrogen atoms, counter ions, and co-crystallized solvent molecules are omitted for clarity. Colour code: Cr = green; N = blue; O = red; C = gray. Thermal ellipsoids are drawn at 50 % probability level.

### Single-Crystal XRD Analysis

Single-crystal XRD analysis reveals a symmetrical butterfly-like structural motif for both dinuclear Cr^III^ complexes, in which the two Cr^III^ centers are bridged by one acetate and two hydroxo ligands (Figure 2c). The Cr–Cr distance of ∼2.95 Å for both dimers exceeds the typical value for a covalent chromium–chromium single bond.^68^ In these dimers, each Cr^III^ ion is coordinated to one tridentate dqp or dqp-PA ligand that spreads outwards, with N_L_–Cr–N_L_ bite angles of ∼99–102° for the lateral pyridines, implying a facial coordination mode (SI, Section 3, Table S1). The N_L_–Cr–N_C_ and N_L_–Cr–O_H/C_ angles are found close to 90° in both complexes, approaching the ideal parameters for an octahedral geometry, whereas the Cr–N bonds (2.03–2.09 Å) are slightly longer than the Cr^III^–O bonds (1.94–2.00 Å). These structural features provide a pseudo-octahedral coordination geometry for each Cr^III^ center, similar to that found in [Cr_2_(dNinp)_2_(μ–OH)_2_(μ-O_2_CMe)]^3+^ (Figure 1b).^66^ Notably, weak face-to-face π–π interactions seem viable between the two central pyridines in Cr_2_-ref, as evidenced by the interplanar distance of ∼3.69–3.81 Å that falls in the typical distance range for such interactions (SI, Section 3, Figure S1).^82^ Cr_2_-An shares a similar core structure to Cr_2_-ref, but with one additional phenyl anthracene moiety on the backbone of the central pyridine. Both anthracene planes are found nearly perpendicular to their central pyridines and orientate in an offset parallel manner with an interplanar distance of ∼3.31–3.91 Å, pointing to π–π interactions (Figure 2c, bottom, SI, Section 3, Figure S2).^82^

### Magnetic Properties

Dinuclear Cr^III^ complexes show predominantly antiferromagnetic exchange interactions (*S* = 0) in their electronic ground states,^61,83,84^ whereas ferromagnetically coupled systems (*S* = 3) have long been limited to only a few examples.^65,66,85–88^ The latter could be attractive magnetic molecules due to the sizeable magnetic moments in their ground states (*S* = 3).^89^ Temperature-dependent magnetic susceptibility measurements for both Cr^III^ dimers provide a χ_M_ *T* value of ∼4.3–4.5 cm^3^ mol^–1^ K at 295 K (Figure 3), which is clearly above the expected value of 3.75 cm^3^ mol^–1^ K for two non-interacting ions with *S* = 3/2 and *g* = 2.00. Decreasing the temperature leads to steadily rising χ_M_ *T* values, which achieves 6.4 cm^3^ mol^–1^ K for Cr_2_-ref and 5.9 cm^3^ mol^–1^ K for Cr_2_-An at 12 K and then drops rapidly to 5.2–5.9 cm^3^ mol^–1^ K at 2 K, likely due to the saturation effect.^90^ Fitting to the spin Hamiltonian *Ĥ* = −2*JŜ*_1_ *Ŝ*_2_ + *g*μ_B_ *B⃗*(*S⃗*_1_ + *S⃗*_2_) gives similar coupling constants with *J* of +10.5 cm^–1^ for Cr_2_-ref and +11.2 cm^–1^ for Cr_2_-An (SI, Section 4). These *J* values are higher than those of previously reported ferromagnetically coupled dinuclear Cr^III^ complexes with identical bridging units,^61,66,88^ but are lower than those for [Cr_2_L_2_(μ-SR)_2_(μ-O_2_CR^2^)]^3+^ complexes bridged by di-thiophenolate and acetate ligands (*J* of +24.2–34.8 cm^–1^) (Figure 1b).^65^ This is presumably attributed to the differences in the geometry of the bridging ligands within the Cr_2_ motif, for example variations in the Cr–O/S–Cr bond angles and the relative orientation of the donor atoms (SI, Table S1, Section 4.2).^70^

**Figure 3:**
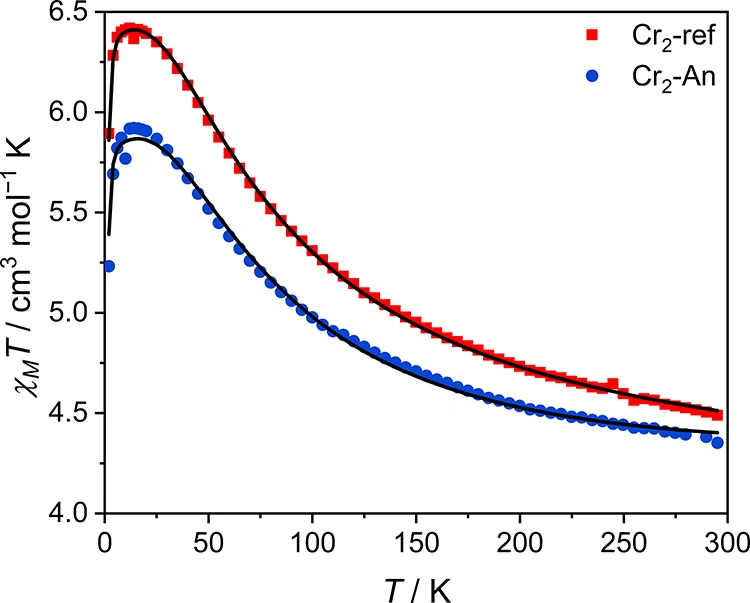
Variable-temperature magnetic susceptibility data for Cr_2_-ref (red squares) and Cr_2_-An (blue spheres). The black solid curves represent the best fit with the parameters *J* = +10.5 cm^–1^ and *g*_1_ = *g*_2_ = 2.07 for Cr_2_-ref and *J* = +11.2 cm^–1^ and *g*_1_ = *g*_2_ = 1.98 for Cr_2_-An.

For hydroxo-bridged Cr^III^ dimers, the Cr–O­(H)–Cr angles represent an important magnetostructural parameter.^91,92^ In Cr_2_-ref and Cr_2_-An, these angles are very similar, amounting to 98.7–99.1° and 98.2°, respectively, which are close to those found in the previously reported [Cr_2_(dNinp)_2_(μ–OH)_2_(μ-O_2_CMe)]^3+^ complex (97.8–97.9°) (SI, Table S1).^68^ This close structural similarity is consistent with the comparable ferromagnetic coupling constants observed for these systems. Notably, the pronounced modification of the peripheral ligand framework upon introduction of the anthracene-based antenna in Cr_2_-An leaves both the geometry of the magnetically relevant Cr_2_ core and the ferromagnetic exchange coupling essentially unchanged. While the precise magnitude of *J* remains sensitive to the geometry of the bridging unit,^67^ the [Cr_2_(μ–OH)_2_(μ-O_2_CMe)] core within the present class of complexes supported by tridentate chelating ligands appears to provide a robust structural platform for accessing ferromagnetically coupled dinuclear Cr^III^ complexes.^70^

### Ground-State Absorption and DFT Calculations

UV-Vis absorption spectrum of Cr_2_-ref shows a strong peak in the UV region (ε_363_ = 2.9 × 10^4^ M^–1^ cm^–1^) and a very weak band in the visible range (ε_450_ = 350 M^–1^ cm^–1^, Figure 4a, red traces, SI, Figure S5). The strong UV absorption is assigned to the ligand-to-metal charge transfer (LMCT) transition, whereas the weak visible absorption stems from the d-d transitions, as evidenced by the time-dependent density functional theory (TD-DFT) calculations (Figure 4b, SI, Section 7). These results agree with those reported for other mono- and dinuclear Cr^III^ complexes.^49–56,66,70^ Although spin-forbidden transitions could in principle be enhanced in exchange-coupled transition metal dimers,^66^ the modest visible absorptivity of Cr_2_-ref shows that this intrinsic advantage alone does not overcome the limited visible-light harvesting of the present Cr^III^ complex (Figure 4a). For Cr_2_-An, however, a distinct strong absorption band is observed in the range of 400–550 nm, with a high extinction coefficient ε_450_ = 7570 M^–1^ cm^–1^ that is 30 times stronger than the d–d transitions in Cr_2_-ref (Figure 4a, Table 1). Considering the Laporte-forbidden nature of the MC transitions and the very similar ferromagnetic exchange coupling in Cr_2_-ref and Cr_2_-An (*J* = +10.5 and +11.2 cm^–1^, respectively), the pronounced additional visible absorption of Cr_2_-An cannot be rationalized by a change in the exchange interaction and therefore points to transitions associated with the PA moieties. TD-DFT calculation for Cr_2_-An could not deliver meaningful results, primarily due to the structural complexity. We therefore performed TD-DFT calculations for the (PA)_2_ fragment extracted from the single-crystal structure in Cr_2_-An. This calculation reveals two energetically low-lying π–π* transitions within the dimeric (PA)_2_ fragment due to their π-orbital overlap. Importantly, the calculated absorption spectrum for (PA)_2_ closely resembles the experimental absorption difference spectrum between Cr_2_-An and Cr_2_-ref (Figure 4c). A linear combination of the calculated absorption spectra for Cr_2_-ref and (PA)_2_ represents the experimental absorption spectrum for Cr_2_-An very well (SI, Section 7, Figure S19). Based on the favorable agreement between the experimental results and the theoretical calculations, it seems plausible that the additional strong visible absorption for Cr_2_-An arises from the low-energy π–π* transitions in the dimeric (PA)_2_ fragment. This hypothesis is further supported by the femtosecond-transient absorption studies (SI, Section 8.1). Selective excitation of Cr_2_-An at 460 nm into the π–π* transition in (PA)_2_ with a femtosecond laser leads to a distinguishable transient absorption spectral feature for Cr_2_-An in the early domain, which is absent in Cr_2_-ref (see following section, SI, Figures S20 and S21). The (PA)_2_ fragment thus acts as a strongly visible light absorbing antenna for the dinuclear Cr^III^ complex. This addresses the weak visible absorption inherent to luminescent Cr^III^ complexes, overcoming a primary limitation for efficient solar energy harvesting.

**Figure 4:**
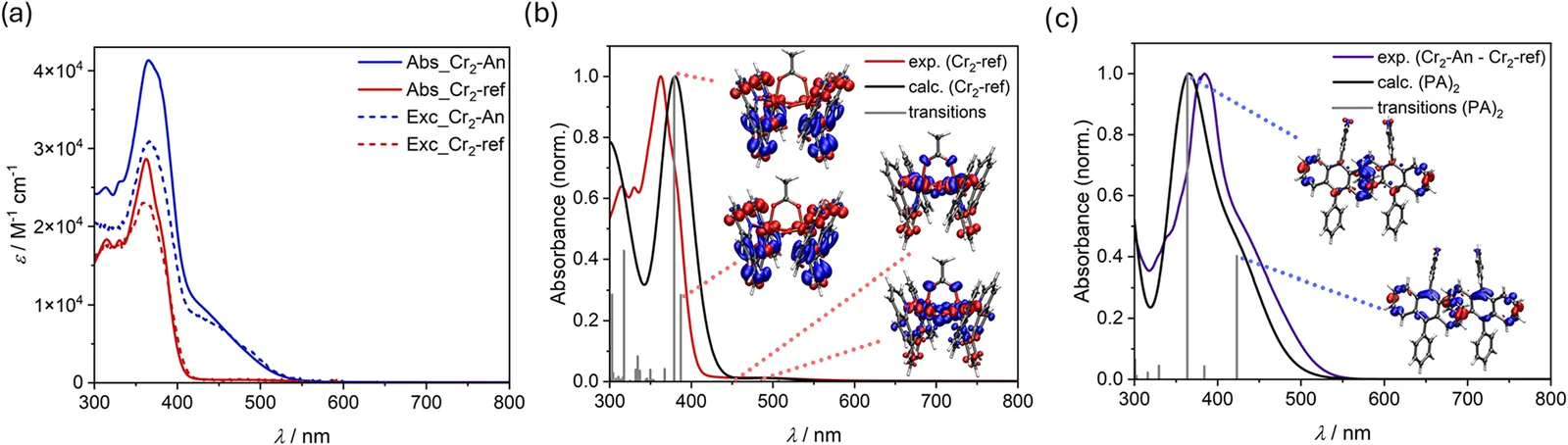
UV–Vis absorption of Cr_2_-ref (red) and Cr_2_-An (blue) measured in acetonitrile and their theoretical calculations. (a) UV–Vis absorption (solid traces) and excitation spectra (dotted traces) for Cr_2_-ref (red traces) and Cr_2_-An (blue traces) in acetonitrile at 20°C. The excitation spectra are detected at the 745 nm emission for Cr_2_-An (5 μM) and at 726 nm for Cr_2_-ref (15 μM). (b) UV–Vis absorption spectra for Cr_2_-ref obtained experimentally in acetonitrile (red traces) and from the TD-DFT calculations (black traces), along with the calculated transitions (gray lines) and their corresponding charge density difference plots for selected transitions (insets). (c) Experimental UV–Vis difference absorption spectrum obtained by subtracting Cr_2_-ref from Cr_2_-An using the data from Figure 3a (purple traces) and calculated UV–Vis absorption spectrum of the offset parallel oriented anthracene moieties (PA)_2_ (black traces), along with the calculated transitions for (PA)_2_ (gray lines) and their corresponding charge density difference plots for selected transitions (insets).

**Table 1:** Summary of the Key Photophysical Parameters for Cr_2_-ref and Cr_2_-An

	ε_363_ (M^–1^ cm^–1^)	ε_450_ (M^–1^ cm^–1^)	*E* ^a^ (eV)	λ_em_ (nm) (293 K)	τ_em_ ^b^ (ns) (293 K)	Φ_PL_ (%) (293 K)	λ_em_ ^c^(nm) (77 K)	τ_em_ ^c^ (μs) (77 K)
Cr_2_-ref	2.9 × 10^4^	350	1.71	726	283 ± 1 (272 ± 2)	<0.01	731/768	3.62 ± 0.02
Cr_2_-An	4.1 × 10^4^	7570	1.66 (1.71)	745/820	153 ± 1 (95 ± 1)	0.03	725/750/795	590 ± 3 ns; 3.90 ±0.02

a Excited state energy level estimated from the luminescence maximum at 726 nm for Cr_2_-ref. For Cr_2_-An, the energy levels are given for the ^3^PA* and the spin-flip excited states (in parentheses), and the former (1.66 eV) is estimated from the phosphorescence maximum at 745 nm.

b Luminescence lifetime values obtained in deaerated and aerated (values in parentheses) acetonitrile;

c Luminescence measurements in ethanol at 77 K.

### Excited-State Dynamics and Kinetics

Both Cr_2_-ref and Cr_2_-An fulfill the aforementioned structural requirements for constructing ferromagnetically coupled dinuclear Cr^III^ complexes with room temperature photoluminescence. For Cr_2_-ref at 293 K, light excitation at 375 nm leads to a dark red photoluminescence maximizing at 726 nm with a lifetime of ∼283 ns that is scarcely sensitive to oxygen (Figure 5a,b, Table 1), similar to the reported [Cr_2_(dNinp)_2_(μ–OH)_2_(μ-O_2_CMe)]^3+^.^66^ At 77 K, a more structured luminescence spectrum with two distinct bands at 731 and 768 nm from the spin-flip ^2^T_1_^4^A_2_/^2^E^4^A_2_ excited states is observed, which shows a luminescence lifetime of 3.6 μs (SI, Figure S8). Nanosecond (ns)-transient absorption measurements of Cr_2_-ref give an excited state absorption maximized at 407 nm and a ground state bleach signal that aligns with the absorption spectrum (Figure 5c, Figure 4a, SI, Section 8.2). The decay kinetics of the transient absorption signals mirror the time-resolved luminescence measurements and show negligible sensitivity to oxygen (SI, Section 8.2). A simplified energy level diagram for Cr_2_-ref is shown in Figure 6a.

**Figure 5:**
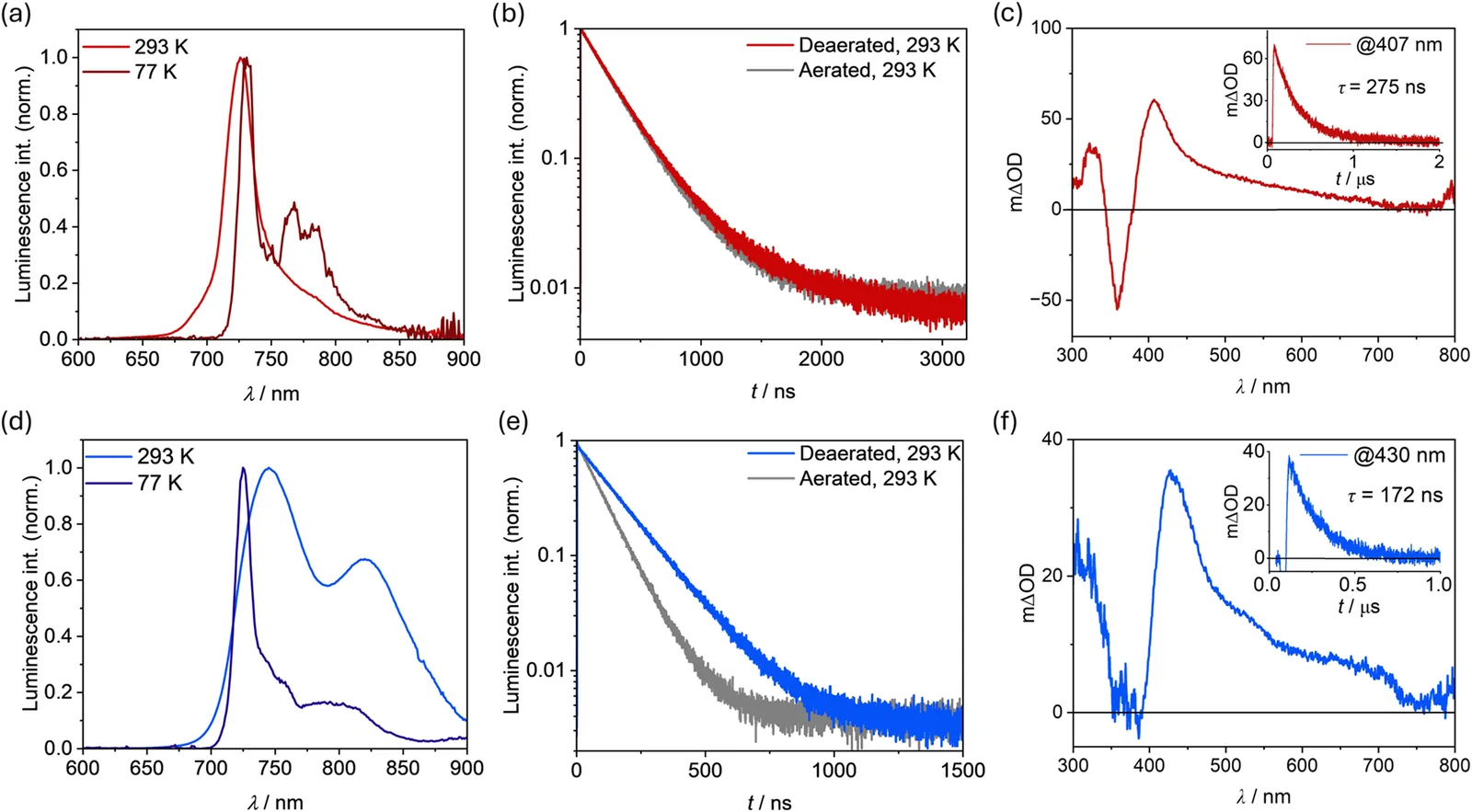
Luminescence measurements of Cr_2_-ref and Cr_2_-An. (a) Normalized luminescence spectra of Cr_2_-ref in acetonitrile at 293 K (red) and in ethanol at 77 K (dark red) with excitation at 375 nm. (b) Normalized luminescence decays of Cr_2_-ref recorded at 726 nm in aerated (gray traces) and deaerated (red traces) acetonitrile at 293 K following excitation at 375 nm. (c) Transient absorption spectrum for Cr_2_-ref (50 μM) in deaerated acetonitrile at 293 K under ns-pulsed laser excitation at 355 nm; inset: corresponding transient absorption decay kinetics recorded at 407 nm. (d) Normalized luminescence spectra of Cr_2_-An in acetonitrile at 293 K (blue) and in ethanol at 77 K (dark blue) with excitation at 450 nm. (e) Normalized luminescence decays of Cr_2_-An recorded at 745 nm in aerated (gray traces) and deaerated (blue traces) acetonitrile at 293 K, with excitation at 450 nm. (f) Transient absorption spectrum for Cr_2_-An (50 μM) in deaerated acetonitrile at 293 K under ns-pulsed laser excitation at 355 nm; inset: corresponding transient absorption decay kinetics recorded at 430 nm.

**Figure 6:**
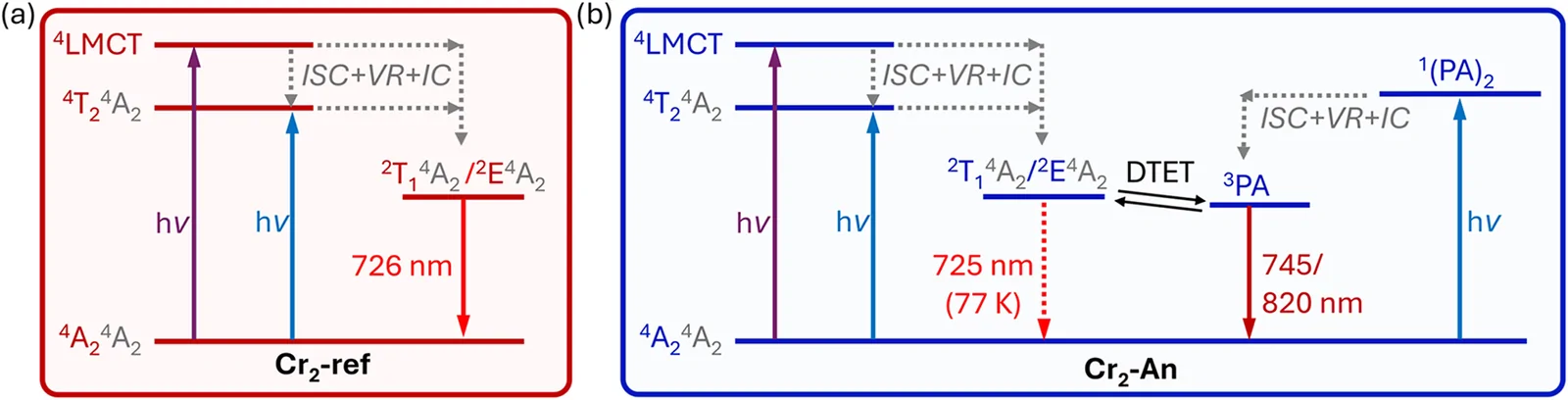
Simplified energy level diagrams for (a) Cr_2_-ref and (b) Cr_2_-An. ISC: intersystem crossing; VR: vibrational relaxation; IC: internal conversion; DTET: doublet-triplet energy transfer.

By comparison, selective excitation at 450 nm for Cr_2_-An in acetonitrile at 293 K leads to distinct broad NIR luminescence bands peaking at 745/820 nm, which closely resemble the phosphorescence spectra of triplet anthracenes at room temperature.^93,94^ The phosphorescence quantum yield for Cr_2_-An is determined to be 0.03% in acetonitrile at 293 K, which is more than 3-fold higher than that for Cr_2_-ref (Table 1, SI, Section 6). Time-resolved luminescence measurements give a time constant of 153 ns for Cr_2_-An in deaerated acetonitrile at room temperature, which is quenched to 95 ns in aerated solution (Figure 5e, Table 1). Transient absorption studies with ns-pulsed laser reveal the excited state absorption maximum at 430 nm for Cr_2_-An at room temperature (Figure 5f), corresponding to the excited state absorption of triplet PA (^3^PA*).^81,95,96^ The excitation spectrum for Cr_2_-An monitored at 745 nm essentially reproduces the absorption spectrum in the visible range (Figure 4a), suggesting a direct intersystem crossing (ISC) from the ^1^(PA)_2_* state to the triplet PA (^3^PA*) (Figure 6b). Transient absorption study with fs-pulsed laser for Cr_2_-An resolves this process with a time constant of 1.2 ps (SI, Section 8.1). This corresponds to an ISC rate constant of 8.3 × 10^11^ s^–1^, which exceeds that found for the phenyl-substituted anthracenes (∼10^6^–10^7^ s^–1^)^97^ by more than four orders of magnitude. Although chromium is not a traditional heavy atom, the presence of the metal ions nevertheless appears to enhance the rate of ISC. This is likely attributed to the paramagnetic nature of the Cr^III^ center, which enhances ISC via electron spin–spin exchange interactions with PA, a well-known phenomenon reported for organic radical-chromophore dyads.^98–100^ The unusual anthracene-based phosphorescence in solution at room temperature is likely facilitated by the rigid conformation of the two PAs confined by the aforementioned π–π interactions and the ligand framework, similar to those observed for anthracenes confined in rigid environments.^93,94^ After continuous irradiation with a 520 nm CW-laser (30 W cm^–2^) for 160 min, Cr_2_-An retains 98% of the initial phosphorescence intensity, highlighting the photorobustness of this dimer (SI, Figure S14).

Notably, the spin-flip excited states for Cr_2_-An become emissive at 77 K. The corresponding bi-exponential luminescence decay kinetics show an excellent agreement with the time constants for Cr_2_-ref on the one hand and for ^3^PA* on the other (Figure 5a,d, Table 1, SI, section 6, Figures S8 and S13). Our low-temperature measurements provide thus unequivocal evidence for the co-existence of the spin-flip and ^3^PA* excited states at 77 K, whereas the ^3^PA* becomes dominant at room temperature (Figure 5d, Figure 6b). The energy gap between the spin-flip excited states and ^3^PA is estimated to be 0.05 eV (Table 1). This makes an intramolecular doublet-triplet energy transfer (DTET) from the ^2^T_1_^4^A_2_/^2^E^4^A_2_ excited states of the Cr^III^ center to ^3^PA thermodynamically feasible, with a negligible activation barrier *E*_a_ estimated to be 0.28 kcal mol^–1^ (∼0.01 eV) (SI, Section 6.2) that allows reversible DTET.

The decay kinetics of Cr_2_-An obtained from the ns-transient absorption studies give time constants of 172 ns in deaerated and 105 ns in aerated acetonitrile at room temperature, which are close to the above-mentioned photoluminescence lifetimes (Table 1, SI, Section 8.2). To gain more insight into the excited state dynamics, we performed fs-transient absorption studies for both dimers. For Cr_2_-An, photoexcitation with a fs-laser at 370 nm gives the identical initial ^4^LMCT state as for Cr_2_-ref, which decays rapidly via ISC to a mixed “hot” state of the ^2^T_1_^4^A_2_/^2^E^4^A_2_ excited states and ^3^PA* with a time constant of 0.36 ps (SI, Section 8.1). Following an intramolecular DTET to ^3^PA*, a final non-decaying state is formed, in line with the ns-transient absorption measurements (Figure 6b, SI, Section 8.1, 8.2). No clear spectral features for the electron transfer products are observed, which suggests that the intramolecular DTET is a direct energy transfer process (SI, Section 8.2). The proposed excited state dynamics for Cr_2_-An are summarized in Figure 6b.

DTET has been observed for doublet photosensitizers, including organic radicals,^101–104^ luminescent Fe^III^ complexes with a ^2^LMCT excited state,^23,27–29,41,105–132^ and Cr^III^ complexes with spin-flip doublet excited states,^17,66,70,107^ but this still remains relatively underexplored compared to the traditional triplet–triplet energy transfer (TTET). For Cr_2_-An, the decay kinetics of ^3^PA* is substantially shorter than that expected for a decoupled PA chromophore. A recent report shows that in a PA-decorated Fe^III^ complex,^106^ the large energy gap of 0.35 eV makes intramolecular DTET an irreversible process, leading to a time constant of 11.5 μs for the decay of the non-emissive ^3^PA* state. The short lifetime observed for ^3^PA* in Cr_2_-An is presumably attributed to the reversible nature of DTET enabled by the minimal energy gap, similar to recent reports on other Fe^III^-anthracene or -perylene dyads involving a DTET reservoir, with a triplet lifetime ranging from 100 to 177 ns.^28,29,105^ These values are highly comparable to ours, despite lacking significant intramolecular π–π interactions between the organic chromophores. This suggests that such interactions do not appear to play a decisive role for the short lifetime of ^3^PA* in Cr_2_-An. Another potential depopulation mechanism for ^3^PA* might arise from the excited states exchange interactions between the Cr^III^ center and ^3^PA*, similar to those observed in molecular organic radical-chromophore dyads.^98–100^ Room temperature phosphorescence of anthracenes was recently observed in solid state-platinum­(II) complexes^94^ and gold­(I) complexes^108^ anchored with anthracene moieties. Our observation represents a very rare example of a room temperature phosphorescent anthracene in solution, which is accessible either by direct ISC from the ^1^(PA)_2_* state or via DTET from the spin-flip excited states of the Cr^III^ center (Figure 6b, SI, Section 8.1). Such room temperature phosphorescence for PA makes solution-based photophysical and photochemical applications viable, such as photon upconversion and photocatalysis.

### Photon Upconversion

Sensitized triplet–triplet annihilation upconversion (TTA-UC) allows the conversion of two low-energy photons to one of higher energy under non-coherent excitation.^109–112^ This biphotonic process is gaining increasing attention for photocatalytic reactions using low energy visible or NIR light.^113–121^ The strong visible light absorptivity, coupled with the ^3^PA excited state and the high photostability, makes Cr_2_-An a promising photosensitizer for TTA-UC. 9,10-Bis­((triisopropylsilyl)­ethynyl) anthracene (AnTIPS) is a well- known annihilator with a triplet state energy slightly lower than that for ^3^PA.^27,122^ Addition of AnTIPS to Cr_2_-An solution in deaerated 1,2-dichloroethane (DCE) clearly quenches the phosphorescence lifetime in a diffusion-controlled manner (SI, Section 9.1). For the upconversion pair Cr_2_-An (100 μM)/AnTIPS (20 mM) in deaerated DCE, selective excitation with a 532 nm CW-laser leads to upconversion luminescence maximized at 475 nm from the singlet excited state of AnTIPS (Figure 7a). An increase in the excitation power density drastically enhances the upconversion luminescence, which initially follows a quadratic dependence at low power densities and tends to approach a linear regime at high power densities (SI, Section 9.3), a characteristic for TTA-UC.^111,123,124^

**Figure 7:**
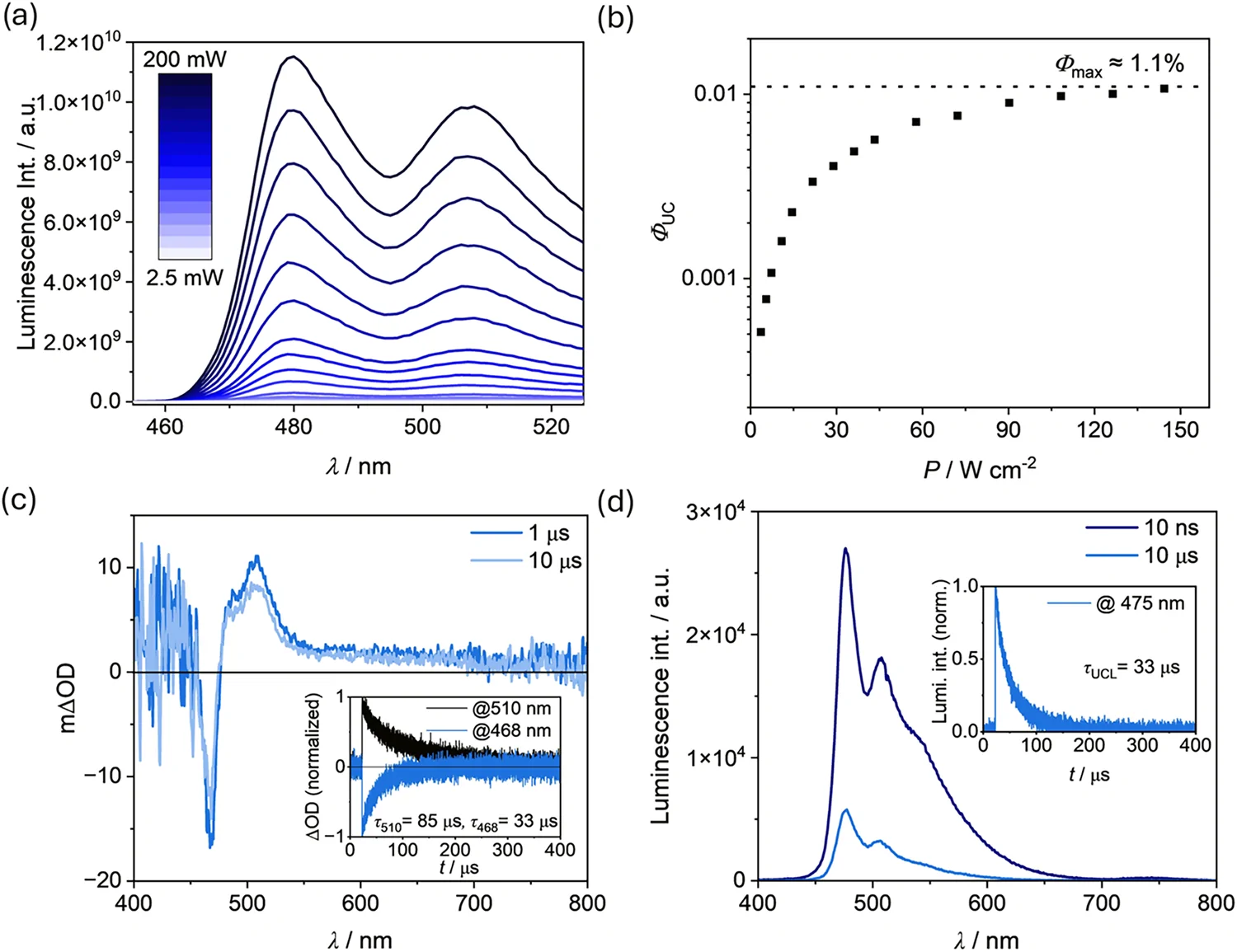
Photon upconversion studies of Cr_2_-An (100 μM)/AnTIPS (20 mM) in deaerated 1,2-dichloroethane (DCE) at room temperature. (a) Upconversion luminescence spectra of the Cr_2_-An/AnTIPS pair under 532 nm CW-laser excitation with increasing power; (b) upconversion quantum yields Φ_UC_ plotted as a function of the excitation power density of a 532 nm CW-laser; (c) transient absorption spectra of the upconversion pair recorded with different time delays after excitation with a ns-pulsed laser at 532 nm, along with the transient absorption decay kinetics recorded at 510 and 468 nm (inset); (d) time-gated luminescence spectra for the upconversion pair under 532 nm excitation with a ns-pulsed laser, along with the upconversion luminescence decay at 475 nm (inset). The decay kinetics in (c) and (d) are fitted mono-exponentially.

Nanosecond-transient absorption measurements for Cr_2_-An (100 μM)/AnTIPS (20 mM) in deaerated DCE under 532 nm excitation show the characteristic transient absorption signal at 510 nm for triplet AnTIPS, along with a stimulated emission at 468 nm arising from the singlet upconversion luminescence (Figure 7c). Notably, the corresponding decay kinetic agrees excellently with the observed time constant for the delayed fluorescence under identical conditions (Figure 7d), giving a time constant of 33 μs. This is roughly half of the triplet lifetime of AnTIPS under identical conditions (Figure 7c, inset), in line with the literature reports.^18,123,125^ Photoexcitation with a square-pulsed CW-laser (700 μs pulse width) leads to substantially slower decay kinetics with a time constant of 95 μs for the observed upconversion luminescence (SI, Section 9.3). This discrepancy is attributed to the laser pulse width and power, as demonstrated by us and others.^27,123^

Under our conditions, the maximal upconversion quantum yield Φ_UC_ for Cr_2_-An (100 μM)/AnTIPS (20 mM) is determined to be 1.1% (theoretical maximum is 50%),^126^ exhibiting a high photostability under continuous irradiation with a 532 nm CW-laser (144 W cm^–2^) (Figure 7b, SI, Section 9.3). This Φ_UC_ seems moderate but remains competitive to those obtained with doublet photosensitizers based on Fe^III^ complexes (Φ_UC_ from 0.002% to 1.3%)^23,27–29,105^ or a mononuclear Cr^III^ complex (Φ_UC_ up to 12%).^17^ Photon upconversion has been sensitized by dinuclear [Cr_2_(dNinp)_2_(μ–OH)_2_(μ-O_2_CMe)]^3+^ in our early report, but with a very low efficiency that could not be quantified.^66^ Recent studies for Fe^III^-perylene dyads reveal an unusual doublet-triplet annihilation between the ^2^LMCT excited state and the triplet state of anchored perylene, which leads to upconversion luminescence in the absence of additional annihilators.^29,105^ For Cr_2_-An, no delayed fluorescence from ^1^PA* is observed under irradiation from a 520 nm CW-laser (SI, Figure S15), which excludes the occurrence of doublet-triplet annihilation or triplet–triplet annihilation at homomolecular level under our conditions. A comparative TTA-UC study with Cr_2_-ref could not be performed, due to the very limited solubility of the complex in DCE.

### ^1^O_2_-Mediated Photocatalysis

Since the phosphorescence from Cr_2_-An is sensitive to oxygen and sufficiently long-lived for diffusion-controlled bimolecular reactions, we investigated this complex for benchmark singlet oxygen (^1^O_2_)-mediated photocatalytic oxidations of α-terpinene and 1-naphthol^28,74^ with visible light and compared the photocatalytic performance with Cr_2_-ref (Figure 8a,b). The quenching rate constant *k*_q_ by molecular oxygen is estimated to 2.5 × 10^9^ M^–1^ s^–1^ for Cr_2_-An in acetonitrile at room temperature, substantially faster than that obtained for Cr_2_-ref (4.6 × 10^7^ M^–1^ s^–1^) (SI, Sections 6.1 and 6.2), likely due to the distinct nature of ^3^PA* in Cr_2_-An and the metal-centred excited states in Cr_2_-ref.^66^ For Cr_2_-An, the quantum yield Φ_Δ_ for photosensitized generation of ^1^O_2_ was determined to 29% in aerated acetonitrile at room temperature, whereas for Cr_2_-ref this is found very low (Φ_Δ_ < 0.5%) (SI, Sections 6.1 and 6.2). This leads to high product yields of 74–83% for both photocatalytic reactions using Cr_2_-An as the photosensitizer, whereas significantly lower yields were obtained for Cr_2_-ref with the same catalyst amount under identical conditions. Photoinduced electron transfer with the substrates is energetically highly uphill by 0.37 to 0.58 eV for both dimers and therefore thermodynamically not feasible, as evidenced by the quenching experiments (SI, Section 10.4).

**Figure 8:**
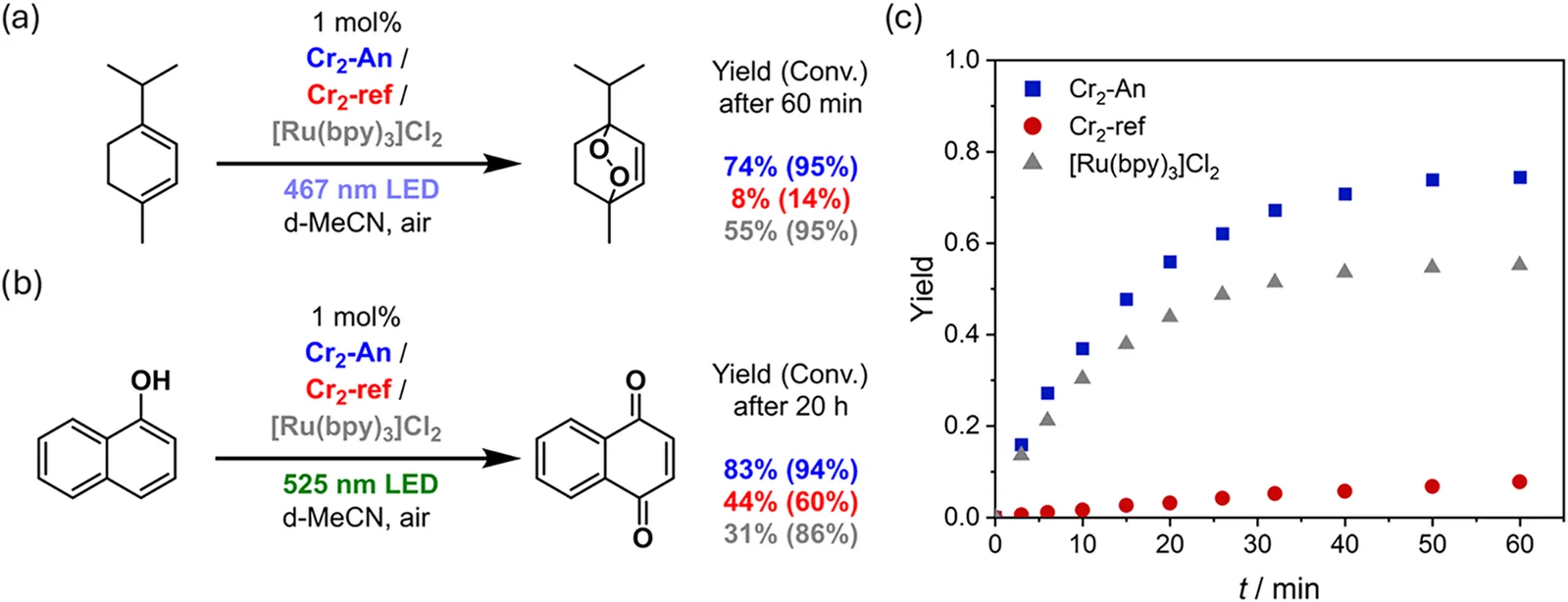
Photocatalytic reactions mediated by ^1^O_2_. Reaction schemes for photocatalytic oxidation of (a) α-terpinene under 467 nm LED irradiation and (b) 1-naphthol under 525 nm LED irradiation. The reactions are catalysed either by Cr_2_-An (blue), Cr_2_-ref (red), or [Ru­(bpy)_3_]­Cl_2_ (gray) under identical conditions. (c) Reaction kinetics obtained for photocatalytic oxidation of α-terpinene from (a) in the presence of the indicated catalysts. The product yields were obtained from NMR measurements (SI, Section 10).

Considering that [Ru­(bpy)_3_]­Cl_2_ and Cr_2_-An have similar extinction coefficients in the visible range from 460 to 550 nm (SI, Figure S31) and a triplet excited state lifetime of ∼100 ns in aerated acetonitrile,^127^ we further compared the photocatalytic performance between these two photosensitizers under identical conditions by following their reaction kinetics using NMR measurements (SI, Section 10). For the photooxidation of α-terpinene, Cr_2_-An outperforms [Ru­(bpy)_3_]­Cl_2_ with respect to the reaction kinetics and product yields under identical conditions (Figure 8c). For the photooxidation of 1-naphthol, Cr_2_-An shows highly consistent yields for product formation and substrate conversion, whereas only 31% product yield was obtained for [Ru­(bpy)_3_]­Cl_2_ despite a rapid substrate conversion, likely due to side reactions based on electron transfer with the formed 1,4-naphthoquinone.^128^ The superior photocatalytic performance for Cr_2_-An over [Ru­(bpy)_3_]­Cl_2_ makes it a highly attractive photosensitizer based on earth-abundant transition metal.

## Conclusion

Luminescent mononuclear Cr^III^ complexes are attractive chromophores for photochemical conversion based on electron- or energy transfer.^17,34,45,46^ However, their inherently weak d–d absorption in the visible range remains a fundamental bottleneck for efficient light harvesting. This is also true for dinuclear Cr^III^ complexes, which are mostly non-emissive at room temperature and scarcely applied for photochemical conversion. Our study presents two novel dinuclear Cr^III^ complexes that show room temperature luminescence and ferromagnetic coupling in their ground state. Combination of luminescence and high spin state (*S* = 3) makes these Cr^III^ dimers potentially attractive candidates for optically addressable molecular qubits.^129,130^ By attaching two phenyl anthracenes (PAs) to a dinuclear Cr^III^ complex, strong visible absorption from a unique π–π* transition of a laterally offset, π-stacked anthracene pair is accessed in our Cr_2_-An. The extinction coefficient at 450 nm, ε_450_, achieves a value of 7570 M^–1^ cm^–1^ for Cr_2_-An, which exceeds that for Cr_2_-ref by a factor of 30. This validates the “antenna effect” for enhancing visible light harvesting of Cr^III^ complexes, which is fundamentally relevant for the development of efficient photosensitizers based on earth-abundant metals.

Room temperature phosphorescence from anthracenes is usually accessed in the solid state, by heavy atom effect^94^ or in confined environments,^93,131^ whereas examples in homogeneous solution remain scarce.^108^ For Cr_2_-An, room temperature NIR-phosphorescence from ^3^PA* with a lifetime of ∼150 ns, which is sensitive to oxygen, is observed in solution. Two potential pathways drive the formation of ^3^PA*: direct ISC from the ^1^(PA)_2_* state, and DTET from the spin-flip excited states of the Cr^III^ centers. The unusual emissive nature of ^3^PA* is likely attributed to the combined effect of the rigid arrangement of the two PA units and the presence of the chromium­(III) ions. This provides a powerful molecular design strategy to achieve room temperature phosphorescence of organic chromophores in solution, a phenomenon that is otherwise highly challenging to realize.

The strong visible absorption, the long-lived phosphorescent excited state, and the high photostability make Cr_2_-An an attractive photosensitizer for energy-transfer based applications. Using AnTIPS as the annihilator, Cr_2_-An sensitizes green-to-blue upconversion with a quantum yield of up to 1.1% (theoretical maximum set to 50%), yielding delayed fluorescence with microsecond-scaled time constants. Under aerobic conditions, Cr_2_-An sensitized ^1^O_2_-mediated oxidations of α-terpinene and 1-naphthol with visible light, giving high product yields of up to 83%. Superior photocatalytic performance is observed for Cr_2_-An compared to Cr_2_-ref or the benchmark [Ru­(bpy)_3_]­Cl_2_. This makes our dinuclear Cr^III^ complex attractive in the context of efficient and sustainable photochemical conversion.

## Supplementary Material



## Notes

Synthetic protocols and characterization data for both complexes and organic chromophores, description of equipment and methods, detailed experimental section and additional experiments, spectroscopic data, and supplementary discussions (PDF)

## ⊥

Institute of Functional Interfaces (IFG), Karlsruhe Institute of Technology (KIT), Hermann-von-Helmholtz-Platz 1, 76344 Eggenstein-Leopoldshafen, Germany
