# Supplementary material for: Visible Light-Harvesting Dinuclear Chromium(III) Complex with Anthracene-Based Phosphorescence for Photon Upconversion and Photocatalysis
Source: J Am Chem Soc. 2026 Oct 2;148(40):43469–81. doi: 10.1021/jacs.6c13239 (PMC13674269; doi:10.1021/jacs.6c13239)
Supplement: Supplementary file 1 [file ja6c13239_si_001.pdf]

Supporting information

Visible Light-Harvesting Dinuclear Chromium(III) Complex with Anthracene-Based Phosphorescence for Photon Upconversion and Photocatalysis

Dennis Fritzler,[a] Katharina L. Deuter,[a] Nikita Vashistha,[b] Serhiy Demeshko,[c] Michael Linseis,[a] Pengyue Jin,[a] Franc Meyer,[c] Benjamin Dietzek-Ivanšić,[b,d] Rainer F. Winter,[a] Cui Wang [a]*

[a] Department of Chemistry, University of Konstanz, Universitätsstraße 10 78464 Konstanz, Germany.
[b] Leibniz Institute of Photonic Technology, e. V. Jena, Albert-Einstein-Straße 9, 07745 Jena, Germany
[c] Institute of Inorganic Chemistry, University of Göttingen, Tammannstraße 4,37077 Göttingen, Germany.
[d] Leibniz Institute of Surface Engineering (IOM), Permoserstraße 15, 04318 Leipzig, Germany

Table of contents

1. Materials and methods 3
2. Synthesis and characterization 5
2.1 Synthesis of 2,6-di(quinolin-8-yl)pyridine (dqp) 5
2.2 Synthesis of 2,6-dichloro-4-(10-phenylanthracen-9-yl)pyridine (Py-PA) 5
2.3 Synthesis of 8,8’-(4-(10-phenylanthracen-9-yl)pyridine-2,6-diyl)diquinoline (dqp-PA) 6
2.4 Synthesis of Cr_2-ref 7
2.5 Synthesis of Cr_2-An 8
2.6 Synthesis of 9,10-Bis((triisopropylsilyl)ethynyl)anthracene (AnTIPS) 9
3. Single crystal X-ray diffraction analysis 9
4. Magnetic properties 14
5. Electrochemistry 15
6. Photophysical characterization 15
6.1 Cr_2-ref 15
6.2 Cr_2-An 18
7. Density Functional Theory calculations 23
8. Transient absorption spectroscopy 33
8.1 fs-transient absorption spectroscopy 33
8.2 ns-transient absorption spectroscopy 36
9. Triplet-triplet annihilation upconversion 38
9.1 Stern-Volmer studies 38
9.2 Control experiments 38
9.3 Upconversion experiments 39
10. Photocatalysis via $^1O_2$ generation 42
10.1 General 42
10.2 Oxidation of 1-naphthol 43
10.3 Oxidation of α-terpinene 48
10.4 Direct quenching of photocatalysts by substrates 52
11. NMR spectra 54
12. References 57

1. Materials and methods

Reagents for the synthesis of ligands and complexes were obtained from various suppliers (Sigma-Aldrich, Thermo Fisher Scientific, abcr, Carl Roth, BLDpharm, Apollo Scientific) and used as received. Solvents for spectroscopic measurements were purchased as "extra dry" from Thermo Fisher Scientific or Sigma-Aldrich and were filtered prior to use. Deuterated solvents for NMR spectroscopy were obtained from deutero. $[Ru(bpy_3]Cl_2 \times 6H_2O$ used as a reference for quantum yield measurements and photocatalytic reactions was purchased from abcr.

NMR spectra were measured on a Bruker DPX-250 (B_H = 250 MHz), Bruker Avance III-500 (B_H = 500 MHz), Bruker Avance III-400 (B_H = 400 MHz) or Jeol Resonance ECZ 400S (B_H = 400 MHz) spectrometer. ^{1}H-NMR spectra were recorded with the indicated proton frequencies. ^{13}C-NMR spectra were measured on the Bruker Avance III-500 spectrometer operating at 125 MHz. Chemical shifts were referenced to the residual protonated solvent signals. Coupling constants J are given in Hertz (Hz) with the coupling patterns being abbreviated as: s (singlet), d (doublet), t (triplet), q (quartet), m (multiplet) and a combination thereof.

Elemental analysis was performed on a C,H,N-combustion analyzer (Elementar Unicube).

Mass spectrometry was measured with 10^{-6} M solutions of the analytes in acetonitrile on a LTQ-Orbitrap mass spectrometer (Thermo Fisher Scientific). The samples were diluted 1:10 with DCM and directly injected into the spectrometer with a flow rate of 5 µl/min. The data were acquired over 2-5 min. Spectra were visualised using Xcalibur (Thermo Fisher Scientific).

Single crystal X-ray diffraction (SC-XRD) for **Cr_2-An** was conducted on a Bruker Kappa APEX II CCD-based 4-circle diffractometer with graphite-monochromated Mo K_α radiation (λ = 0.71073 Å) at 100 K. Data processing and semi-empirical absorption correction were done with the Bruker APEX2 software package. **Cr_2-ref** was measured on a STOE IPDS II diffractometer with graphite monochromated Mo K_α radiation (λ = 0.71073 Å) and an image plate detection system at 100 K. Data processing was performed with the X-Area software package version 2.8 including Integrate3D GUI v1.3.23.0 (STOE & Cie GmbH). Absorption correction was done with X-Red32 by Gaussian integration[1] and scaling of reflection intensities was performed within STOE LANA.[2] Final spherical absorption correction was done within STOE LANA. For both dimers, structure solution was performed with the OLEX2[3] program package with SHELXT[4] using intrinsic phasing and refined with the SHELXL[5] software package using Least Squares minimization. Hydrogen atoms were introduced at their calculated positions, except for hydrogen atoms on oxygen atoms engaged in hydrogen bridging, which were refined using Least Squares minimization.

Temperature-dependent magnetic susceptibility measurements were performed on a Quantum-Design MPMS3 SQUID magnetometer equipped with a 7 T magnet in the temperature range from 295 K to 2 K at a magnetic field of 0.5 T. The powdered samples were contained in a polycarbonate capsule and fixed in a non-magnetic sample holder. Each raw data file for the measured magnetic moment was corrected for the diamagnetic contribution of the polycarbonate capsule according to M^{dia}(capsule) = $\chi_g \cdot m \cdot H$, with an experimentally obtained gram susceptibility of the polycarbonate capsule. The molar susceptibility data were corrected for the diamagnetic contribution. Experimental data were modelled with the *julX* program[6] using a fitting procedure to the spin Hamiltonian $\hat{H} = -2J\hat{S}_1\hat{S}_2 + g\mu_B\vec{B}(\vec{S}_1 + \vec{S}_2)$. Temperature-independent paramagnetism (TIP = $270 \cdot 10^{-6}$ cm^{3}mol^{-1} for **Cr_2-ref** and $870 \cdot 10^{-6}$ cm^{3}mol^{-1} for **Cr_2-An)** was included according to $\chi_{exp} = \chi_{dimer} + TIP$.

Cyclic voltammetry was measured with 1 mM solutions of the analyte in dry deaerated DMF with 100 mM tetra-*n*-butylammonium hexafluorophosphate ($TBAPF_6$) as the electrolyte. The data was

acquired with a computer-controlled BASi potentiostat with a scan rate of 100 mV/s. A glassy carbon disc was used as the working electrode, with a platinum wire and a saturated calomel electrode serving as counter and reference electrodes.

UV-Vis absorption spectra were recorded on a Varian Cary 6000 or Edinburgh Instruments DS5 spectrometer in 10×10 mm standard or long neck quartz cuvettes.

Steady-state and time-resolved luminescence measurements were conducted on a Horiba Hobin-Yvon Fluorolog-3, a PicoQuant FluoTime 300 or an Agilent Cary Eclipse spectrometer, which are equipped with a xenon lamp and external lasers (PicoQuant LDH-D-C-375, LDH-D-C-450 or LDH-D-C-485; RGB Lasersystems LambdaBeam 520 (121 mW) or LambdaBeam 532 (200 mW)) as the light sources. Luminescence decay measurements were conducted on the FluoTime 300 spectrometer using either time-correlated single photon counting (TCSPC) technique or a photomultiplier tube (PMT) detector. If necessary, a suitable longpass filter was placed between the sample and the detector to avoid second order scattering artifacts in the spectra. Photoluminescence quantum yields Φ_{PL} were determined relative to [Ru(bpy$_3$]Cl_2 according to equation S1.

$$\Phi_{\mathrm{PL}} = \Phi_{\mathrm{ref}} \cdot \frac{A_{\mathrm{ref}}}{A_{\mathrm{s}}} \cdot \frac{I_{\mathrm{s}}}{I_{\mathrm{ref}}} \cdot \left(\frac{\eta_{\mathrm{s}}}{\eta_{\mathrm{ref}}}\right)^2 \qquad eq.(S1)$$

A_{ref} and A_s represent the absorbance of the reference and the samples, I_{ref} and I_S indicate the integrated emission intensities of the reference and the samples, η_{ref} and η_S represent the refractive indices of the solvents used for the reference and the sample, respectively. The calculation is based on the literature-known luminescence quantum yield Φ_{ref} of the reference sample [Ru(bpy)$_3$]Cl_2 (Φ_{ref} = 6.3% in deaerated water).[7]

Quantum chemical calculations based on density functional theory (DFT) employed the Gaussian 16 program package.[8] Time-dependent DFT (TD-DFT) calculations and vibrational analyses were preceded by geometry optimization for **Cr_2-ref**. For TD-DFT calculations on the dimeric anthracenyl fragment, the coordinates were obtained from the single-crystal XRD structure of **Cr_2-An**. All calculations were performed with the B3LYP functional, [9-12] the 6-311G basis set for the lighter elements (C, N, H, O), a pseudopotential on Cr, [13] and applying the self-consistent reaction field model (SCRF) in the solvation model based on density (SMD) variation in acetonitrile, [14] while omitting the corresponding anions. The spin state of **Cr_2-ref** was assumed to be a septet, while, for the anthracene fragments, a singlet state was used. Electronic spectra were calculated from the described structures by the TD-DFT method. GaussSum,[15] Avogadro,[16] GNU Parallel,[17] and vmd[18] program packages were used in combination with POV-Ray[19] for data processing and graphical representations.

Femtosecond transient absorption (fs-TA) measurements in the visible region were performed using a Ti:sapphire-based regenerative amplifier system (Astrella Coherent Inc.), which generated 800 nm pulses at a repetition rate of 1 kHz. This fundamental beam is split to generate pump and probe pulses. The samples were excited using 370 nm and 460 nm pump pulses generated by an optical parametric amplifier (OPA) (Topas-prime, Light conversion). Transient absorption spectra were collected using a white-light continuum probe generated by focusing the 800 nm beam into a CaF_2 crystal. Data processing and analysis were performed using the Python-based KiMoPack package.[20]

UV-Vis nanosecond transient absorption spectroscopy was conducted on a LP920-KS spectrometer from Edinburgh Instruments, which is equipped with a pulsed xenon lamp as the probe light source. A frequency-tripled pulsed Nd:YAG laser (Quantel Q-smart 450, ca. 10 ns

pulse width) with a beam expander (Thorlabs BE02-355) in the optical path was used for experiments that required excitation at 355 nm. For excitation at 532 nm, a frequency-doubled pulsed Nd:YAG laser (Quantel Q-smart 450, ca. 10 ns pulse width) was used with a beam expander (Thorlabs GBE02-A) in the optical path. The transient absorption spectra were captured on an iCCD camera from Andor, and the kinetics at chosen wavelengths were measured with a photomultiplier tube.

2. Synthesis and characterization

2.1 Synthesis of 2,6-di(quinolin-8-yl)pyridine (dqp)

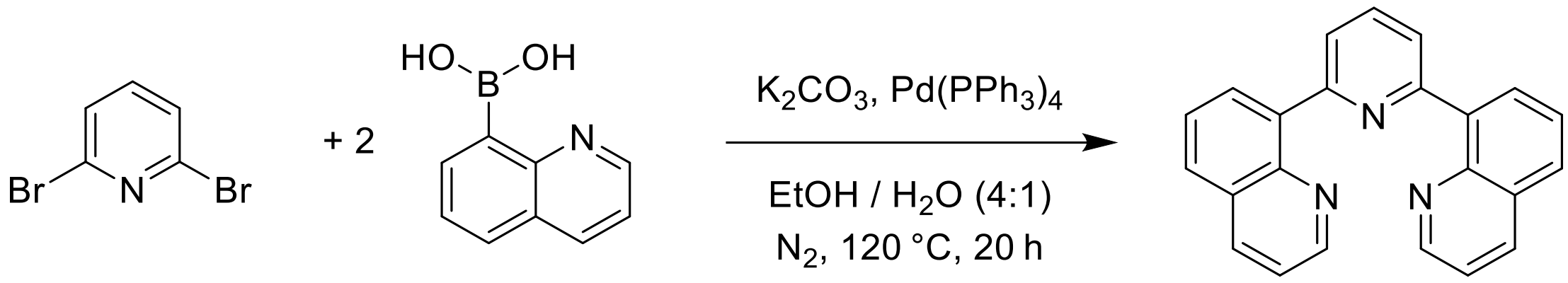

The synthesis of the ligand dqp was adapted from a previously published procedure.[21] 2,6-Dibromopyridine (1059 mg, 4.47 mmol), 8-quinolineboronic acid (1935 mg, 11.2 mmol), potassium carbonate (2471 mg, 17.9 mmol) and tetrakis(triphenylphosphine)palladium (104 mg, 0.09 mmol) were charged into a microwave vial. A mixture of deaerated ethanol and water (12 mL, 4:1) was then added and the vial was capped. The suspension was heated to 120 °C for 20 h under continuous stirring. The reaction mixture was cooled to room temperature and filtered over Celite. The residue was washed with DCM and the resulting filtrate was dried over anhydrous sodium sulfate. The solvent was removed under reduced pressure, leaving a yellow-orange residue. The residue was suspended in 50 mL of ethanol and stored at 4 °C for 2 h. The solution was filtered through a paper filter and the solid residue was washed with cold ethanol until the residue was colorless. The solid was collected and dried at 60 °C, giving dqp as an off-white powder (1.28 g, 86% yield). ^{1}H measurement of the obtained dqp is in line with previous literature reports. [21]

^{1}H NMR (250 MHz, CD_3Cl) δ [ppm] 9.03 (dd, *J* = 4.2, 1.9 Hz, 2H), 8.28 (ddd, *J* = 10.1, 7.7, 1.7 Hz, 4H), 8.16 (d, *J* = 7.4 Hz, 2H), 8.02 – 7.87 (m, 3H), 7.69 (dd, *J* = 8.2, 7.2 Hz, 2H), 7.47 (dd, *J* = 8.3, 4.2 Hz, 2H).

2.2 Synthesis of 2,6-dichloro-4-(10-phenylanthracen-9-yl)pyridine (Py-PA)

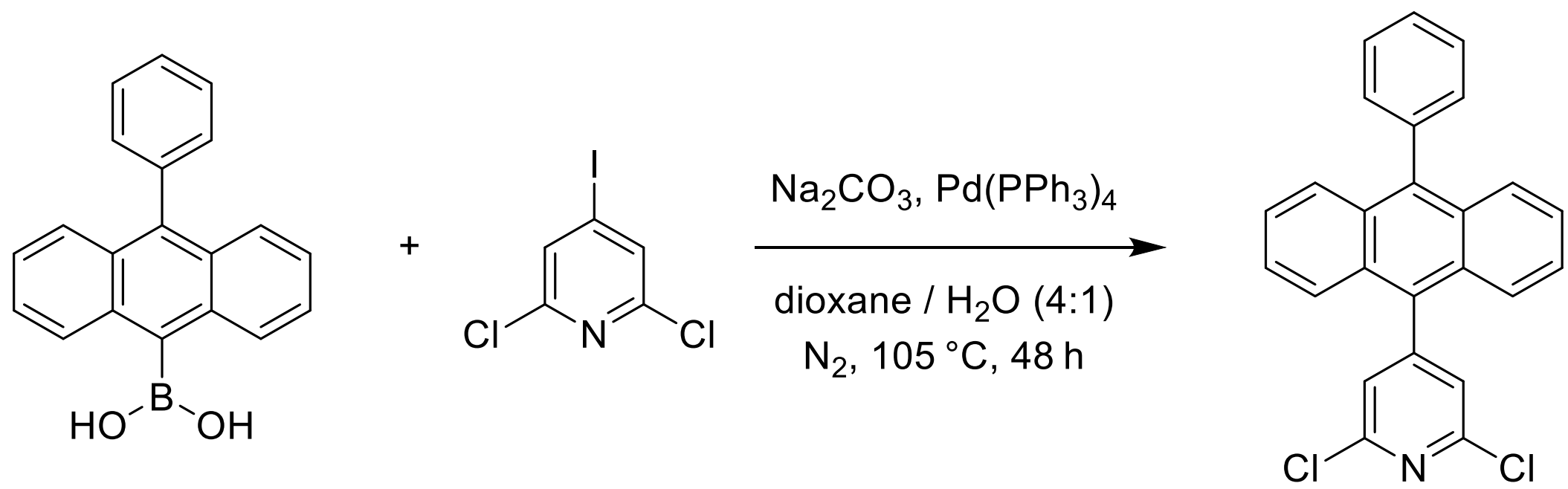

The ligand precursor Py-PA was synthesized via Suzuki-coupling in accordance to previously published literature.[22] 2,6-Dichloro-4-iodopyridine (260 mg, 0.93 mmol), (10-phenylanthracen-9-yl)boronic acid (260 mg, 0.87 mmol), sodium carbonate (300 mg, 2.83 mmol) and tetrakis(triphenylphosphine)palladium (108 mg, 0.093 mmol) were charged into a Schlenk tube

and sealed with a septum. The solid mixture was evacuated and flushed with nitrogen for three cycles. A solvent mixture of 1,4-dioxane and water (8 mL, 4:1) was purged with nitrogen for 30 min and then transferred to the solid reaction mixture under nitrogen atmosphere. The suspension was dispersed by sonication and heated to 105 °C under continuous stirring for 48 h. The reaction mixture was cooled to room temperature and filtered through celite. The residue was washed with dichloromethane (DCM) and the filtrate was extracted with water twice. The aqueous phases were extracted with DCM and the combined organic phases were dried over anhydrous sodium sulfate. The solvent was removed under reduced pressure, leaving a dark orange residue. The residue was washed with small amounts (4 mL) of a cyclohexane/ethyl acetate mixture (9:1) until no orange color remained in the residue. The resulting solid was dried under reduced pressure, giving Py-PA as pale yellow crystals (226 mg, 65% yield). ^{1}H and ^{13}C NMR measurements of the obtained Py-PA are in line with the literature report.[22]

^{1}H NMR (250 MHz, CD_3Cl) δ [ppm] 7.75 (d, *J* = 7.7 Hz, 2H), 7.67 – 7.56 (m, 5H), 7.51 – 7.36 (m, 8H).

^{13}C NMR (125 MHz, $CDCl_3$) δ [ppm] 153.65, 151.03, 139.31, 138.35, 131.07, 130.58, 129.74, 128.89, 128.52, 127.83, 127.45, 126.40, 125.65, 125.37, 125.27.

2.3 Synthesis of 8,8'-(4-(10-phenylanthracen-9-yl)pyridine-2,6-diyl)diquinoline (dqp-PA)

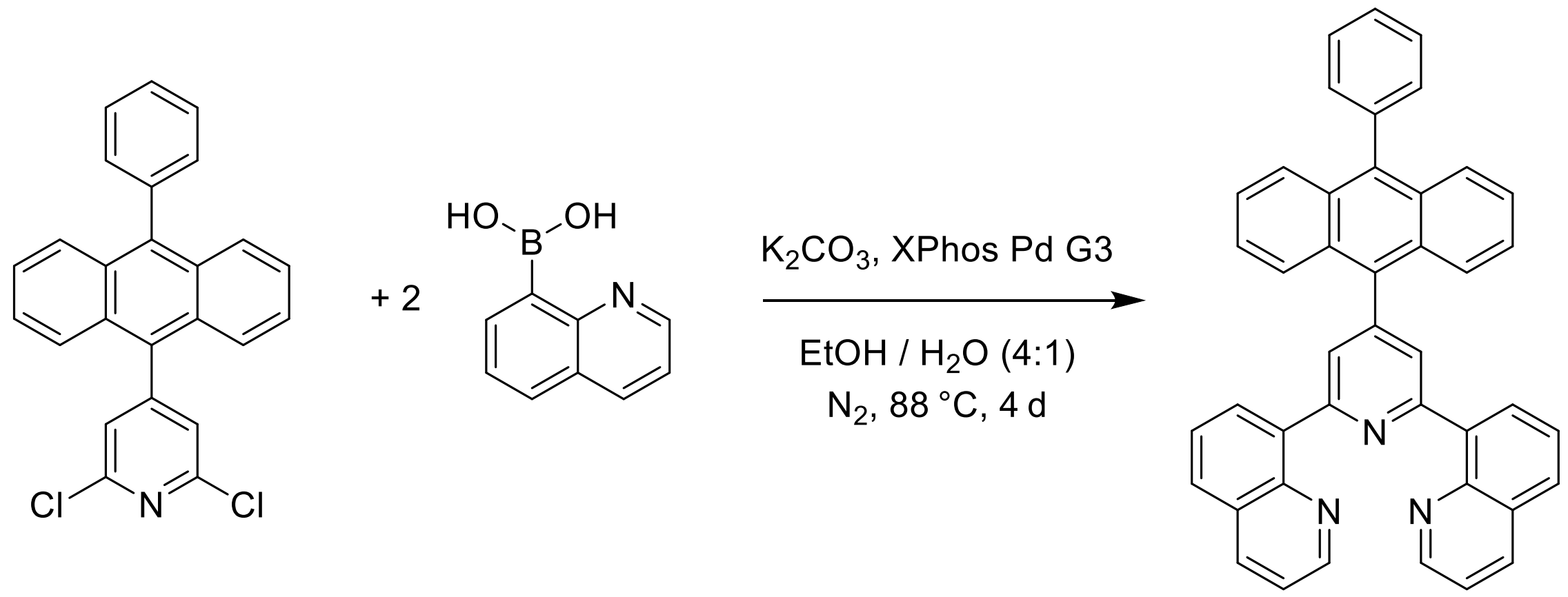

The ligand dqp-PA was synthesized via Suzuki-coupling in accordance to previously published literature.[22] Py-PA (226 mg, 0.56 mmol) obtained in the previous step, 8-quinolineboronic acid (208 mg, 1.2 mmol), potassium carbonate (343 mg, 2.48 mmol) and the palladium catalyst XPhos Pd G3 (22 mg, 0.026 mmol) were introduced into a Schlenk tube and sealed with a septum. The solid mixture was evacuated and flushed with nitrogen for three cycles. A solvent mixture of ethanol and water (4 mL, 4:1) was purged with nitrogen for 30 minutes and then transferred to the reaction mixture under nitrogen atmosphere. The suspension was dispersed by sonication and heated to 88 °C under continuous stirring for 4 d. The reaction mixture was cooled down to room temperature and filtered through celite. The residue was washed with 120 mL of DCM and the filtrate was extracted with water twice. The aqueous phases were extracted with DCM and the combined organic phases were dried over anhydrous sodium sulfate. The solution was concentrated under reduced pressure until 5 mL of the solution remained. The solution was cooled in an ice bath and 20 mL of cold diethyl ether were added slowly, leaving a dark yellow-green precipitate. The precipitate was removed by paper filtration and washed with cold diethyl ether until no green color remained. The remaining residue was dried under reduced pressure giving dqp-PA as lightly yellow crystals (248 mg, 75% yield). ^{1}H and ^{13}C NMR measurements of the obtained dqp-PA are in agreement with the literature report.[22]

^{1}**H NMR** (500 MHz, CD_2Cl_2) δ [ppm] 8.98 (dd, *J* = 4.1, 1.9 Hz, 2H), 8.55 (dd, *J* = 7.2, 1.5 Hz, 2H), 8.43 (s, 2H), 8.39 (d, *J* = 8.8 Hz, 2H), 8.31 (dd, *J* = 8.3, 1.8 Hz, 2H), 8.00 (dd, *J* = 8.1, 1.5 Hz, 2H), 7.83 – 7.74 (m, 4H), 7.70 – 7.65 (m, 2H), 7.64 – 7.60 (m, 1H), 7.57 – 7.51 (m, 4H), 7.47 (dd, *J* = 8.2, 4.1 Hz, 2H), 7.44 (ddd, *J* = 8.8, 6.5, 1.3 Hz, 2H).

^{13}**C NMR** (125 MHz, CD_2Cl_2) δ [ppm] 156.54, 150.29, 148.83, 146.64, 139.14, 137.98, 137.81, 137.57, 136.23, 135.07, 131.24, 130.01, 129.40, 128.93, 128.75, 128.71, 128.42, 127.54, 127.02, 126.97, 126.39, 125.52, 125.16, 121.03.

2.4 Synthesis of Cr_2-ref

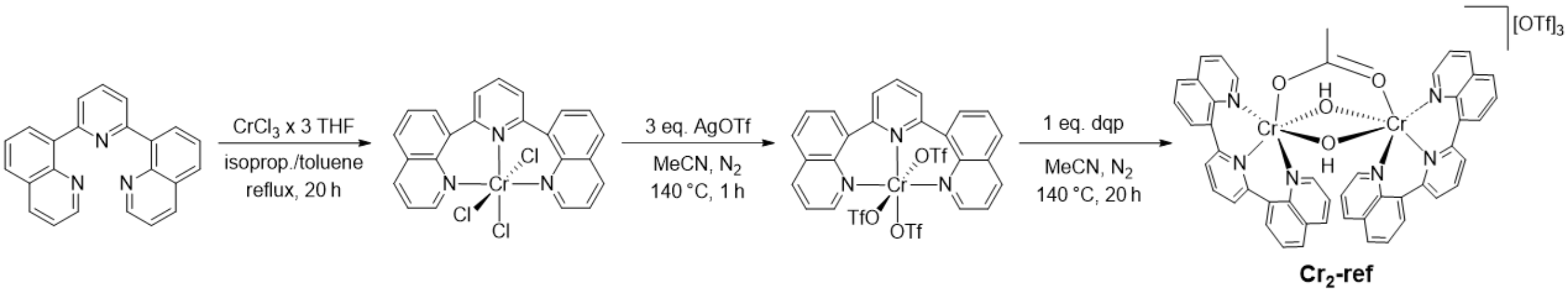

Cr_2-ref was synthesized by adapting a reported procedure, in which the bridging acetate and hydroxides are likely formed from acetonitrile and water traces under the harsh reaction conditions.[23] In a round-bottom flask, $CrCl_3$ × 3 THF (375 mg, 1 mmol) and the ligand dqp (333 mg, 1 mmol) were dispersed in a solvent mixture of isopropanol and toluene (80 mL, 1:1). The mixture was heated to reflux under continuous stirring for 20 h before being cooled to room temperature. The resulting green precipitate was removed by paper filtration and washed with warm ethanol (30 mL), DCM (15 mL) and diethyl ether (15 mL). The resulting green solid was dried under reduced pressure, giving $Cr(dqp)Cl_3$ (400 mg, 81% yield).

The obtained $Cr(dqp)Cl_3$ (175 mg, 0.35 mmol) and silver triflate (273 mg, 1.06 mmol) were introduced into a microwave vial that was sealed with a septum cap under inert conditions. After adding 6 mL of deaerated acetonitrile with a syringe, the mixture was heated to 140 °C under continuous stirring for 1 h, leaving a white precipitate in a brown solution. This solution was used in the next step without isolation of the intermediate.

One equivalent of the ligand dqp (117 mg, 0.35 mmol) was added to a microwave vial that was sealed under inert conditions. The brown solution from the previous step was transferred to the new vial with a syringe and filtered through a syringe filter. The remaining white precipitate was washed with 4 mL of deaerated acetonitrile. The washing solution was also transferred to the new vial. The mixture was heated to 140 °C for 20 h under continuous stirring, leaving a red solution with a pale orange precipitate after cooling to room temperature. The precipitate was removed by paper filtration and washed with cold acetonitrile (20 mL) and cold water (20 mL). The residue was transferred to a vial and dispersed in 5 mL of methanol. After 5 min of stirring, the orange methanol solution was transferred to a flask through a syringe filter. This step was repeated twice until the remaining residue became colorless. The solvent was then removed from the solution under reduced pressure, leaving an orange solid in the flask. This was dissolved in 2 mL of DMF and transferred into a new vial. This was then used for vapor diffusion crystallization with diethyl ether at 4 °C. The crystals were collected after 4 days and washed with diethyl ether giving **Cr_2-ref** as an orange crystalline powder (42 mg, 18% yield). Crystals suitable for SC-XRD analysis were obtained through slow evaporation of the compound from methanol.

ESI-MS (m/z): calc. for $[Cr_2(\mu\text{-}OH)_2(\mu\text{-}CH_3CO_2)(dqp)_2]^{3+}$: 287.7171, found: 287.7227

Elemental analysis: calculated ($Cr_2C_{51}H_{35}N_6S_3F_9O_{13}$ × 1.5 H_2O × 2.5 DMF × 0.5 Et_2O): C, 46.65%; H, 3.91%; N, 7.64%; found: C, 46.50%; H, 3.78%; N, 7.56%.

2.5 Synthesis of Cr_2-An

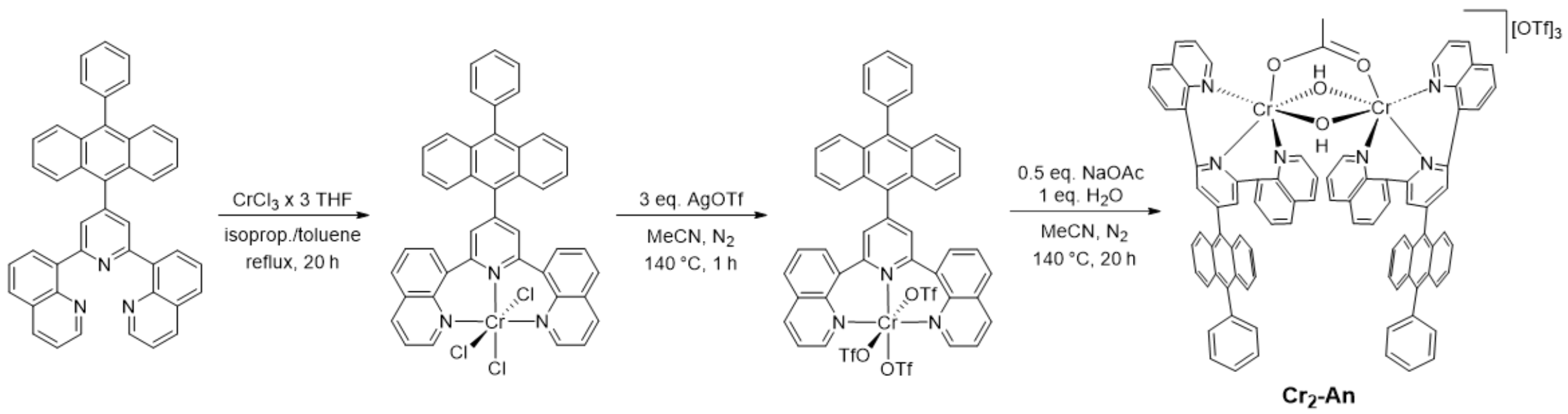

Cr_2-An was synthesized from the same Cr(III) precursor by adapting a literature procedure.[23] $CrCl_3$ × 3 THF (127 mg, 340 µmol) and the ligand dqp-PA (199 mg, 340 µmol) were weighted into a round-bottom flask and dispersed in a solvent mixture of isopropanol and toluene (50 mL, 1:1). The reaction mixture was heated to reflux and stirred for 20 h, leaving a light green precipitate after cooling to room temperature. The precipitate was removed by paper filtration and washed with warm ethanol (20 mL), DCM (10 mL) and diethyl ether (10 mL). The resulting yellow-green solid was dried under reduced pressure, giving Cr(dqp-PA)Cl_3 (153 mg, 58% yield).

The obtained Cr(dqp-PA)Cl_3 (153 mg, 200 µmol) and silver triflate (154 mg, 600 µmol) were introduced into a microwave vial that was sealed with a septum cap. The solid mixture was evacuated and flushed with nitrogen for three cycles. Acetonitrile (8 mL) was purged with nitrogen for 30 minutes before being transferred into the microwave vial. The reaction mixture was heated to 140 °C for 1 h under continuous stirring, leaving a white precipitate in a dark brown solution. This intermediate was not isolated and directly used in the next step.

The last step was improved compared to the synthesis of **Cr_2-ref** by the use of sodium acetate and water instead of another equivalent of the polypyridine ligand, which lead to a higher product yield. For this, sodium acetate (8 mg, 100 µmol) was charged into another microwave vial under inert conditions. The vial was sealed with a septum cap and the brown solution from the previous step was transferred into the new vial with a syringe equipped with a syringe filter. The white precipitate left in the original vial was washed with 5 mL of deaerated acetonitrile. The washing solution was then also transferred to the new vial through a syringe with a filter under inert conditions. A small amount of deaerated water was also added to the reaction mixture (3.6 µL, 200 µmol), which was heated to 140 °C for 20 h under continuous stirring. The dark orange red solution was slowly cooled to 0 °C and opened to ambient air before being filtered through a syringe filter into multiple glass vials and washed with cold acetonitrile (3 mL). The vials were then used for vapor diffusion crystallization with diethyl ether at 4 °C, giving single crystals suitable for SC-XRD analysis. The crystals were collected after 4 days and washed with diethyl ether giving **Cr_2-An** as a red crystalline powder (43 mg, 25% yield).

ESI-MS (m/z): calc. for $[Cr_2(\mu\text{-}OH)_2(\mu\text{-}CH_3CO_2)(dqp\text{-}PA)_2]^{3+}$: 455.7797, found: 455.7803

Elemental analysis: calculated ($Cr_2C_{91}H_{59}N_6S_3F_9O_{13}$ × 3 H_2O × CH_3CN): C, 58.46%; H, 3.59%; N, 5.13%; found: C, 58.45%; H, 3.61%; N, 5.18%.

2.6 Synthesis of 9,10-Bis((triisopropylsilyl)ethynyl)anthracene (AnTIPS)

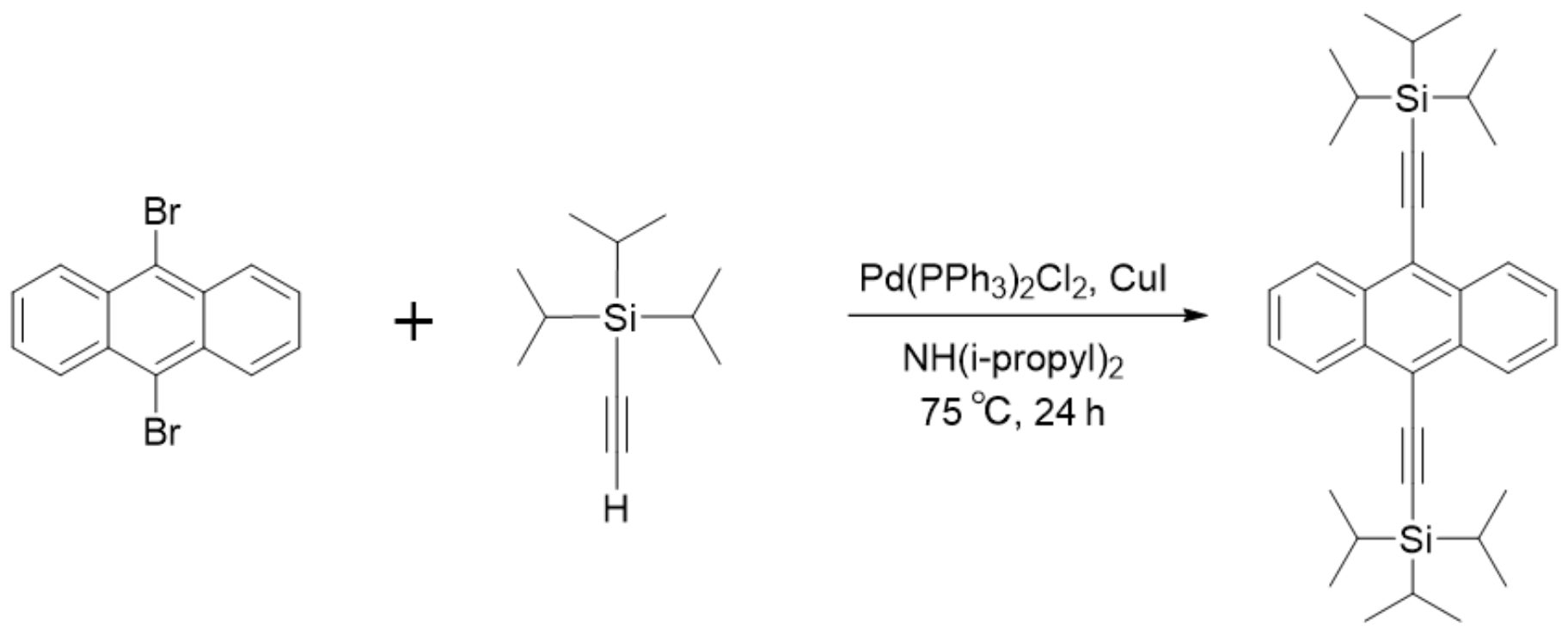

AnTIPS was synthesized by adapting previously reported procedures.[24, 25] 9,10-Dibromoanthracene (1.00 g, 2.99 mmol), CuI (46 mg, 0.24 mmol), and $Pd(PPh_3)_2Cl_2$ (84 mg, 0.12 mmol) (84 mg, 0.12 mmol) were placed in a Schlenk tube and degassed under vacuum for 30 min at room temperature. Deaerated $NH(i\text{-}propyl)_2$ (20 mL) and (triisopropylsilyl)acetylene (1.50 mL, 6.68 mmol) were added via syringe under nitrogen atmosphere. The reaction mixture was stirred at 75 °C overnight and cooled to room temperature. Upon treating with saturated aqueous NH_4Cl solution, the yellow precipitate was collected by filtration and washed with distilled water for three times. The crude product was purified by column chromatography on silica gel using petroleum ether as eluent to afford AnTIPS as a bright yellow solid (1.56 g, 95% yield). The obtained ^{1}H NMR spectrum is in line with the literature report of AnTIPS.[26]

^{1}H NMR (400 MHz, CD2Cl2) δ [ppm] 8.69 (dd, J = 6.7, 3.2 Hz, 4H), 7.67 (dd, J = 6.7, 3.2 Hz, 4H), 1.31 (d, J = 4.7 Hz, 42H).

3. Single crystal X-ray diffraction analysis

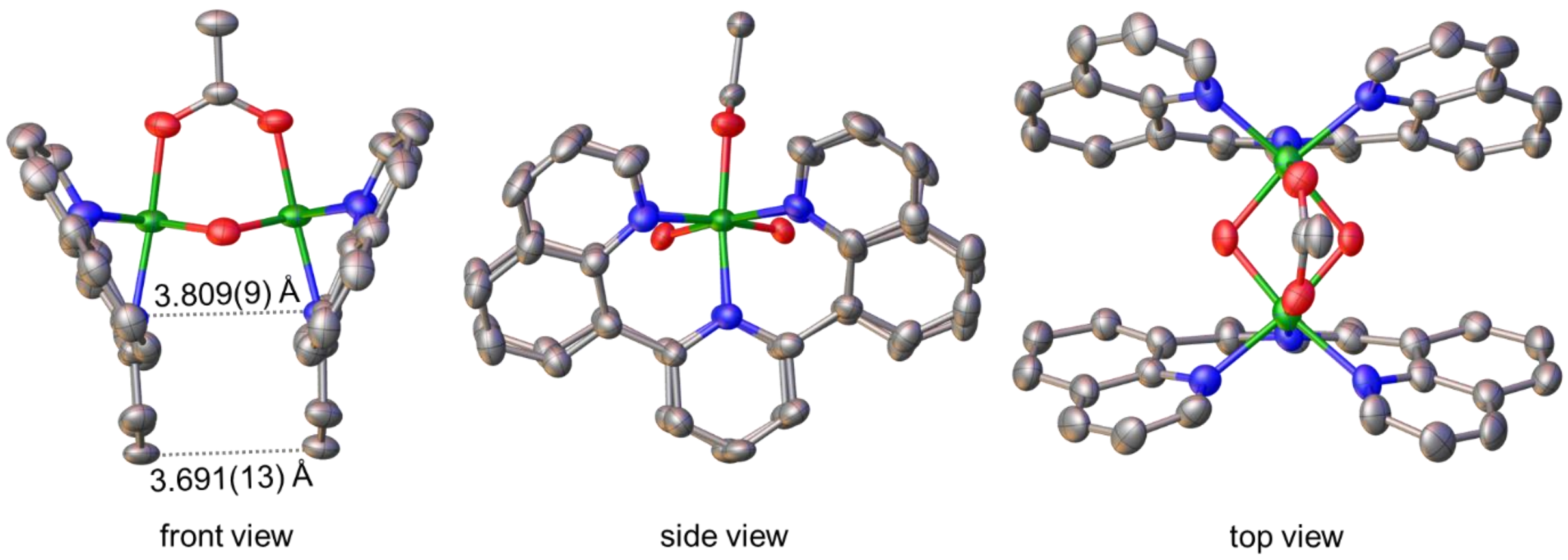

Figure S1: Structure of **Cr_2-ref** obtained from single crystal X-ray diffraction analysis, viewed from the front, side and top. The acetate bridge is shown in only one of two possible disordered positions. Counter ions, solvent molecules and H-atoms are omitted for clarity. Thermal ellipsoids are shown at 50% probability. Color code: Cr = green, N = blue, O = red, C = gray.

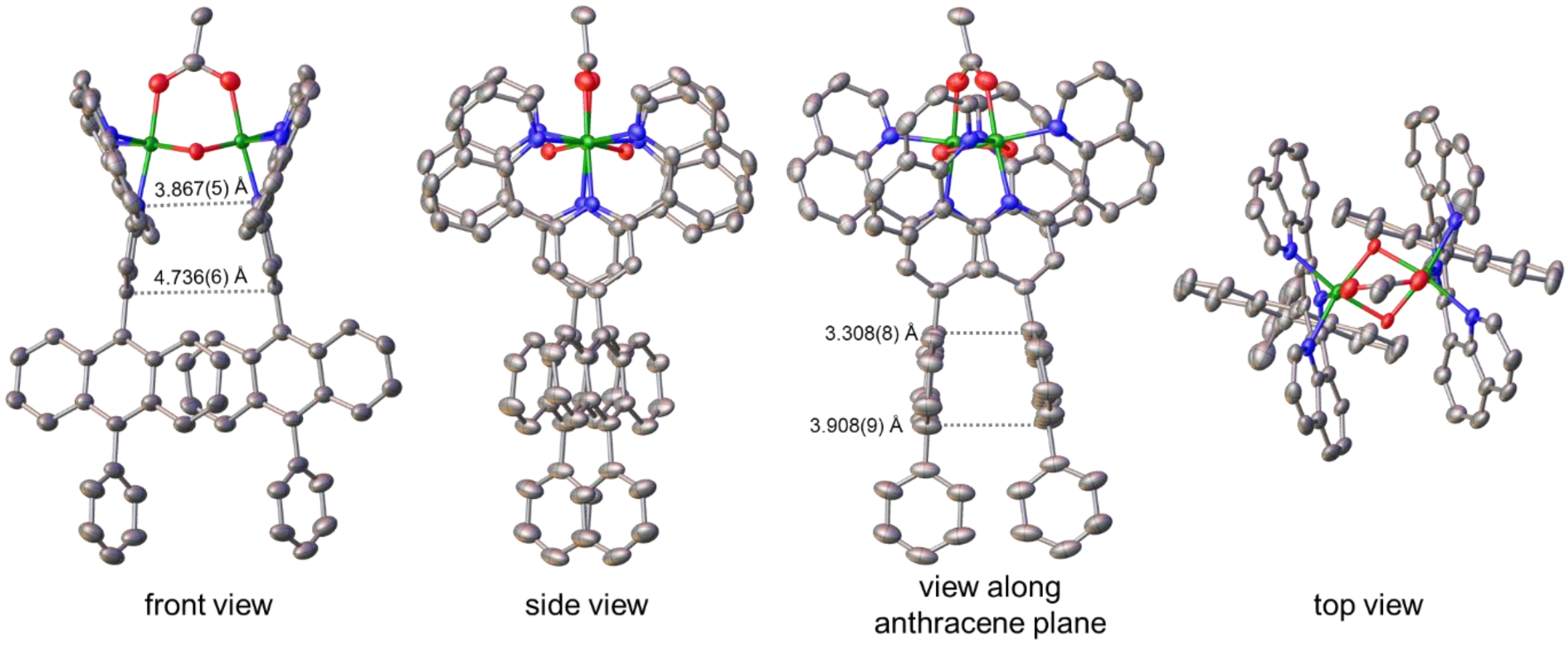

Figure S2: Structure of **Cr_2-An** obtained from single crystal X-ray diffraction analysis, viewed from the front, side, top and along the anthracene plane. The acetate bridge is shown in only one of two possible disordered positions. Counter ions, solvent molecules and H-atoms are omitted for clarity. Thermal ellipsoids are shown at 50% probability. Color code: Cr = green, N = blue, O = red, C = gray.

The structure of **Cr_2-ref** was solved as a twin with a contribution of only 1,8 % of the second domain. Two trifluoromethanesulfonate counterions are disordered over two different special positions in the triclinic space group P-1 and are solved in part -1 with an occupancy of 0.5 (one counterion per complex molecule). The other two trifluoromethanesulfonate counterions as well as the acetate ligand, two water and three methanol solvent molecules are disordered with almost equal contributions of 49.4% and 50.6%. All these solvent molecules, counterions, acetate and hydroxy ligands are connected in one complete hydrogen bridging system per part. The disordered counterions which are not on the special position could be refined with differently orientated CF_3 or SO_3 groups. This simplified description is reasonable, because a more sophisticated description would imply the usage of unnecessary restraints yielding a worse parameter-to-data ratio. The unaccounted additional disorder is the main reason for the high R values, with residual electron density around the Cr-centers being another reason. This can be attributed to an insufficient absorption correction. The Cr_2-units as well as the counterions with their sulfur atoms are orientated in one direction of the crystal, giving rise to a highly unsymmetric distribution of the electron density in the crystal. Restraints were placed only on the disordered solvent and counterions. Restrains were only necessary for the stable refinement of the hydroxy-ligand hydrogen atoms (DFIX) and the description of the acetate ligand disorder (SADI and RIGU). Overall two water and three methanol solvent molecules as well as three counterions where found per **Cr_2-ref** molecule in accordance with a assignment of a Cr(III) oxidation state.

In **Cr_2-An,** one disordered diethyl ether molecule per complex was removed from the refinement using the Olex2 solvent mask implementation of the Platon SQEEEZE method.[27] One water molecule is residing on a special position in the monoclinic space group C2/c. One of the trifluoromethanesulfonate counterions is disordered over a special position. The disorder is modelled by placing one full trifluoromethansulfonate in part -1 with an occupancy of 0.5 and extending it into the other asymmetric residue unit. The other trifluoromethanesulfonate is also disordered with almost equal contributions 53% vs 47%. Several more orientations of this counterion are possible, and this simplified description is the main source for the high R values of the data set. In the main residue, no restraints were necessary except for a DFIX restraint for the stable refinement of the hydroxy-ligand hydrogen atom. One water, two acetonitrile and one diethyl ether solvent molecules as well as three trifluoromethanesulfonate counterions were found per **Cr_2-An** complex in accordance with a assignment of a Cr(III) oxidation state.

All relevant bond lengths and angles in the primary coordination sphere of the chromium atoms were determined from the crystal structures and are summarized in Table S1, along with the values found in structurally analogous literature-known chromium(III) dimers. The Cr–Cr distance is found to be ~2.95 Å for both complexes, suggesting the absence of metal-metal bond.[28] Due to a two-fold rotational symmetry in **Cr_2-An**, both chromium atoms are equivalent and have only one set of bond lengths and angles. **Cr_2-An** and **Cr_2-ref** are structurally very similar, with their equivalent bond distances being within 0.03 Å and bond angles within 3° of each other. Both complexes show a bridging geometry that is in accordance with the required structural parameters to obtain ferromagnetically coupled chromium(III) dimers.[29] Furthermore, both complexes exhibit a pseudo-octahedral coordination of the chromium(III) ions with most bite angles close to 90°, which was found to be a key factor to achieve room-temperature luminescence for chromium(III) dimers.[30] The pseudo-octahedral coordination is enabled by the dqp ligand's ability to bind in a facial mode, which is required for the present bridging geometry. The biggest difference between the two compounds is found in the distance between the central pyridine rings of the polypyridine ligands (Figure S1 and Figure S2). The pyridine rings in **Cr_2-ref** are aligned near parallel to each other with C-C distances of 3.69 Å – 3.81 Å, presumably due to π-π interactions.[31] In **Cr_2-An**, the two pyridine rings have a clear fold angle, giving a distance increase to 3.87 Å – 4.74 Å. This fold angle is most likely induced by the two bulky anthracene moieties, which show an offset stacked alignment with a interplanar distance of 3.31 Å – 3.91 Å that falls in the typical range for π-π interactions (Figure S2, middle right).[31]

Table S1: Key bond lengths and angles (standard deviation in parentheses) in the primary coordination sphere of the chromium centers in **Cr_2-ref** and **Cr_2-An** (this work) as well as the literature known compounds $[Cr_2L_2(\mu\text{-}SR)_2(\mu\text{-}O_2C_2H_3)][ClO_4]_3$[32] and $[Cr_2(dNinp)_2(\mu\text{-}OH)_2(\mu\text{-}O_2C_2H_3)][OTf]_3$[23]. N_L = laterally coordinating N-atom, N_c = centrally coordinating N-atom, O_H = O-atom in the hydroxide bridge or S-atom in the thiolate bridge, O_C = O-atom in the acetate bridge.

	Cr_2-ref	Cr_2-An	$[Cr_2L_2(\mu\text{-}SR)_2(\mu\text{-}O_2C_2H_3)][ClO_4]_3$[32]	$[Cr_2(dNinp)_2(\mu\text{-}OH)_2(\mu\text{-}O_2C_2H_3)][OTf]_3$[23]
$N_L – Cr – N_L$ angle / °	99.0(2) / 99.1(2)	101.90(14)	99.31(8) / 99.88(8)	99.4(2) / 100.9(2)
$N_L – Cr – N_C$ angle / °	87.0(2) / 87.1(2) / 87.1(2) / 87.0(2)	87.13(13) / 87.15(14)	83.52(8) / 82.27(9) / 82.59(8) / 83.67(10)	86.72(19) / 85.9(2) / 86.5(2) / 86.2(2)
$N_L – Cr – O_H$ (cis) angle / °	90.5(2) / 91.4(2) / 90.6(2) / 91.3(2)	88.62(13) / 90.39(13)	92.55(6) / 91.10(6) / 91.98(6) / 90.90(6)	91.48(19) / 89.87(19) / 91.04(19) / 88.8(2)
$N_L – Cr – O_H$ (trans) angle / °	169.2(2) / 170.4(2) / 169.3(2) / 170.2(2)	169.40(12) / 167.02(14)	169.58(6) / 167.17(6) / 169.20(6) / 167.85(6)	169.3(2) / 168.26(19) / 170.27(19) / 167.7(2)
$N_L – Cr – O_C$ angle / °	85.0(7) / 96.4(8) / 86.8(8) / 95.7(7)	91.55(13) / 88.67(14)	89.29(8) / 91.29(8) / 88.99(8) / 90.56(9)	93.7(2) / 91.5(2) / 89.8(2) / 90.1(2)
$N_C – Cr – O_H$ angle / °	90.4(2) / 92.3(2) / 90.7(2) / 91.7(2)	94.72(13) / 89.37(12)	94.29(7) / 98.34(6) / 97.93(6) / 95.33(7)	90.32(18) / 88.71(18) / 91.40(18) / 93.14(19)
$N_C – Cr – O_C$ angle / °	171.7(7) / 173.6(7)	175.25(14)	169.43(8) / 168.81(8)	177.4(2) / 174.19(18)
$O_H – Cr – O_H$ angle / °	79.0(2) / 79.0(2)	79.21(14)	77.11(3) / 77.22(2)	78.91(17) / 79.24(16)
$O_H – Cr – O_C$ angle / °	87.5(9) / 95.1(8) / 95.0(8) / 86.5(8)	89.4(2) / 92.7(2)	93.76(6) / 90.13(6) / 91.73(6) / 92.31(6)	92.23(18) / 91.33(18) / 93.35(18) / 91.03(18)
$Cr – O_H – Cr$ angle / °	98.7(2) / 98.7(2)	98.22(13)	93.53(3) / 92.12(3)	97.81(18) / 97.90(18)
Cr – Cr distance / Å	2.9510(17)	2.9496(13)	3.5449(10)	2.9326(13)
$Cr – N_L$ length / Å	2.091(6) / 2.088(6) / 2.088(6) / 2.088(6)	2.068(3) / 2.080(4)	2.168(2) / 2.206(2) 2.203(2) / 2.180(2)	2.049(5) / 2.055(5) / 2.061(5) / 2.070(5)
$Cr – N_C$ length / Å	2.032(5) / 2.045(5)	2.047(3)	2.120(2) / 2.119(2)	2.089(5) / 2.107(5)
$Cr – O_H$ length / Å	1.941(5) / 1.946(5) / 1.948(5) / 1.943(5)	1.946(3) / 1.956(3)	2.4302(9) / 2.4671(8) / 2.4356(8) / 2.4560(9)	1.948(4) / 1.949(4) / 1.944(4) / 1.940(4)
$Cr – O_C$ length / Å	2.001(12) / 1.998(11)	1.980(3)	1.9483(18) / 1.9534(18)	1.981(5) / 1.946(4)

Table S2: Crystallographic data for **Cr_2-ref** and **Cr_2-An**.

Identification code / CCDC	Cr_2-ref / 2565408	Cr_2-An / 2565409
Empirical formula	$C_{54}H_{51}Cr_2F_9N_6O_{18}S_3$	$C_{103}H_{83}Cr_2F_9N_{10}O_{15}S_3$
Formula weight	1443.19	2071.97
Temperature/K	100	100(2)
Crystal system	triclinic	monoclinic
Space group	P-1	C2/c
a/Å	15.5530(7)	16.4038(6)
b/Å	15.8889(7)	40.8458(11)
c/Å	15.8900(8)	14.8784(5)
α/°	102.591(4)	90
β/°	112.801(3)	103.7370(10)
γ/°	114.217(3)	90
Volume/Å^3	2950.9(3)	9683.8(5)
Z	2	4
ρ_{calc}g/cm^3	1.624	1.421
μ/mm^{-1}	0.581	0.378
F(000)	1476	4272
Crystal size/mm^3	0.35 × 0.323 × 0.3	0.49 × 0.231 × 0.13
Radiation	Mo Kα (λ = 0.71073)	MoKα (λ = 0.71073)
2Θ range for data collection/°	4.774 to 50.5	3.452 to 54.278
Index ranges	-18 ≤ h ≤ 18, -18 ≤ k ≤ 19, -19 ≤ l ≤ 19	-21 ≤ h ≤ 21, -52 ≤ k ≤ 38, -19 ≤ l ≤ 19
Reflections collected	20098	42945
Independent reflections	10618 [R_{int} = 0.0378, R_{sigma} = 0.0451]	10702 [R_{int} = 0.0713, R_{sigma} = 0.0920]
Data/restraints/parameters	10618/254/1190	10702/289/733
Goodness-of-fit on F^2	1.538	1.04
Final R indexes [I>=2σ (I)]	R_1 = 0.1247, wR_2 = 0.3432	R_1 = 0.0874, wR_2 = 0.2255
Final R indexes [all data]	R_1 = 0.1545, wR_2 = 0.3747	R_1 = 0.1509, wR_2 = 0.2709
Largest diff. peak/hole / e Å^{-3}	3.94/-0.95	0.94/-0.56

4. Magnetic properties

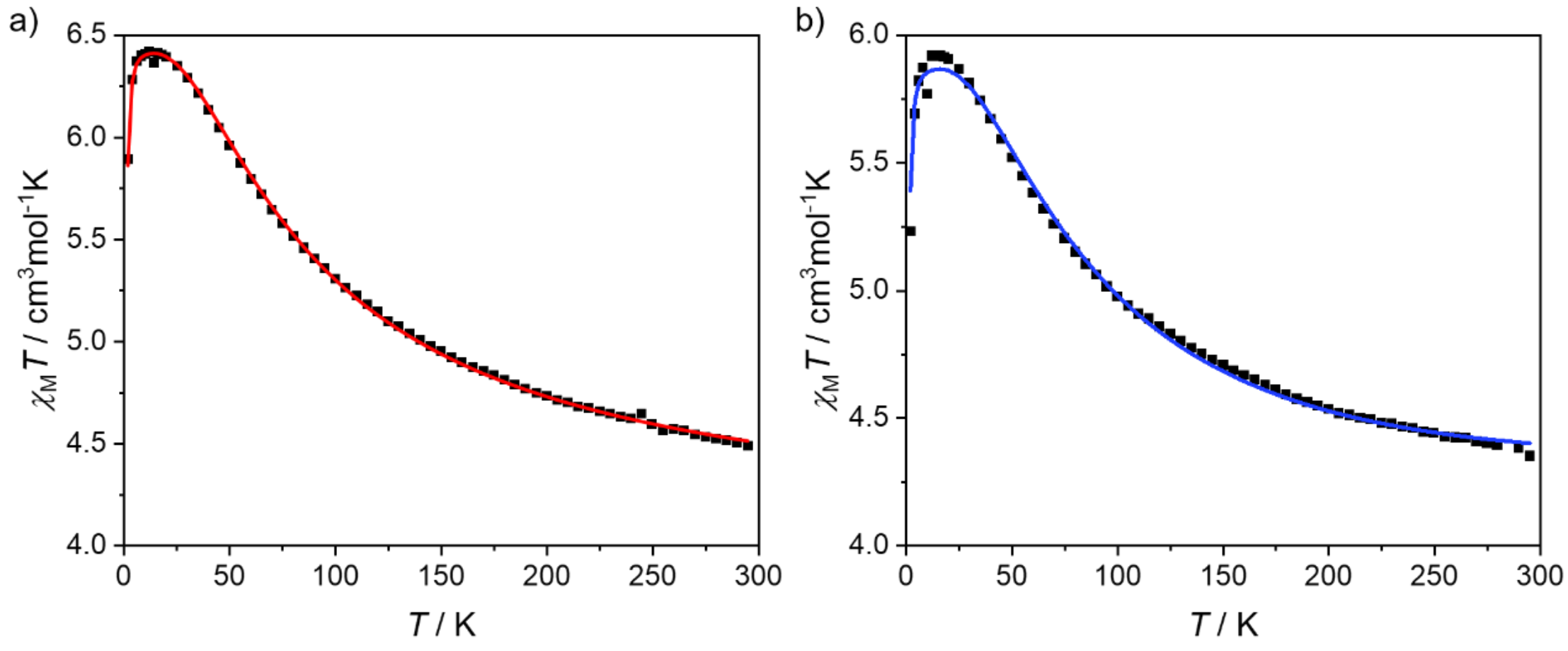

Figure S3: a) Temperature-dependent magnetic susceptibility data for **Cr_2-ref**. The red curve represents the best fit with parameters: J = + 10.5 cm^{-1} and g_1 = g_2 = 2.07. b) Temperature-dependent magnetic susceptibility data for **Cr_2-An**. The blue curve represents the best fit with parameters: J = + 11.2 cm^{-1} and g_1 = g_2 = 1.98.

Variable-temperature magnetic susceptibility measurements of **Cr_2-An** and **Cr_2-ref** were conducted to determine the magnetic exchange interactions of the complexes in their ground states. The susceptibility of powdered solid samples of the compounds was measured over a temperature range between 2 K and 295 K in an external magnetic field of 0.5 T. Figure S3 shows the temperature dependence of the $\chi_M T$ product, where χ_M is the molar magnetic susceptibility. Upon decrease of the temperature, the $\chi_M T$ product in **Cr_2-ref** increases from a value of 4.49 cm^{3}mol^{-1}K at 295 K to a maximum of 6.42 cm^{3}mol^{-1}K at 12 K, before dropping to a value of 5.89 cm^{3}mol^{-1}K at 2 K. A very similar trend can be observed in **Cr_2-An**, where the $\chi_M T$ product increases from 4.35 cm^{3}mol^{-1}K (295 K) to a maximum of 5.92 cm^{3}mol^{-1}K (14 K), before dropping to 5.23 cm^{3}mol^{-1}K (2 K). The $\chi_M T$ product of both dimers is higher than 3.75 cm^{3}mol^{-1}K, which is the spin-only value for two non-interacting ions with spin S = 3/2.[29, 33] These higher $\chi_M T$ values for **Cr_2-ref** and **Cr_2-An** are indicative of ferromagnetic exchange interactions between the chromium ions to give a high-spin ground state with S = 3. The experimental data was fitted to the spin Hamiltonian $\hat{H}$ (eq. S2), giving coupling constants of J = + 10.5 cm^{-1} (g_1 = g_2 = 2.07) for **Cr_2-ref** and J = + 11.2 cm^{-1} (g_1 = g_2 = 1.98) for **Cr_2-An**.

$$\hat{H} = -2J\hat{S}_1\hat{S}_2 + g\mu_B\vec{B}(\vec{S}_1 + \vec{S}_2) \qquad eq.(S2)$$

S_1 and S_2 represent the respective electron spins of the chromium ions, μ_B is the Bohr magneton, and B indicates the magnetic flux density. The ferromagnetic coupling in these dimers is in line with literature-known compounds with an equivalent bridging geometry between the chromium ions.[23, 29, 32] The coupling constants resemble those in the structurally most similar complex $[Cr_2(dNinp)_2(\mu\text{-}OH)_2(\mu\text{-}O_2C_2H_3)][OTf]_3$ (J = + 9.3 cm^{-1}).[23] The slightly longer Cr– Cr distance found in **Cr_2-ref** and **Cr_2-An** (Table S1) could account for their higher coupling constants, according to magneto-structural correlations.[29, 30] The coupling constant found in the thiolate-bridged dimer $[Cr_2L_2(\mu\text{-}SR)_2(\mu\text{-}O_2C_2H_3)][ClO_4]_3$ (J = + 24.2 cm^{-1})[32] is significantly larger, which is likely affected by the smaller (closer to 90°) Cr-S-Cr bond angles that result in a larger Cr–Cr distance and consequently weaker orbital overlap between the chromium centers (Table S1).[30]

5. Electrochemistry

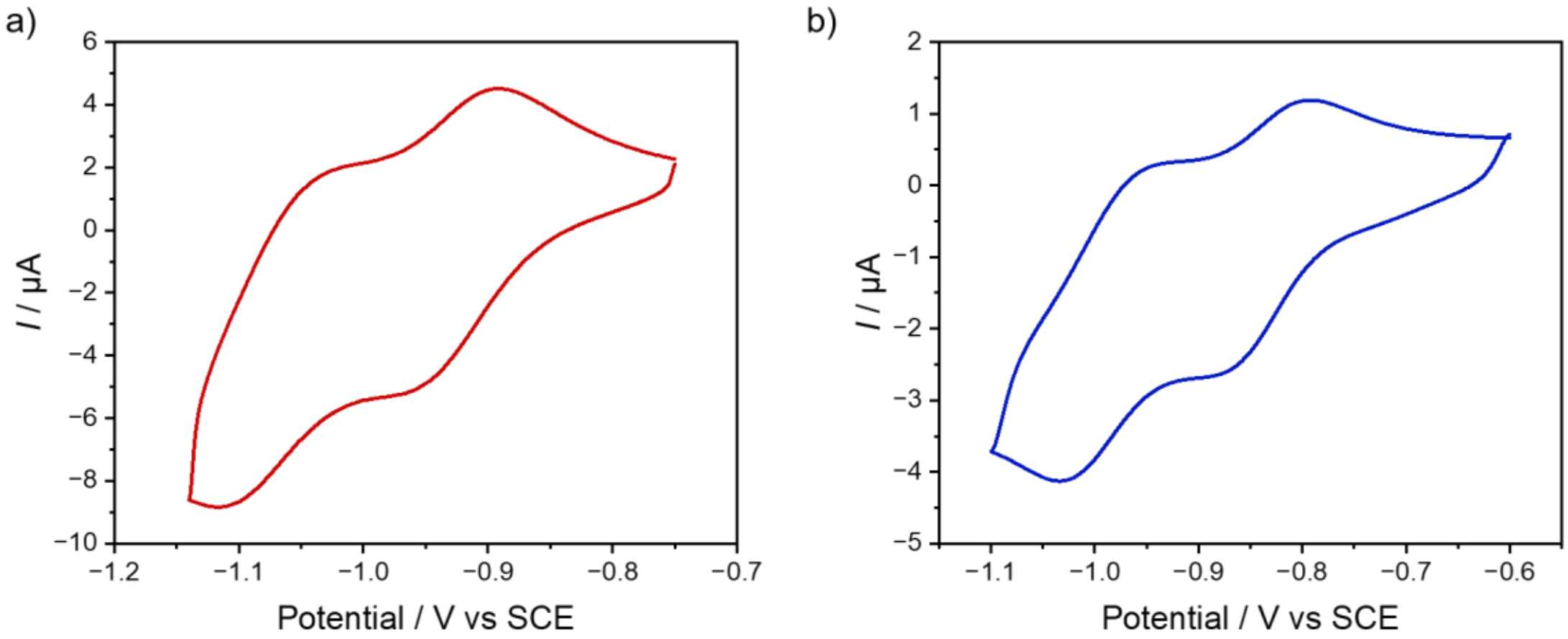

Figure S4: Cyclic voltammograms of a) **Cr_2-ref** and b) **Cr_2-An** in deaerated dry dimethylformamide (DMF). $TBAPF_6$ (0.1 M) was used as the supporting electrolyte. A glassy carbon disk, a platinum wire and a saturated calomel electrode were used as working electrode, counter electrode and reference electrode respectively. The potential scan rate was 0.1 V/s.

6. Photophysical characterization

6.1 Cr_2-ref

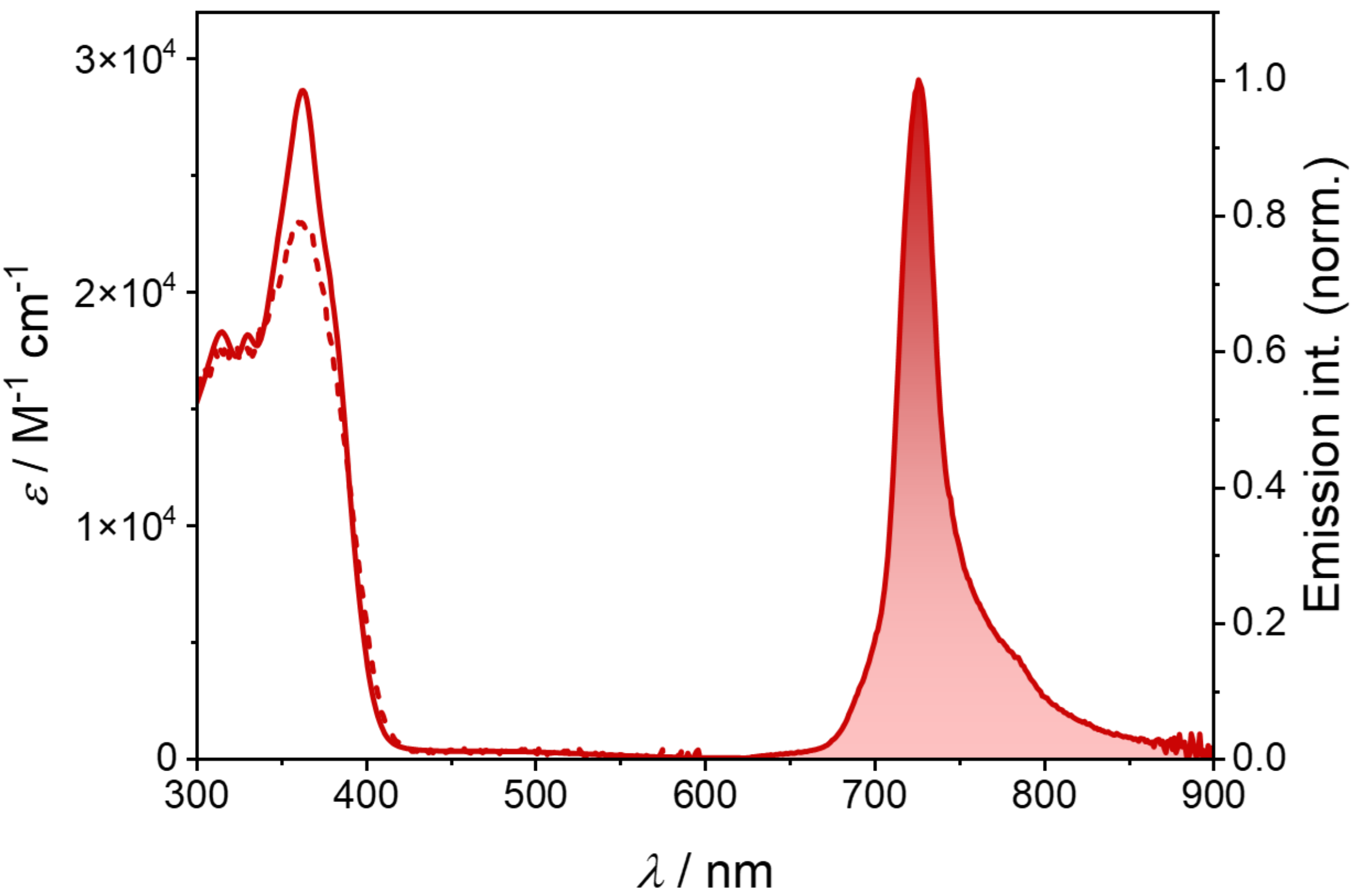

Figure S5: UV-Vis absorption spectrum (solid red trace), normalized luminescence spectrum (red area, excitation at 375 nm) and excitation spectrum (dashed red trace, emission detected at 726 nm) of **Cr_2-ref**. The spectra were measured at a concentration of 50 μM (absorption, emission) or 15 μM (excitation) in deaerated acetonitrile at 20 °C.

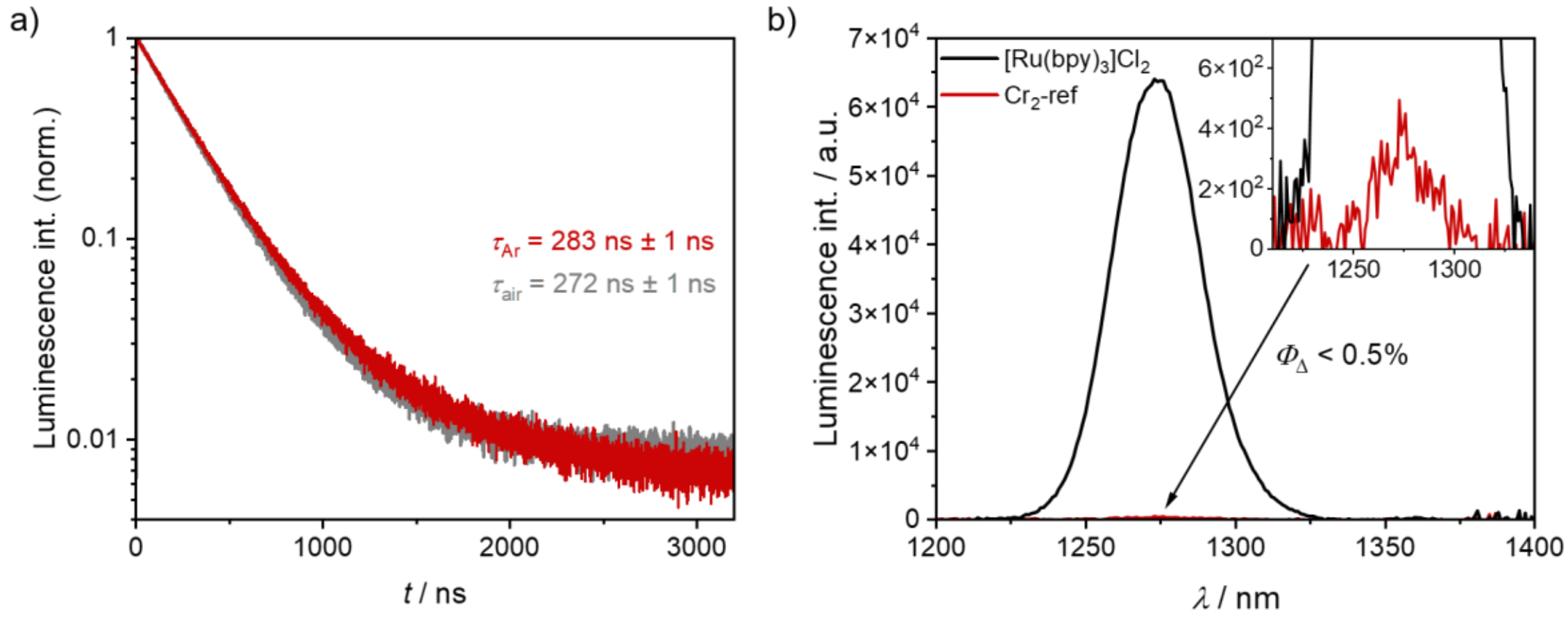

Figure S6: a) Luminescence lifetime measurements of **Cr$_2$-ref** (50 µM) in deaerated (red trace) and aerated (gray trace) acetonitrile at 20 °C. The samples were excited at 375 nm and the luminescence was detected at 726 nm. b) Luminescence spectra of $^1O_2$ sensitized by **Cr$_2$-ref** (200 µM, red trace) and [Ru(bpy)$_3$]Cl$_2$ (17 µM, black trace) upon excitation at 485 nm in air-equilibrated acetonitrile at 20 °C. The sample concentrations were adjusted to being isoabsorbing at the excitation wavelength. The $^1O_2$ quantum yield (Φ_Δ) of **Cr$_2$-ref** was determined relative to the known value for the reference [Ru(bpy)$_3$]Cl$_2$ (Φ_Δ = 57%).[34]

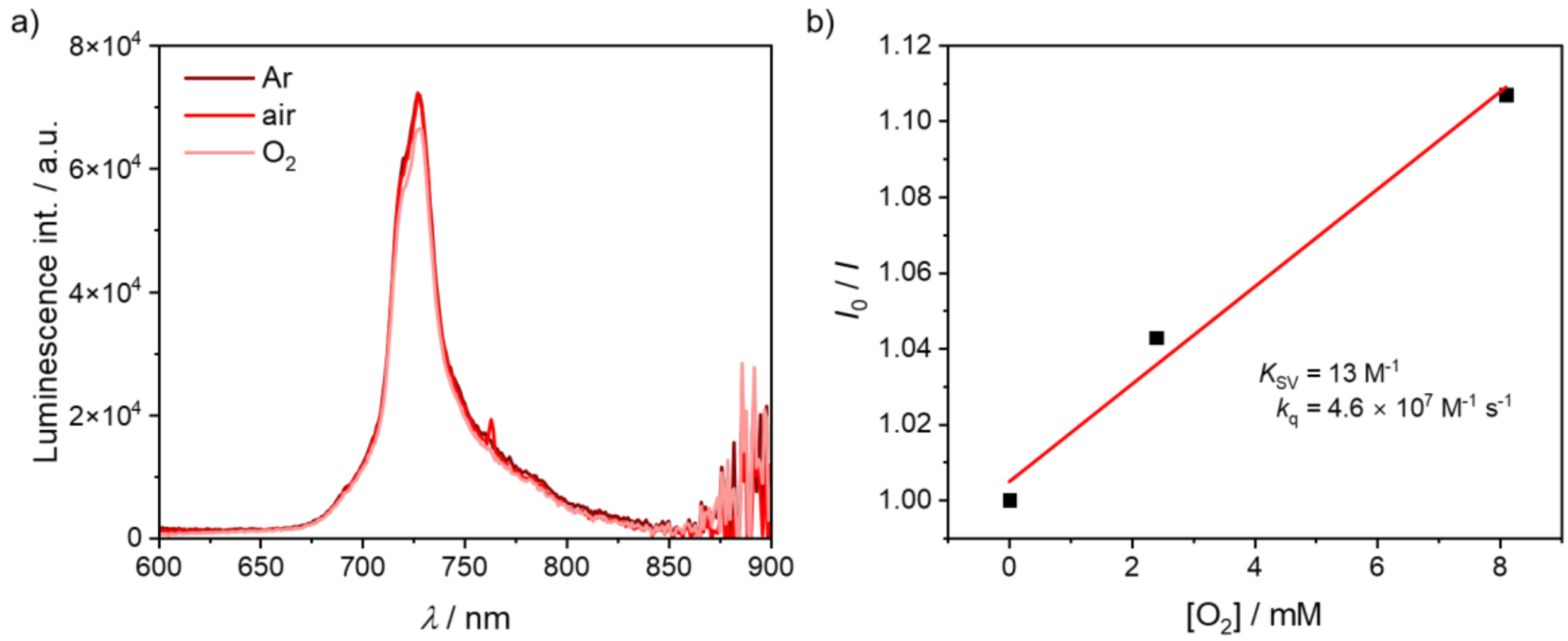

Figure S7: a) Luminescence spectra of **Cr$_2$-ref** (50 µM in acetonitrile) at 20 °C upon excitation at 375 nm under deaerated (dark red trace), aerated and O_2 saturated (light red trace) conditions. b) Corresponding Stern-Volmer plot of the luminescence intensities from a) (integrated from 650-850 nm). The oxygen concentrations for air-equilibrated and O_2-saturated conditions were found in the literature.[35, 36] A linear fit gives the Stern-Volmer constant K_{SV} from the slope. The quenching rate constant k_q can be calculated from $k_q = K_{SV} / \tau_0$ (τ_0 = 283 ns from Figure S6).

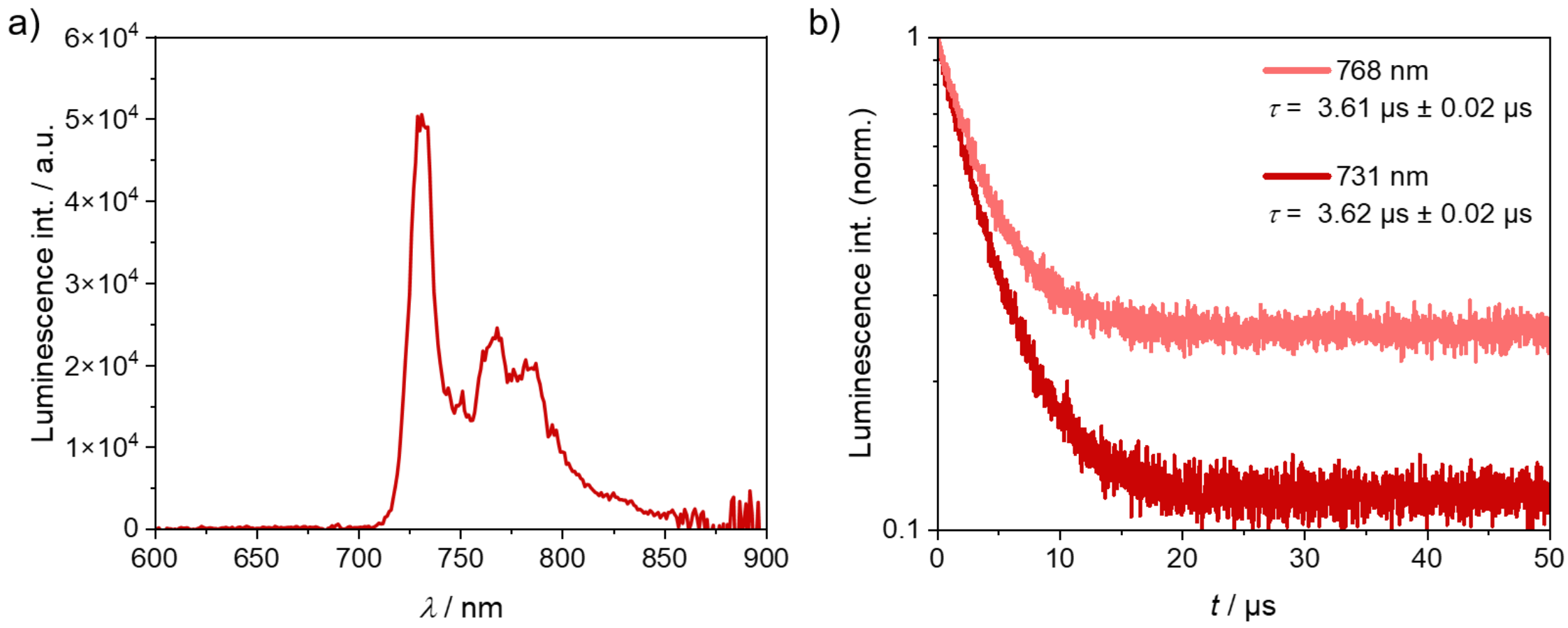

Figure S8: a) Luminescence spectrum of **Cr_2-ref** (50 μM in ethanol) measured at 77 K upon excitation at 375 nm. b) Luminescence lifetime measurements of **Cr_2-ref** (50 μM in ethanol). The sample was excited at 375 nm and the luminescence was detected at 731 nm (dark-red trace) and 768 nm (light-red trace).

UV-Vis absorption measurements of **Cr_2-ref** in acetonitrile at 293 K show a weak absorption band between 400 nm and 550 nm ($\varepsilon_{490} \approx 340$ M^{-1} cm^{-1}) from the metal-centered transition and a strong absorption band in the UV range ($\varepsilon_{363} \approx 2.9 \times 10^4$ M^{-1} cm^{-1}) arising from the $^4A_2 \rightarrow ^4$LMCT transition. Excitation into either band at room temperature yields a narrow emission band centered at 726 nm, which is similar to literature-known chromium(III) monomers and dimers and corresponds to a spin-flip emission from the $^2T_1{}^4A_2/^2E^4A_2$ states.[23, 32, 37-39] The excitation spectrum of **Cr_2-ref** monitored at 726 nm reproduces the aforementioned transitions in the absorption spectrum. The luminescence decays with a lifetime of 283 ns and shows only very minor sensitivity towards quenching by oxygen. This was confirmed by the determination of the $^1O_2$ quantum yield (Φ_Δ), which was found to be below 0.5% (Figure S6). Luminescence intensity based quenching studies with dissolved molecular oxygen also showed minimal quenching with a rate constant of $k_q = 4.6 \times 10^7$ M^{-1} (Figure S7). At 77 K, the luminescence shows a vibrational substructure, which shows an emission wavelength-independent lifetime of 3.6 μs (Figure S8). The photoluminescence quantum yield of **Cr_2-ref** is estimated to be below $\Phi < 0.01\%$, making the quantitative determination challenging.

6.2 Cr_2-An

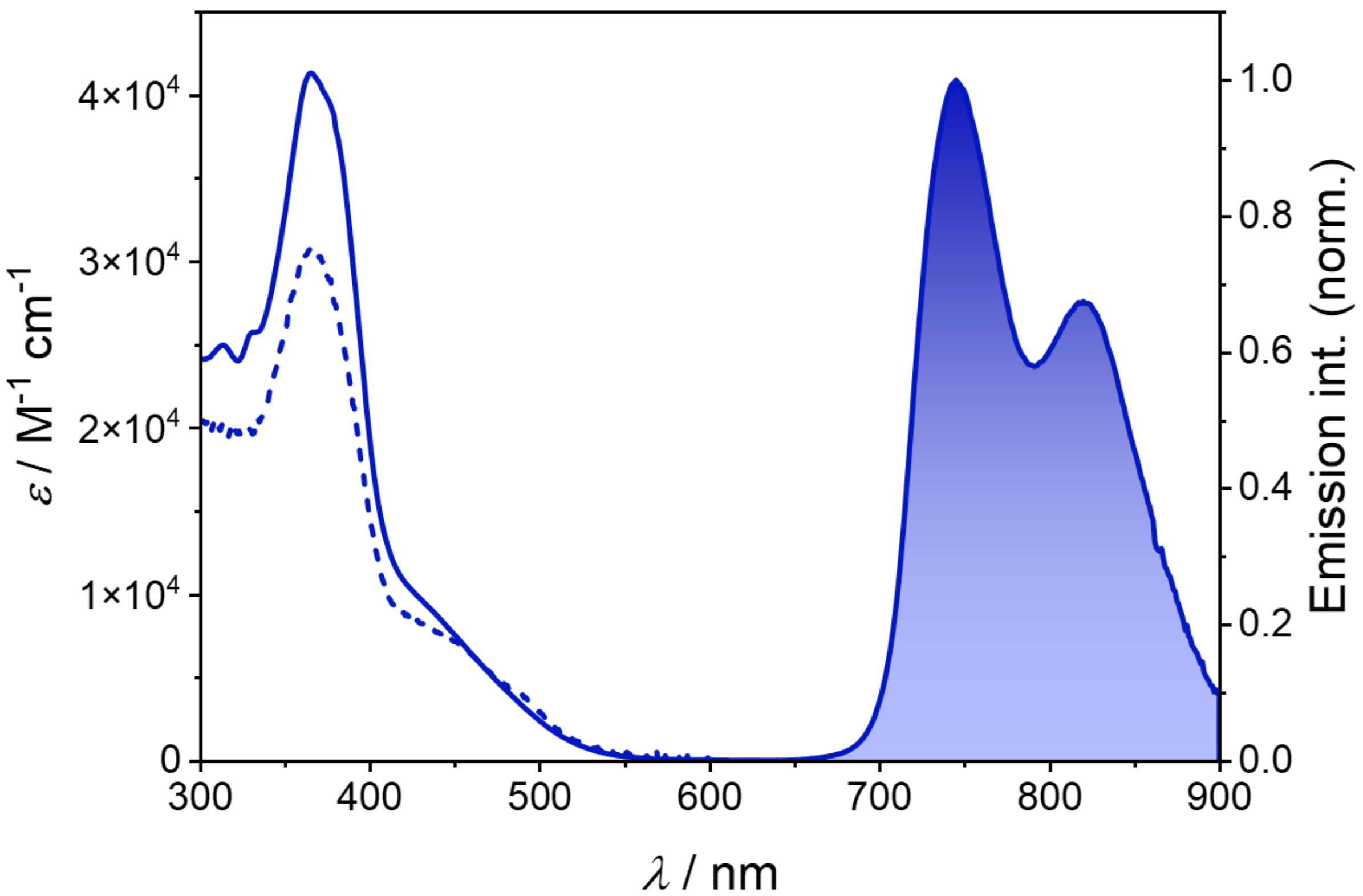

Figure S9: UV-Vis absorption spectrum (solid blue trace), normalized luminescence spectrum (blue area, excitation at 450 nm) and excitation spectrum (dashed blue trace, emission detected at 745 nm) of **Cr_2-An**. The spectra were measured at a concentration of 50 μM (absorption, emission) or 5 μM (excitation) in deaerated acetonitrile at 20 °C.

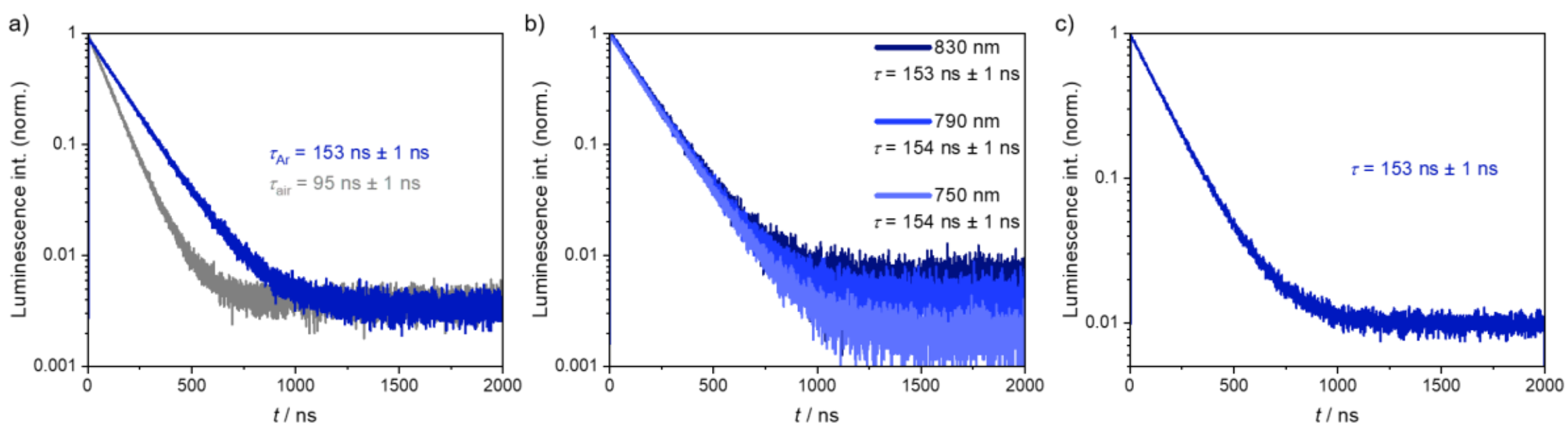

Figure S10: a) Luminescence lifetime measurements of **Cr_2-An** (50 μM) in deaerated (blue trace) and aerated (gray trace) acetonitrile at 20 °C. The samples were excited at 450 nm and the luminescence was detected at 745 nm. b) Luminescence lifetime measurements of **Cr_2-An** (50 μM) in deaerated acetonitrile at 20 °C. The sample was excited at 450 nm and the luminescence was detected at 750 nm (light-blue trace), 790 nm, and 830 nm (dark-blue trace). c) Luminescence lifetime measurements of **Cr_2-An** (50 μM) at 745 nm in deaerated ethanol at 20 °C upon excitation at 450 nm.

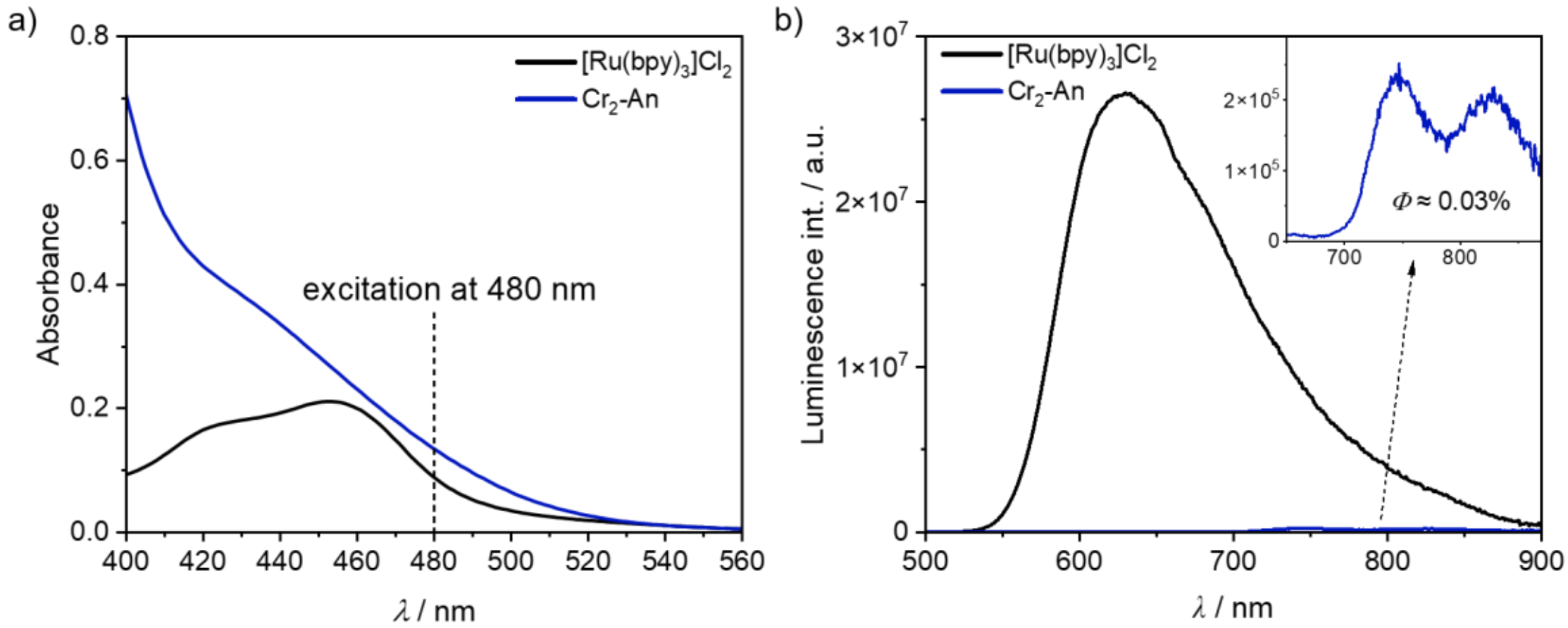

Figure S11: a) UV-Vis absorption spectra of $[Ru(bpy)_3]Cl_2$ (20 µM in deaerated water, black trace) and **Cr_2-An** (40 µM in deaerated acetonitrile, blue trace) at 20 °C. b) Luminescence spectra of $[Ru(bpy)_3]Cl_2$ (Φ_{PL} = 6.3%)[7] and **Cr_2-An** (samples from a)) upon excitation at 480 nm. The emission of **Cr_2-An** is shown enlarged in the inset. The photoluminescence quantum yield was determined to be ~ 0.03% according to equation S1.

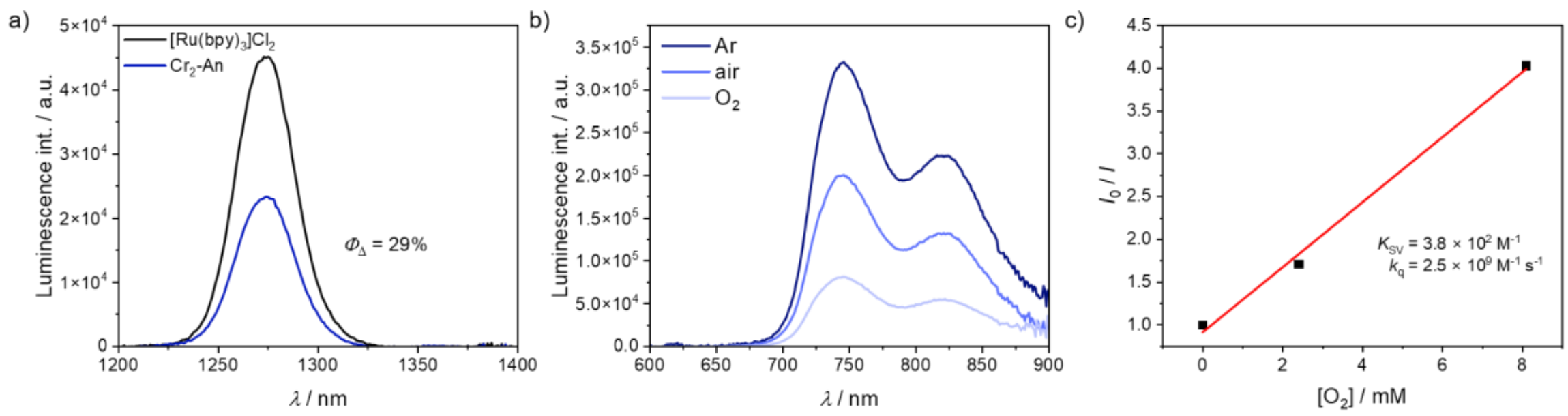

Figure S12: a) Luminescence spectra of $^1O_2$ sensitized by **Cr_2-An** (50 µM, blue trace) and $[Ru(bpy)_3]Cl_2$ (48 µM, black trace) upon excitation at 485 nm in air-equilibrated acetonitrile at 20 °C. The sample concentrations were adjusted to being isoabsorbing at the excitation wavelength. The $^1O_2$ quantum yield (Φ_Δ) of **Cr_2-An** was determined relative to the known value for the reference $[Ru(bpy)_3]Cl_2$ (Φ_Δ = 57%).[34] b) Luminescence spectra of **Cr_2-An** (50 µM in acetonitrile) at 20 °C upon excitation at 375 nm under deaerated (dark blue trace), aerated and O_2 saturated (light blue trace) conditions. c) Corresponding Stern-Volmer plot of the luminescence intensities from b) (integrated from 650-900 nm). The oxygen concentrations for air-equilibrated and O_2-saturated conditions were found in the literature.[35, 36] A linear fit gives the Stern-Volmer constant K_{SV} from the slope. The quenching rate constant k_q can be calculated from $k_q = K_{SV} / \tau_0$ (τ_0 = 153 ns from Figure S10).

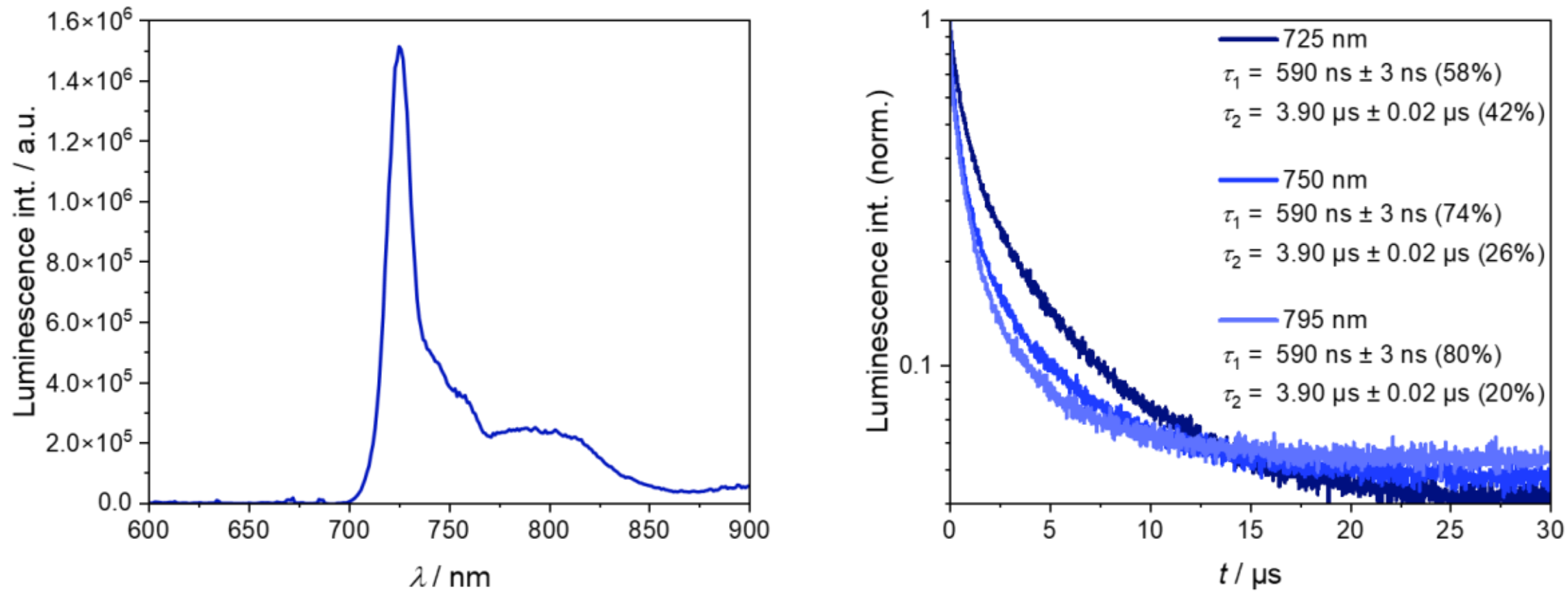

Figure S13: a) Luminescence spectrum of **Cr_2-An** (50 μM in ethanol) measured at 77 K upon excitation at 450 nm. b) Luminescence lifetime measurements of **Cr_2-An** (50 μM in ethanol) at 77 K at different wavelengths upon excitation at 450 nm. Initial bi-exponential fit of these decays give two stable time constants. By fixing these time constants to 3.9 μs and 590 ns, the bi-exponential fitting results show distinct relative amplitudes (in parentheses).

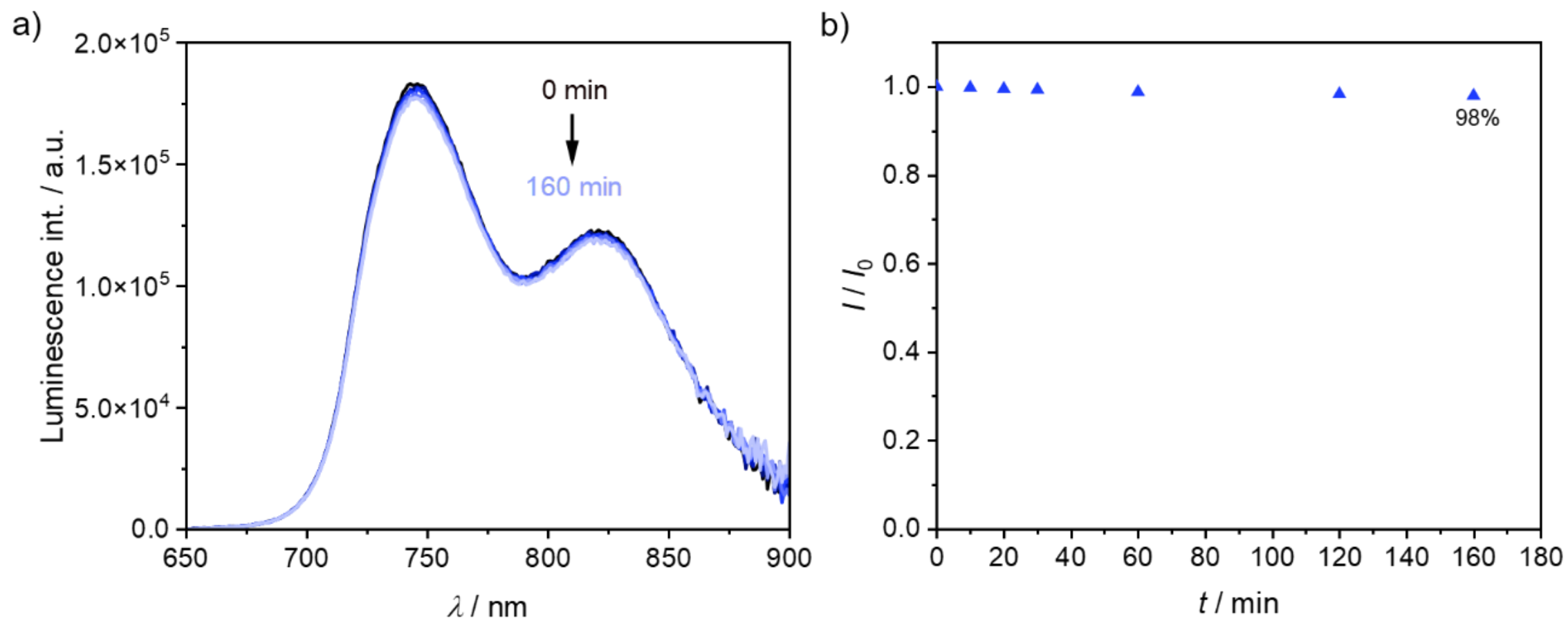

Figure S14: a) Luminescence spectra of **Cr_2-An** (50 μM) in deaerated acetonitrile at 20 °C upon CW-laser (30.2 W cm^{-2}) excitation at 520 nm, measured at multiple time points (0 – 160 min) after continuous laser irradiation at 520 nm between measurements. b) Integrated luminescence intensity ($I_{650\text{-}900}$) from a) in relation to the initial luminescence intensity at time zero I_0, plotted as a function of the irradiation time. The luminescence intensity remains 98% of the initial intensity after 160 min of continuous laser irradiation.

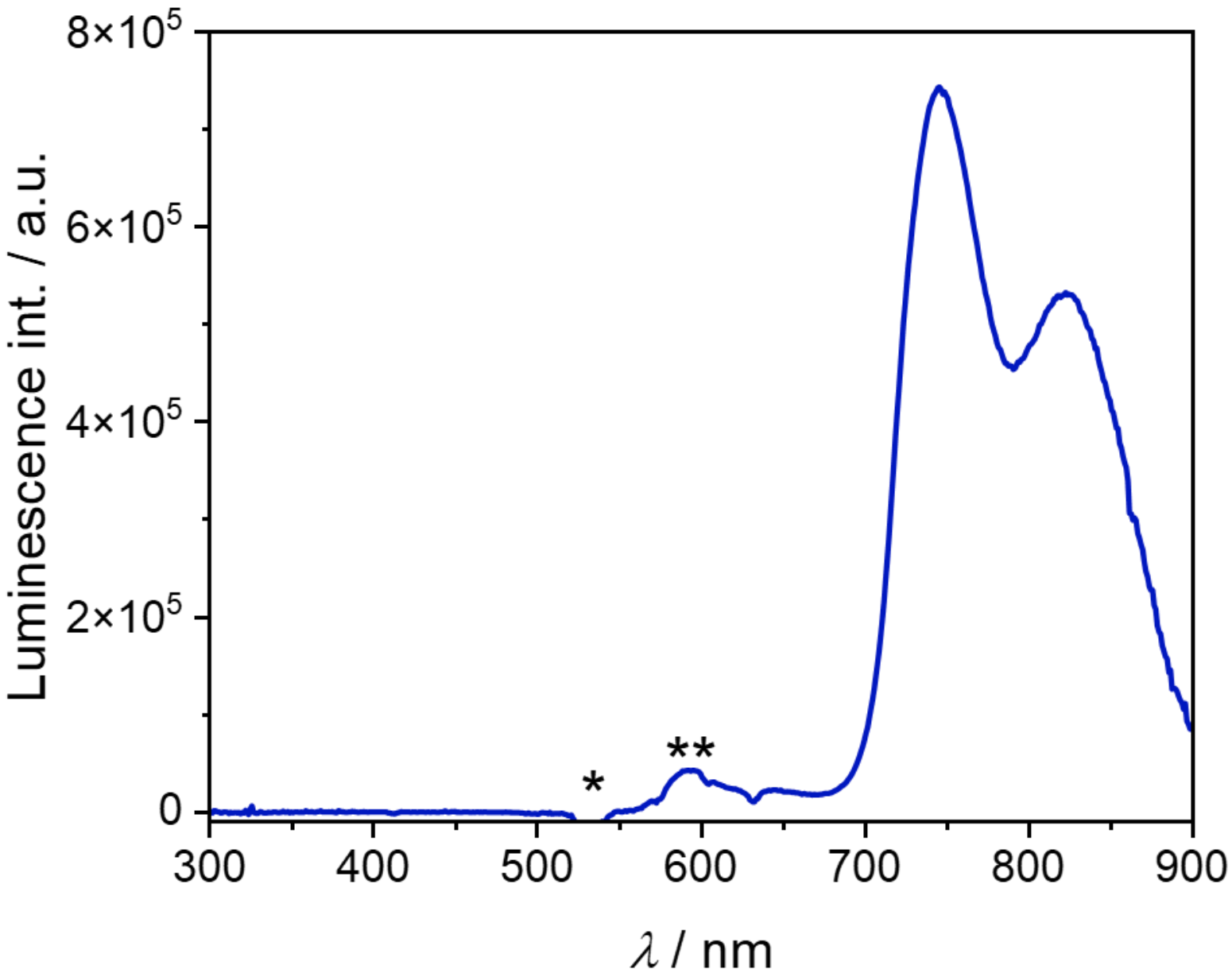

Figure S15: Luminescence spectrum of **Cr_2-An** (50 µM) in deaerated acetonitrile at 20 °C upon 532 nm CW-laser excitation (144 W cm^{-2}) measured in the full visible spectral regime. The signal marked with * denominates the scattered excitation light, which was cut from the measurement. The signal marked with ** is caused by instrument artefacts in that spectral region.

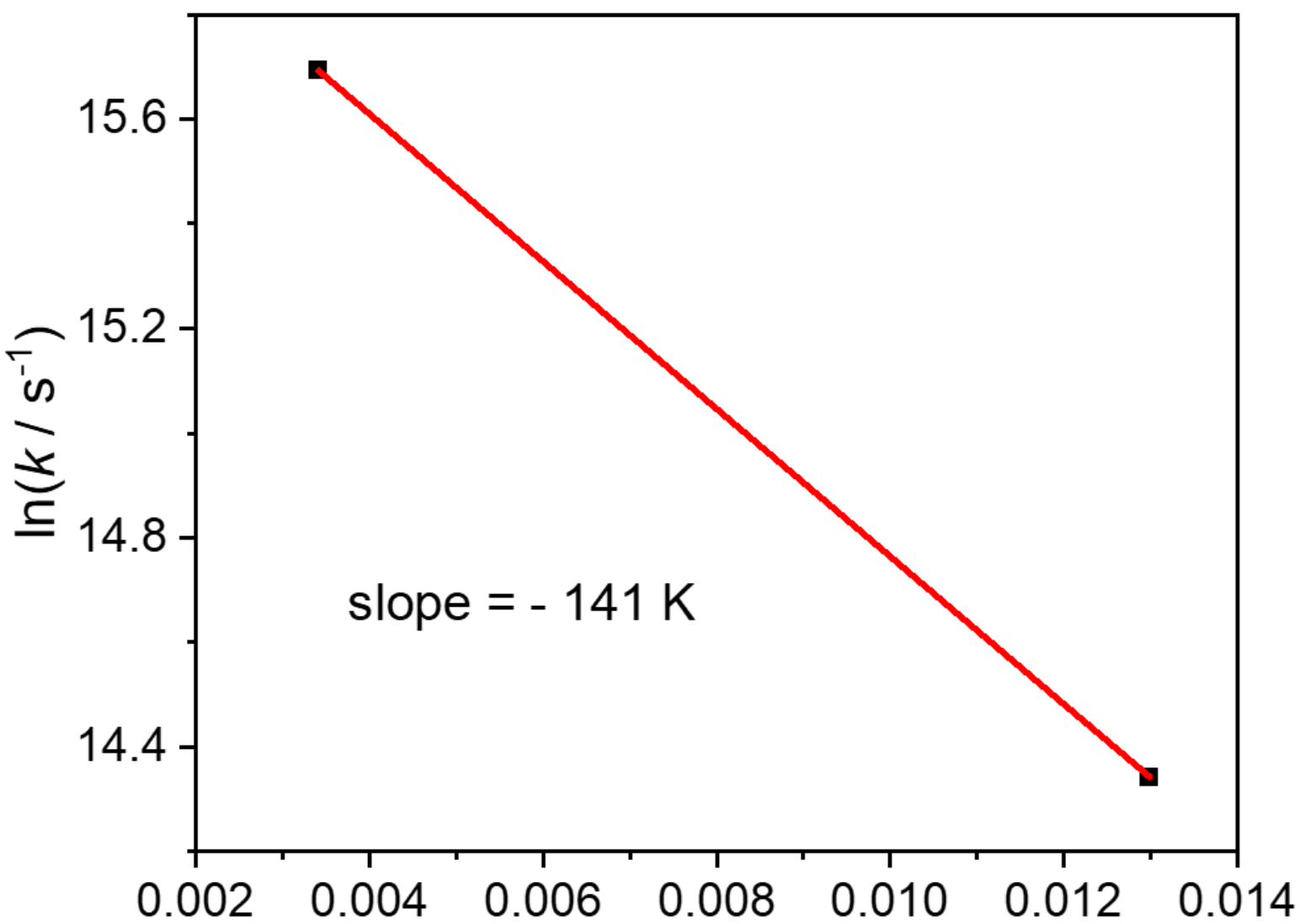

Figure S16: Arrhenius plot for the decay of the ^{3}PA* state of **Cr_2-An** based on the luminescence lifetimes in ethanol at room temperature (Figure S10c) and at 77 K (Figure S13b), with the rate constant $k = 1/\tau$.

UV-Vis absorption measurements of **Cr_2-An** show strong absorption between 400 nm and 550 nm ($\varepsilon_{440} \approx 8.7\times10^3$ M^{-1} cm^{-1}) and very strong absorption in the UV range ($\varepsilon_{366} \approx 4.1\times10^4$ M^{-1} cm^{-1}) (Figure S9a). Excitation into either band at room temperature yields broad double emission bands at 745/820 nm (Figure S9b). The excitation spectrum shows a good representation of the absorption spectrum, particularly in the visible range. The emission band resembles the shape of anthracene-based phosphorescence.[40, 41] The luminescence decays with a lifetime of 153 ns in deaerated solution at room temperature, independent of the detection wavelength (Figure S9). The luminescence lifetime also shows significantly higher oxygen sensitivity compared to that of **Cr_2-ref**. This oxygen sensitivity of **Cr_2-An** is further evidenced by the $^1O_2$ quantum yield determination, which yields a value of Φ_Δ = 29%, and intensity based quenching by molecular oxygen revealing a quenching rate constant of $k_q = 2.5 \times 10^9$ M^{-1} (Figure S12). As triplet states of organic chromophores have notably higher rate constants for quenching by oxygen compared to the quenching rate constants for metal-centered spin-flip states, [38, 39, 42, 43] this is further indication of the anthracene-based nature of the observed emission.

Surprisingly, the emission of **Cr_2-An** at 77 K differs strongly from the room-temperature luminescence. A narrow emission band centered at 725 nm that resembles the emission seen in **Cr_2-ref** can be observed, with weak broad bands at 750/795 nm as shoulders (Figure S13a). Luminescence decays at these wavelengths follow biexponential fit. This yields a short component of 590 ns, which is the main contribution at 750/795 nm, and a long component of 3.9 µs which is strongest at 725 nm (Figure S13b). Due to the spectral position and lifetime of the narrow band, both of which are very similar to **Cr_2-ref** at 77 K, it is assumed that the emission maximized at 725 nm stems from the $^2T_1{}^4A_2/{}^2E^4A_2$ states. The shorter-lived component with a lifetime of 590 ns arises likely from the triplet phenylanthracene excited state (^{3}PA*), which seems to exist in an equilibrium with the spin-flip excited states, likely due to the intramolecular doublet-triplet energy transfer reservoir. Although luminescence measurements at tunable temperatures could not be performed on our spectrometer, the activation energy barrier E_a can be estimated from an Arrhenius plot (Figure S16) based on the luminescence lifetime response of **Cr_2-An** in ethanol at room temperature (Figure S10c) and at 77 K (Figure S16). Based on the Arrhenius equation $\ln(k/\text{s-1}) = -E_a/RT + \ln(A)$, where k (= $1/\tau$) is the rate constant of the ^{3}PA* state, R is the universal gas constant, T is the temperature and A is a pre-exponential factor, the Arrhenius plot gives a slope corresponding to $-E_a/R$ = -141 K. This equates to an activation energy barrier E_a of 1.2 kJ/mol (~0.01 eV), which allows both the forward and reverse DTET process.

Homomolecular photon upconversion has been recently reported in iron(III)-perylene complexes via an unusual doublet-triplet annihilation. [44, 45] Excitation of **Cr_2-An** with a 532 nm laser (144 W cm^{-2}) only yields the broad anthracene-based phosphorescence, and no anthracene-based fluorescence was observed (Figure S15). This suggests that homomolecular photon upconversion does not occur in our compound, at least not under our conditions.

7. Density Functional Theory calculations

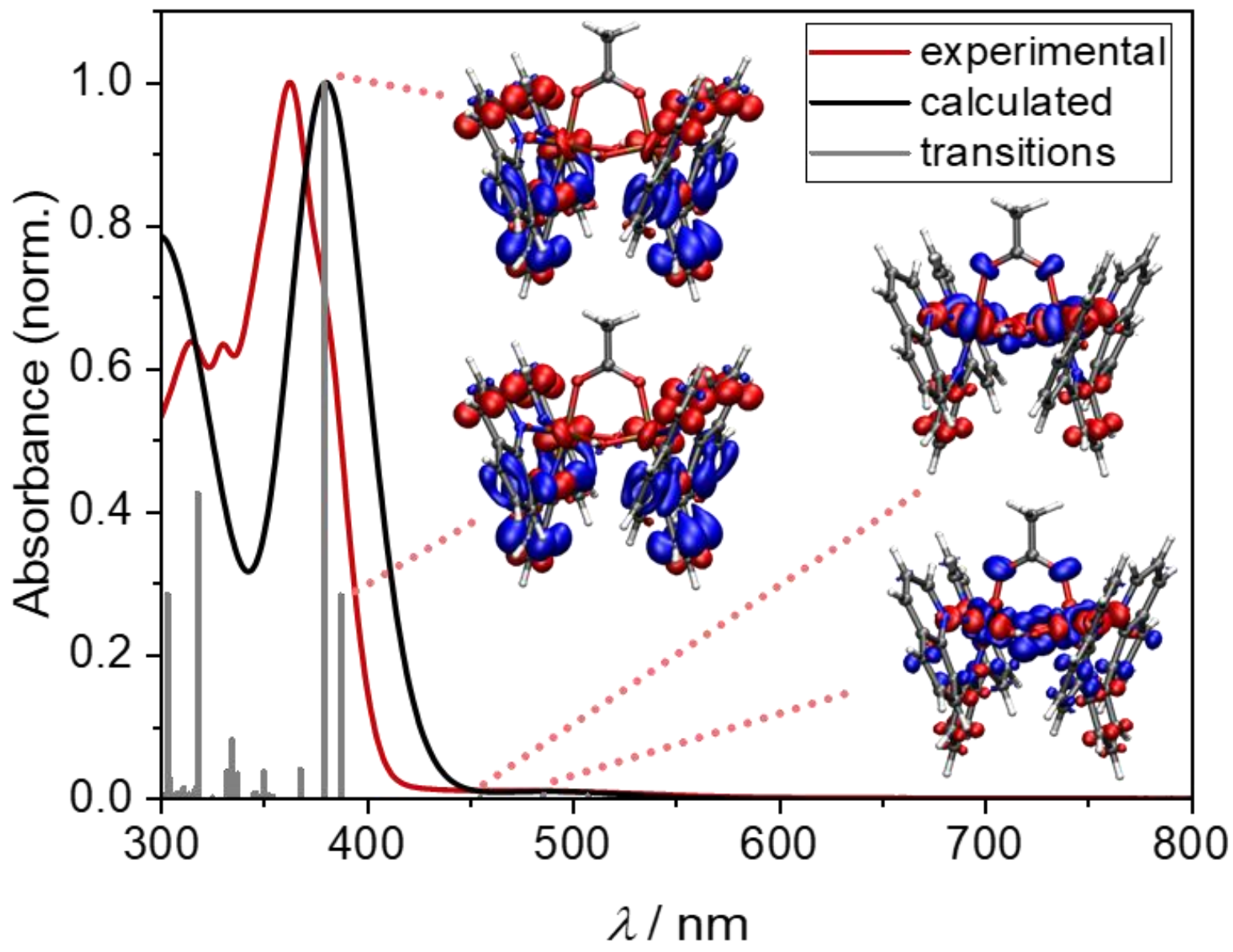

Figure S17: Experimental (red) and calculated (black) absorption spectra of **Cr_2-ref** in acetonitrile. Gray vertical lines represent the calculated transitions in the complex. Electron density difference maps (EDDM) of the relevant transitions are shown with blue color representing electron density loss and red color representing electron density gain.

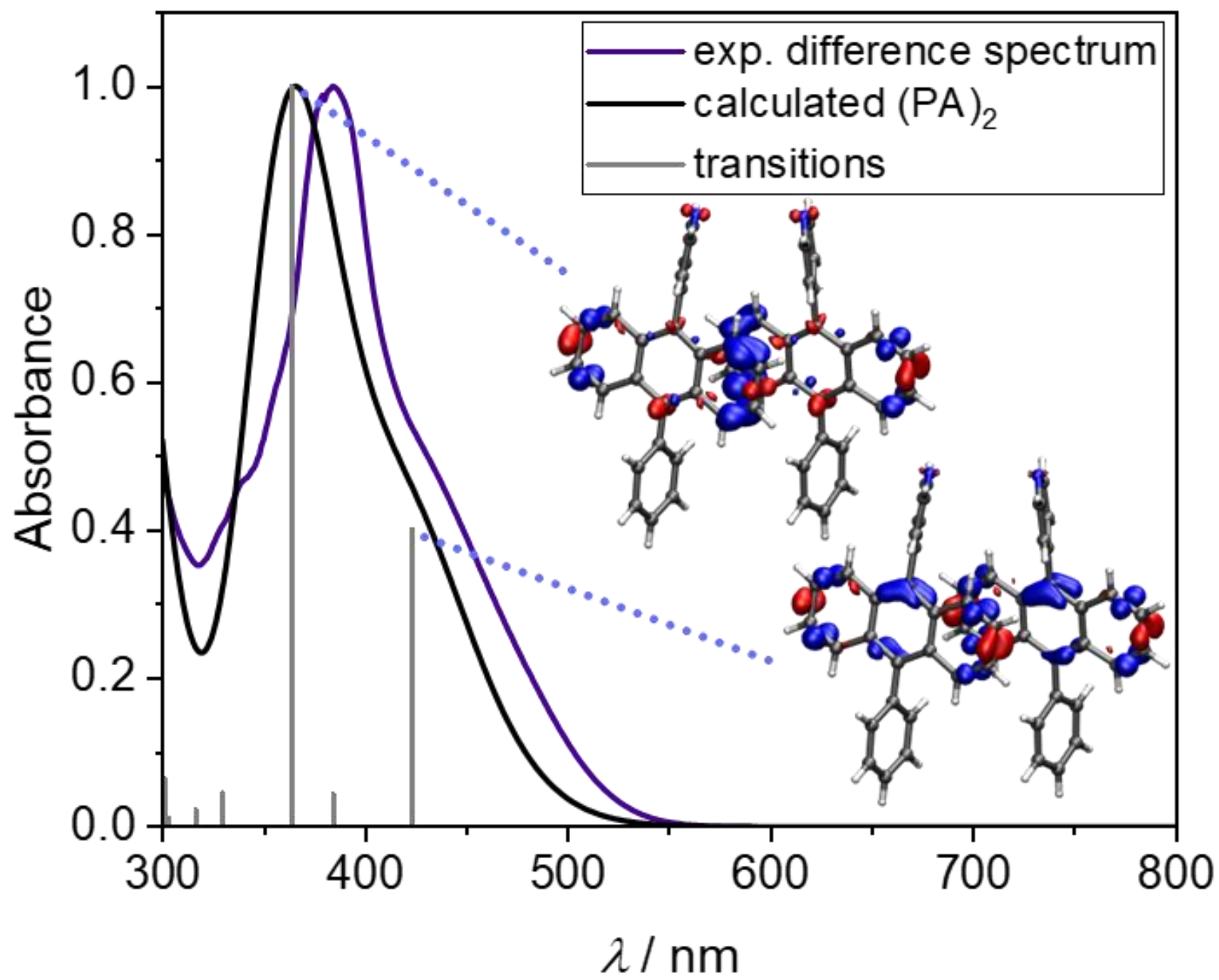

Figure S18: Experimental differential absorption spectrum (blue, **Cr_2-An** - **Cr_2-ref**) and calculated absorption spectrum of the associated phenlyanthracene fragment $(PA)_2$ (black). Gray vertical lines represent the calculated transitions. EDDMs of the two strongest transitions are shown with blue color representing electron density loss and red color representing electron density gain.

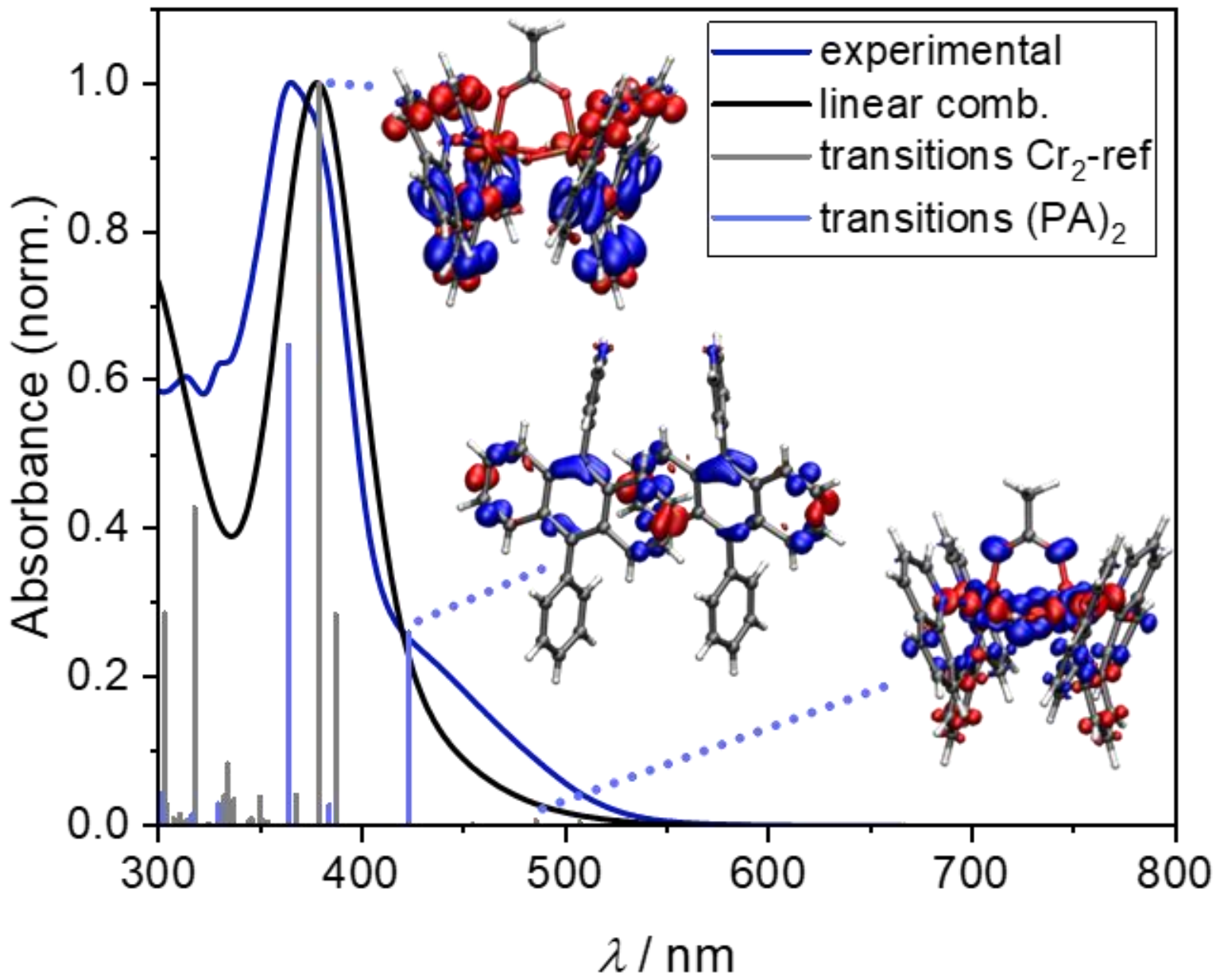

Figure S19: Experimental absorption spectrum of **Cr_2-An** in acetonitrile (dark blue) with the linear combination of the calculated absorption spectra of **Cr_2-ref** and $(PA)_2$ (black). Vertical lines represent the calculated transitions in **Cr_2-ref** (gray) and $(PA)_2$ (light blue). Electron density difference maps (EDDM) of relevant transitions are shown with blue color representing electron density loss and red color representing electron density gain.

Table S3: Atomic coordinates of **Cr_2-ref** for TD-DFT calculations.

Cr	-0.00714	1.53584	-0.59860
Cr	0.00798	-1.53585	-0.59841
O	-0.01296	1.13410	-2.55881
C	-0.00721	-0.00022	-3.17837
C	-0.05179	-0.00079	-4.67324
H	0.41758	0.89520	-5.06957
H	-1.09510	-0.00863	-4.99852
H	0.43085	-0.88984	-5.06926
O	0.00727	-1.13436	-2.55865
N	0.02107	2.16596	1.34957
C	-1.15730	2.35984	2.02422
C	-1.13540	2.71564	3.38034
H	-2.05584	2.92520	3.89649
C	0.07209	2.86121	4.04560
H	0.09150	3.14560	5.08687
C	1.25458	2.69762	3.34035
H	2.19575	2.89162	3.82454
C	1.22452	2.34783	1.98355
C	-2.47923	2.29984	1.34682
C	-3.60390	2.00269	2.11556
H	-3.47467	1.61856	3.11549
C	-4.91815	2.20454	1.64996
H	-5.75042	1.95506	2.29081
C	-5.12826	2.76006	0.40987
H	-6.12527	2.98842	0.06095
C	-4.02492	3.02539	-0.44210
C	-4.20488	3.58248	-1.73318
H	-5.20153	3.84601	-2.05786
C	-3.12052	3.76620	-2.55933
H	-3.22123	4.19387	-3.54389
N	-1.64747	2.79045	-0.91331
C	-1.86459	3.31261	-2.13053
H	-1.03542	3.31257	-2.81391
C	-2.69526	2.71327	-0.00664
C	2.52041	2.28752	1.25798
C	3.67339	1.97009	1.97428
H	3.58356	1.56963	2.97222
C	4.96816	2.17441	1.45685
H	5.82524	1.90731	2.05658
C	5.13002	2.75607	0.22117
H	6.11313	2.98777	-0.16326
C	3.99367	3.04761	-0.57729
C	4.11770	3.64090	-1.85885
H	5.09997	3.90737	-2.22261
C	1.75817	3.39953	-2.15417
H	0.89602	3.44001	-2.79391
N	1.59642	2.83257	-0.94886
C	2.68318	2.72924	-0.09323
N	-0.01504	-2.16566	1.34989
C	1.16513	-2.35940	2.02140
C	1.14685	-2.71496	3.37763
H	2.06869	-2.92442	3.89135
C	-0.05884	-2.86042	4.04616
H	-0.07546	-3.14463	5.08753
C	-1.24323	-2.69697	3.34405
H	-2.18310	-2.89090	3.83080
C	-1.21680	-2.34742	1.98713
C	2.48519	-2.29949	1.34038
C	3.61192	-2.00215	2.10606
H	3.48534	-1.61785	3.10626
C	4.92492	-2.20404	1.63699
H	5.75889	-1.95445	2.27558
C	5.13172	-2.75969	0.39642
H	6.12779	-2.98808	0.04485
C	4.02612	-3.02511	-0.45258
C	4.20273	-3.58210	-1.74416
H	5.19855	-3.84546	-2.07150
C	3.11622	-3.76590	-2.56746
H	3.21439	-4.19349	-3.55230
C	1.86138	-3.31245	-2.13533
H	1.03039	-3.31271	-2.81653
N	1.64741	-2.79034	-0.91753
C	-2.51466	-2.28730	1.26501
C	-3.66578	-1.96975	1.98424
H	-3.57337	-1.56893	2.98180
C	-4.96188	-2.17440	1.47029
H	-5.81741	-1.90720	2.07217
C	-5.12694	-2.75658	0.23527
H	-6.11105	-2.98857	-0.14642
C	-3.99268	-3.04826	-0.56610
C	-4.11996	-3.64215	-1.84707
H	-5.10311	-3.90901	-2.20815
C	-3.00068	-3.86178	-2.61658
H	-3.06086	-4.32414	-3.58872
C	-1.76125	-3.40044	-2.14868
H	-0.90081	-3.44087	-2.79066
N	-1.59645	-2.83293	-0.94405
C	2.69759	-2.71310	-0.01360
C	-2.68097	-2.72948	-0.08561
H	-2.15969	-0.00428	-0.45679
H	2.16043	0.00434	-0.46538
O	-1.20755	-0.00513	-0.28447
O	1.20927	0.00517	-0.28762
C	2.99645	3.86046	-2.62551
H	3.05421	4.32240	-3.59799

Table S4: TD-DFT calculated electronic transitions with an oscillator strength > 0.015 for **Cr_2-ref**. Electron density difference maps (EDDMs) of the respective transitions are provided with electron density gain in red and electron density loss in blue.

Nr	Energy (cm^{-1})	λ (nm)	Osc. Strength	Transition	Electron density difference map
2	19722	507	0.0019	ILCT (dqp) & LMCT (dqp→Cr^{3+})	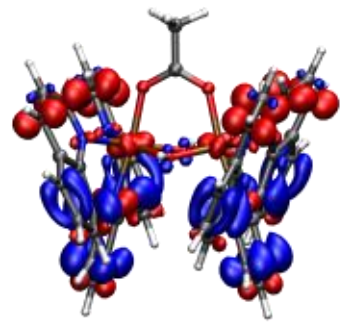
5	20595	486	0.0027	dd & LL'CT (OH^- & AcO^- →dqp)	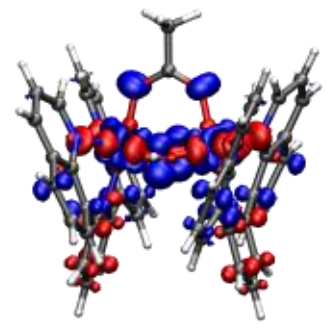
9	22005	454	0.0011	d-d & LL'CT (OH^- & AcO^- →dqp)	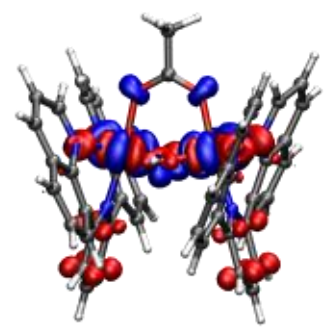
16	25836	387	0.1113	ILCT+LMCT (dqp)	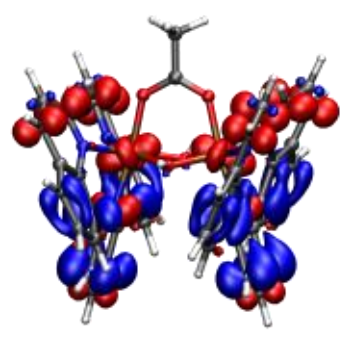
17	26390	379	0.3910	ILCT+LMCT (dqp)	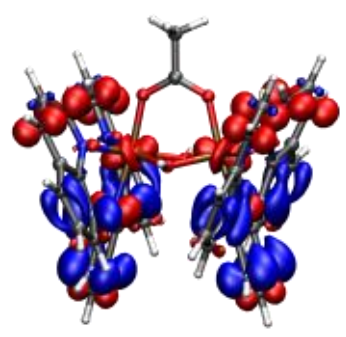

22	27229	367	0.0163	ILCT+ LMCT<br>(dqp)	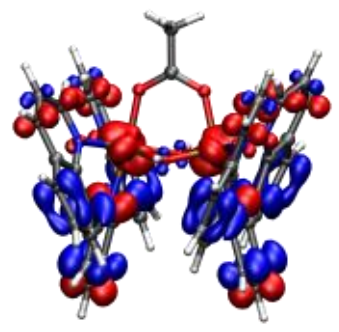
27	28271	354	0.0018	ILCT+ LMCT<br>(dqp)	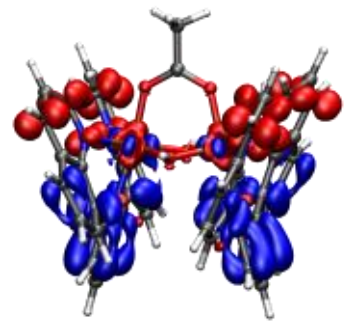
30	28446	352	0.0019	π→ π* (dqp)<br>& LMCT<br>(OH^{-}→Cr^{3+})	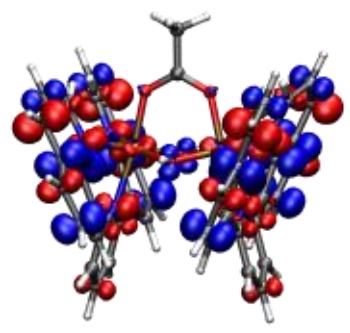
31	28449	352	0.0024	π→ π* (dqp)<br>& LMCT<br>(OH^{-}→Cr^{3+})	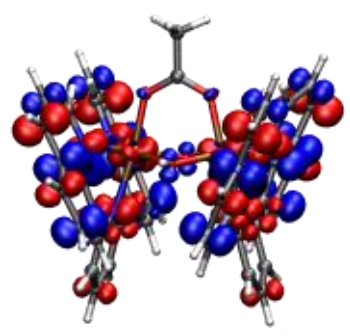
32	28594	350	0.0152	ILCT & LMCT<br>(dqp)	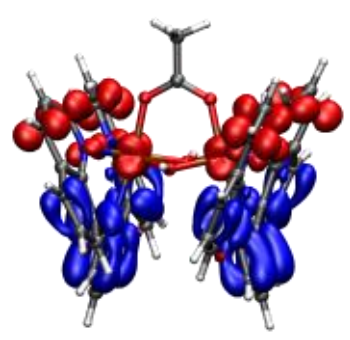
33	28867	346	0.0018	ILCT & LMCT<br>(dqp & OH^{-})	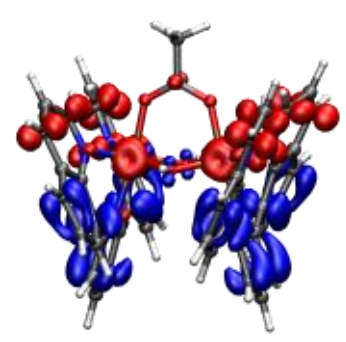

34	28912	346	0.0037	ILCT & LMCT (dqp & OH^-)	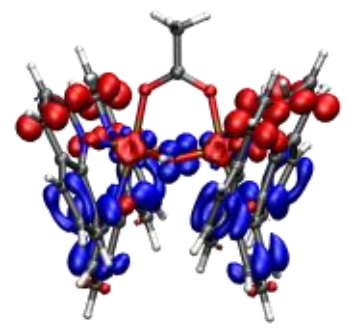
35	28988	345	0.0010	ILCT & LMCT (dqp & OH^-)	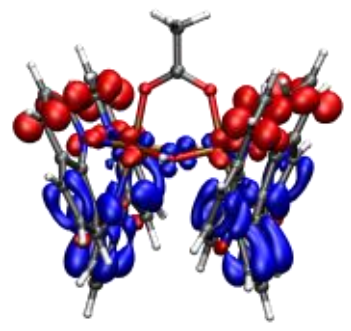
36	29028	344	0.0028	ILCT & LMCT (dqp & OH^-)	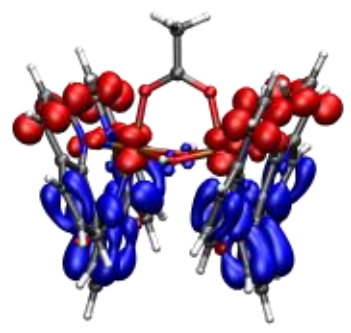
40	29716	337	0.0012	ILCT (dqp)	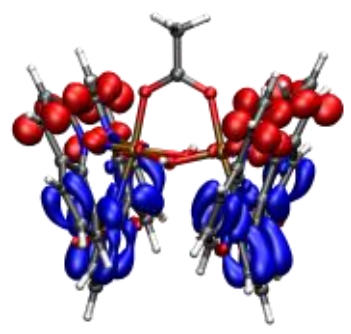
41	29921	334	0.0325	LL'CT (OH^-→dqp) & MLCT (Cr^{3+}→dqp)	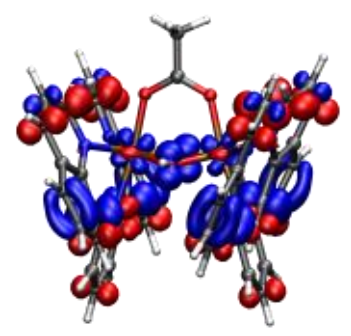
42	30134	332	0.0153	LL'CT (OH^-→dqp) & MLCT (Cr^{3+}→dqp)	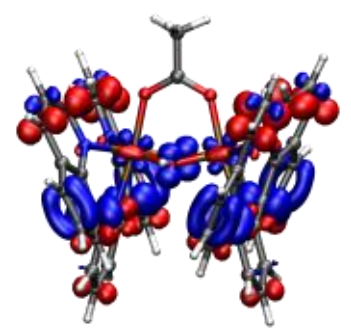

43	30790	325	0.0011	LL'CT (OH^-→dqp) & MLCT (Cr^{3+}→dqp)	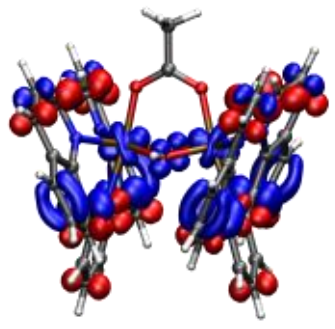
45	31497	317	0.1671	LL'CT, MLCT & LMCT	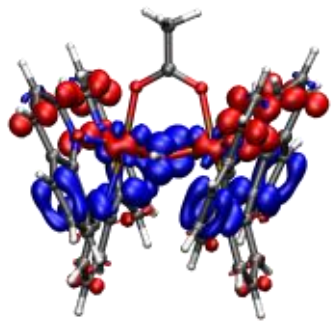
46	31588	317	0.0059	ILCT (dqp) & MLCT (Cr^{3+}→dqp)	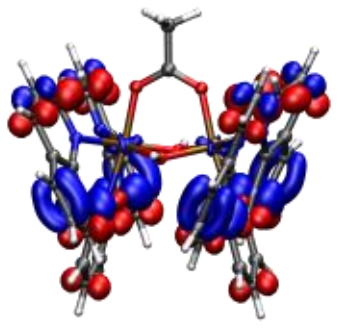
47	31620	316	0.0026	LL'CT (dqp→OH^-) & LMCT (dqp→Cr^{3+})	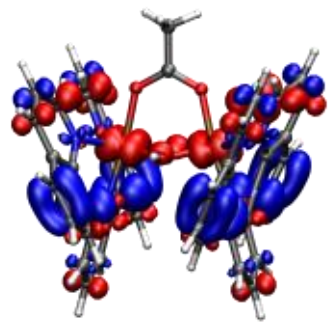
50	31881	314	0.0029	LL'CT (dqp→OH^-) & LMCT (dqp→Cr^{3+})	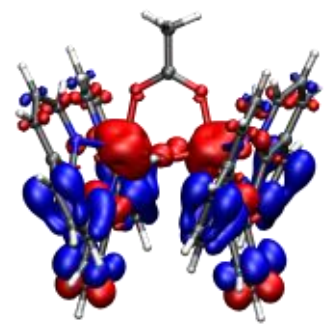
51	31929	313	0.0019	LL'CT (dqp→OH^-) & LMCT (dqp→Cr^{3+})	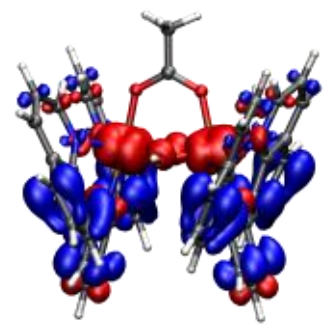

52	32090	312	0.0013	ILCT (dqp) & LMCT (dqp→Cr^{3+})	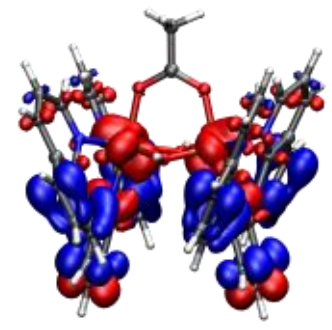
54	32197	311	0.0061	LL'CT (dqp→OH^-) & LMCT (dqp→Cr^{3+})	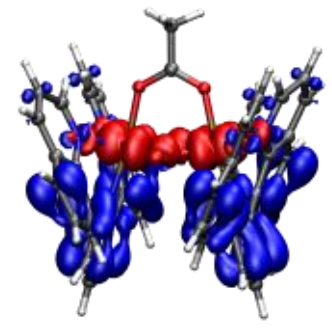
56	32535	307	0.0036	LL'CT (dqp→OH^- &AcO^-) & LMCT (dqp→Cr^{3+})	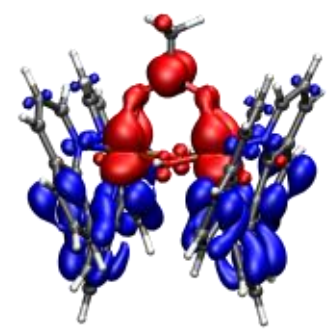
57	32866	304	0.0109	LL'CT (dqp→OH^- &AcO^-) & LMCT (dqp→Cr^{3+})	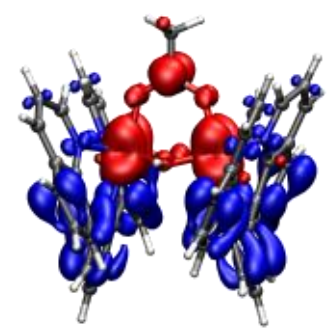
58	33024	303	0.1118	ILCT (dqp) & MLCT (Cr^{3+}→dqp)	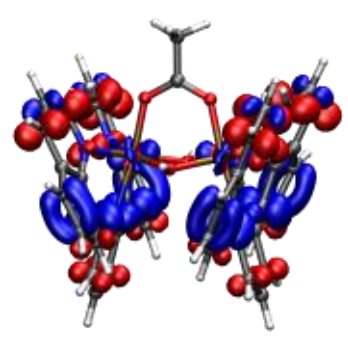
60	33253	301	0.0028	ILCT (dqp) LMCT (AcO^-& dqp→Cr^{3+})	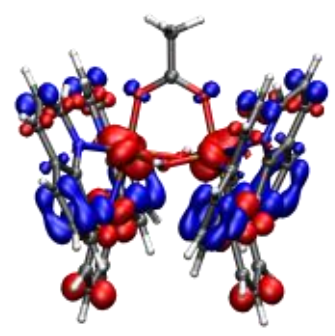

Table S5: Atomic coordinates of the dimeric anthracene fragment $(PA)_2$ for TD-DFT calculations.

C	4.19870	24.66600	8.59130
C	3.91180	23.92540	9.74150
C	3.79010	24.53950	11.03650
H	3.88030	25.48090	11.11460
C	3.54960	23.80170	12.15050
H	3.47660	24.23140	12.99400
C	3.40730	22.38750	12.06520
H	3.21440	21.87990	12.84500
C	3.54420	21.77420	10.87440
H	3.48390	20.82710	10.83390
C	3.78040	22.51040	9.66490
C	3.87500	21.86640	8.42060
C	4.08530	22.61730	7.25010
C	4.12650	22.00570	5.94360
H	4.01160	21.06470	5.85970
C	4.33120	22.76190	4.82950
H	4.33320	22.34290	3.97590
C	4.53600	24.15110	4.91920
H	4.68170	24.66150	4.13220
C	4.52710	24.74760	6.10710
H	4.69080	25.68160	6.15190
C	4.27670	24.02450	7.32820
C	3.75480	20.37850	8.33810
C	2.53000	19.77350	8.00100
H	1.76860	20.29870	7.79120
C	2.46050	18.34460	7.98510
H	1.63130	17.92260	7.78540
C	3.52720	17.58670	8.24120
H	3.46600	16.64040	8.18470
C	4.71520	18.18440	8.58840
H	5.46450	17.64410	8.80110
C	4.83840	19.58130	8.63180
H	5.67090	19.97910	8.86480
C	6.80220	24.66600	13.11120
C	7.08910	23.92540	11.96100
C	7.21080	24.53950	10.66610
H	7.12060	25.48090	10.58790
C	7.45130	23.80170	9.55200
H	7.52430	24.23140	8.70850
C	7.59360	22.38750	9.63740
H	7.78650	21.87990	8.85750
C	7.45670	21.77420	10.82810
H	7.51690	20.82710	10.86860
C	7.22050	22.51040	12.03770
C	7.12590	21.86640	13.28190
C	6.91560	22.61730	14.45240
C	6.87440	22.00570	15.75890
H	6.98930	21.06470	15.84280
C	6.66970	22.76190	16.87300
H	6.66770	22.34290	17.72660
C	6.46480	24.15110	16.78330
H	6.31920	24.66150	17.57040
C	6.47370	24.74760	15.59540
H	6.31010	25.68160	15.55060
C	6.72420	24.02450	14.37430
C	7.24610	20.37850	13.36440
C	8.47090	19.77350	13.70150
H	9.23230	20.29870	13.91130
C	8.54040	18.34460	13.71740
H	9.36960	17.92260	13.91710
C	7.47370	17.58670	13.46140
H	7.53490	16.64040	13.51780
C	6.28560	18.18440	13.11410
H	5.53640	17.64410	12.90140
C	6.16240	19.58130	13.07070
H	5.33000	19.97910	12.83780
H	4.33570	25.72420	8.67100
H	6.66520	25.72420	13.03150

Table S6: Relevant TD-DFT calculated electronic transitions with an oscillator strength > 0.1 for the anthracene fragments $(PA)_2$ in **Cr_2-An**. Electron density difference maps (EDDMs) of the respective transitions are provided with electron density gain in red and electron density loss in blue.

Nr	Energy (cm^{-1})	λ (nm)	Osc. Strength	Transition	Electron density difference map
1	23648	423	0.1018	Anthracene-based π→ π*	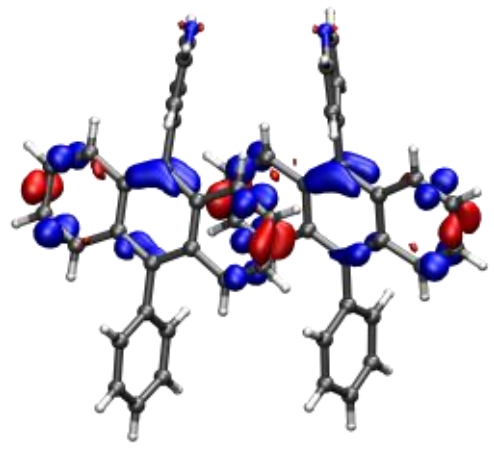
4	27514	363	0.2531	Anthracene-based π→ π*	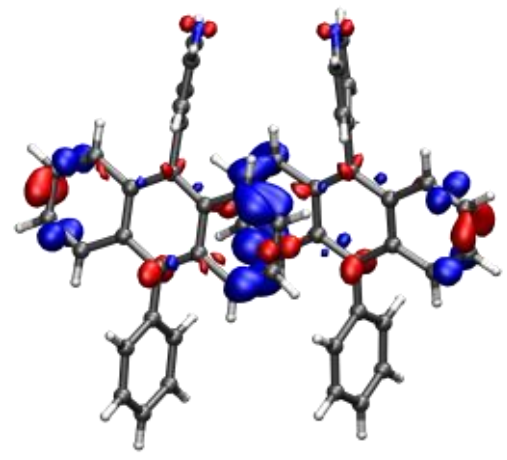

The calculated absorption spectrum of **Cr_2-ref** (Figure S17) reproduces the experimental absorption spectrum very well. The nature of the calculated transitions is in line with previous calculations in mononuclear chromium(III) polypyridine complexes with metal-centered d-d transitions in the visible region, LMCT transitions in the blue, and ligand-centered and MLCT transitions in the UV.[37-39] Chromium(III) dimers employing the same AcO^- and OH^- bridging units as in the present work have not been investigated by TD-DFT calculations with regards to their UV-Vis absorption data, though it was generally assumed that the nature of electronic transitions was equivalent to mononuclear chromium(III) complexes.[23, 32] Due to the complexity of the **Cr_2-An** system, TD-DFT calculations on **Cr_2-An** did not yield any meaningful results, assumed to stem from spin incompatibility of the (assumed) singlet states on anthracene and measured septet state of **Cr_2An**. Because both complexes have their experimental absorption maximum at almost the same wavelength (363 nm / 366 nm), the assumption that **Cr_2-An** has the same transitions as **Cr_2-ref** with additional transitions stemming from the attached PA units seems plausible. Therefore, the atomic coordinates of the dimeric phenylanthracenylpyridine subunit of **Cr_2An** were extracted from the crystal structure and used for TD-DFT calculations. The resulting calculation shows two significant π-π* transitions, which are lower in energy than those for monomeric phenylanthracenylpyridine due to the proximity of the two systems. This red-shift of dimeric or aggregated π-systems is a common observation and a characteristic feature often exploited in the design of aggregation-induced emission compounds.[13, 46] A calculated UV-Vis absorption spectrum reproduces the experimental difference spectrum (**Cr_2-An** - **Cr_2-ref**) very well (Figure S18). A comparison between the experimental spectrum of **Cr_2-An** and a linear combination of the calculated spectra (**Cr_2-ref** + $(PA)_2$) is shown in Figure S19. The linear combination mostly reproduces the experimental data, though the strong visible absorption in **Cr_2-An** seems underrepresented in the calculation, likely due to differences between the solid-state structure used for TD-DFT calculations and the structure in solution for the dimeric anthracene unit. The $(PA)_2$ fragment seems to act as an antenna in **Cr_2-An** for the observed strong visible light absorption. Intraligand charge transfer between the anthracenes and the dqp framework might be viable and could contribute to the strong visible absorption, but our attempt with theoretical calculations for **Cr_2-An** failed to provide the evidence for this hypothesis. Based on our current calculations, we attribute the strong absorption to the antenna effect of $(PA)_2$.

8. Transient absorption spectroscopy

8.1 fs-transient absorption spectroscopy

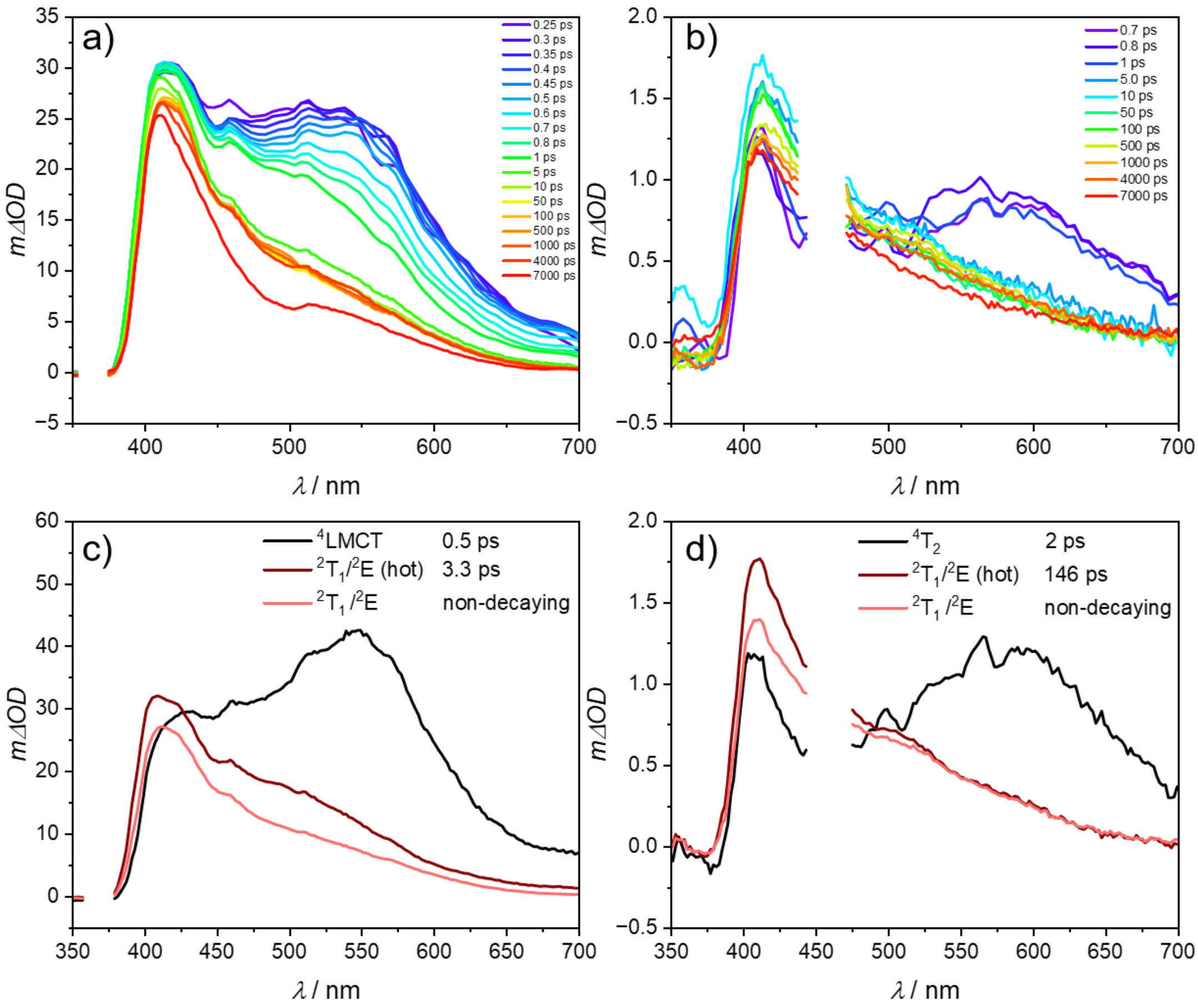

Figure S20: UV-Vis transient absorption (TA) spectra of **Cr_2-ref** (100 µM) in deaerated acetonitrile at 20 °C. The spectra were recorded at different time delays after excitation with a fs-pulsed laser at a) 370 nm or b) 460 nm. The wavelength region around the excitation wavelengths is removed due to pump scattering. The corresponding species-associated spectra with their time constants are shown in the bottom panels for c) 370 nm and d) 460 nm excitation.

The fs-TA spectra of **Cr_2-ref** show broad excited state absorption (ESA) bands between 380 nm and 700 nm upon 370 nm excitation (Figure S20a). Based on the absorption data and DFT calculations, excitation at 370 nm primarily populates a ^{4}LMCT state. Global analysis of the spectra based on a sequential model yields three states (Figure S20c). The first state shows excited state absorption at 550 nm with a shoulder at 410 nm, which decays with a time constant of 0.5 ps. The lifetime of 0.5 ps is likely attributed to intersystem crossing (ISC) from the ^{4}LMCT state to the doublet excited states manifolds. This populates an intermediate state maximized at 410 nm, which decays with a lifetime of 3.3 ps into the final state. The latter does not decay on the timescale (7 ns) of the fs-TA experiment. Both the intermediate state and the final state share the same spectral features, which indicates that they correspond to states of similar nature. As their spectral shape is essentially the same as observed in ns-TA measurements (section 8.2), this allows for their assignment to the luminescent $^2T_1/^2E$ states. The intermediate state corresponds to a “hot” $^2T_1/^2E$ state, which decays into the luminescent $^2T_1/^2E$ states through internal conversion (IC) and vibrational relaxation (VR) with a time constant of 3.3 ps. These spectral features and time

constants agree well with the fs-TA analysis of the mononuclear complex $[Cr(bpmp)_2]^{3+}$ upon excitation into the ^{4}LMCT state.[39]

Excitation of **Cr_2-ref** at 460 nm yields partially distinct TA features (Figure S20b/d). Based on the absorption data and DFT calculations, excitation at 460 nm leads to population of the metal-centered $^4T_2$ state. The first state observed in species-associated spectra shows excited state absorption bands centered at 410 nm and 590 nm and decays with a lifetime of 2 ps. For the mononuclear $[Cr(ddpd)_2]^{3+}$, fs-laser excitation into the $^4T_2$ state reveals a combined ISC, IC, and VR process with a time constant of 3.3 ps,[47] whereas $[Cr(bpmp)_2]^{3+}$ shows an ultrafast ISC (<200 fs) and slow IC and VR processes (300 ps) to the final non-decaying states upon excitation into the $^4T_2$ state.[39] For **Cr_2-ref**, the initial process of 2 ps seems long for an ISC, likely due to minor contribution from IC and VR. The subsequent intermediate and non-decaying states share the same characteristics as observed upon excitation at 370 nm, though the IC and VR process shows a significantly longer lifetime (146 ps).A simplified Jablonski diagram for **Cr_2-ref** is shown in Figure S25a.

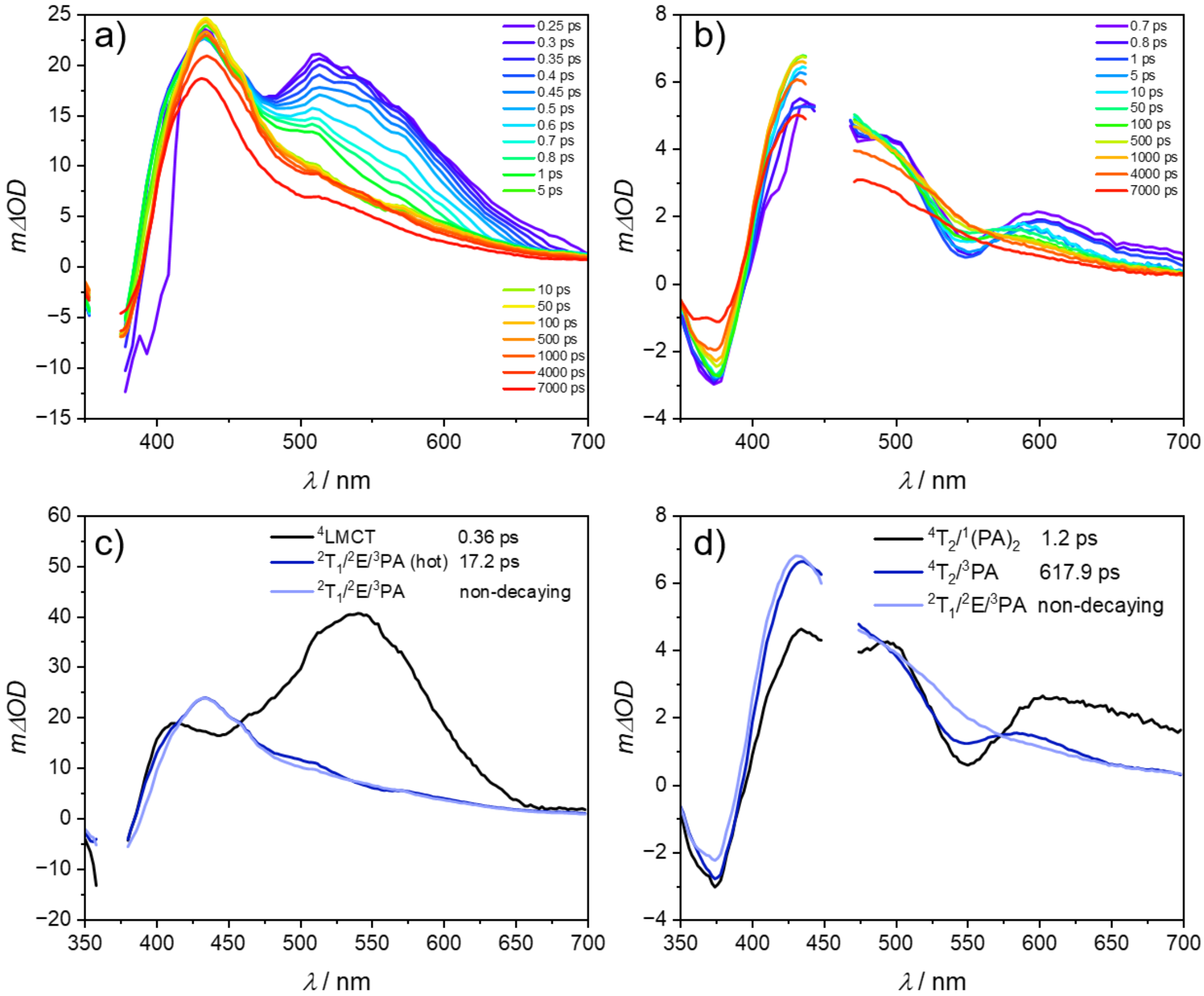

Figure S21: UV-Vis transient absorption spectra of **Cr_2-An** (80 µM) in deaerated acetonitrile at 20 °C. The spectra were recorded at different time delays after excitation with a fs-pulsed laser at a) 370 nm or b) 460 nm. The wavelength region around the excitation wavelengths is removed due to pump scattering. The corresponding species-associated spectra with the corresponding time constants are shown in the bottom panels for c) 370 nm and d) 460 nm excitation.

The fs-TA spectra of **Cr_2-An** upon excitation at 370 nm (Figure S21a) look similar to those of **Cr_2-ref** at first glance. According to the global analysis, the first state in the species-associated spectra (Figure S21c) is very similar to the ^{4}LMCT state observed in **Cr_2-ref** with a lifetime of

0.36 ps. This time constant likely accounts for the ISC from the ^{4}LMCT state to the doublet hot intermediate state and the ISC from $^1(PA)_2$ to the ^{3}PA* intermediate state. The mixed hot intermediate state shows a lifetime of 17.2 ps and shares the spectral features with the non-decaying state. Notably, for both the intermediate state and the final states, their excited state absorption is shifted to 430 nm instead of 410 nm as observed in **Cr_2-ref** (Figure S20c). This difference is more pronounced in the ns-TA spectra for both complexes (Figure S24a). The transient absorption signal at 430 nm is characteristic for a ^{3}PA* state that was previously observed in various metal complexes with anchored PA units.[22, 48, 49] For the intermediate and the final states, the broad TA band from 500 to 700 nm, which is not present for ^{3}PA*,[48] represents the spin-flip excited states as observed in **Cr_2-ref**. This suggests the co-existence of both the spin-flip excited states and the ^{3}PA* state shortly following the initial state. Considering the almost identical energy level between the spin-flip states and the ^{3}PA state (Table 1 in the main paper), doublet-triplet energy transfer (DTET) from the doublet spin-flip excited states to ^{3}PA* is thermodynamically feasible. The intramolecular DTET accounts likely for the much longer time constant (17.2 ps) for the intermediate hot state than that observed in **Cr_2-ref** (3.3 ps, Figure S20c). For the formation of ^{3}PA*, we propose two potential pathways: i) direct ISC from the populated singlet state located on anthracenes $^1(PA)_2$ (Figure S18), which remains likely under the cover of the strong TA signal of the ^{4}LMCT state; ii) intramolecular DTET from the doublet spin-flip excited states to ^{3}PA*.

Excitation of **Cr_2-An** at 460 nm (Figure S21b) leads to different TA spectra as compared to **Cr_2-ref**. Absorption spectra and DFT calculations suggest that excitation at this wavelength populates the $^4T_2$ state as well a π–π* $^1(PA)_2$ state, and the latter contributes dominantly due to the substantially higher extinction coefficients in the visible region. Notably, the initial state shows a TA maximum at 430 nm, accompanied with a TA band at around 600 nm from the $^4T_2$ state and an additional shoulder at 500 nm (Figure S21d). The latter is likely attributed to the singlet excited state located on the anthracene fragment, considering the additional π–π* transition in $^1(PA)_2$ in this wavelength region. This initial mixed state decays into an intermediate state with a lifetime of 1.2 ps, while the TA signal at 430 nm grows rapidly. This intermediate state retains the two distinct TA bands at 430 nm and 600 nm but lacks that at 500 nm. The rapid disappearance of the TA signal at 500 nm and growth at 430 nm is likely attributed to a direct ISC from the $^1(PA)_2$* state to the ^{3}PA* state. This hypothesis is further supported by the excitation spectrum for **Cr_2-An** (Figure S9), which evidences the transition from the $^1(PA)_2$* state to the emissive ^{3}PA* state. The presence of the chromium ions might account for this phenomenon, although they are not considered as traditional heavy atoms.

The intermediate state decays with a lifetime of 618 ps and yields the same mixed non-decaying $^2T_1$/^{2}E/^{3}PA excited states as that observed upon 370 nm excitation (Figure S21c), and the final state agrees well with the ns-TA spectrum (Figure S23a). This slow process is presumably attributed to the triplet-to-doublet energy transfer (TDET) from the ^{3}PA* state to the $^2T_1$/^{2}E excited states that are almost energetically identical (Table 1 in the main paper), leading to an equilibrated mixture of both states. Under 460 nm excitation, the TA signal at 600 nm, which is attributed to the $^4T_2$ state as seen in Cr_2-ref, is observed. The slow diminishing of this signal and the continuous growth of the TA band at 430 nm might suggest the occurrence of ISC from the $^4T_2$ state to the doublet spin-flip excited states, followed by DTET to ^{3}PA*. Both pathways seem viable for the population of ^{3}PA* under these conditions. A summarized excited state dynamics is shown in Figure S25. DTET via charge separation and subsequent recombination as recently reported for a Fe^{III}-PA dyad[48] seems unlikely, since no clear charge separated species were observed in the fs-TA spectra.

8.2 ns-transient absorption spectroscopy

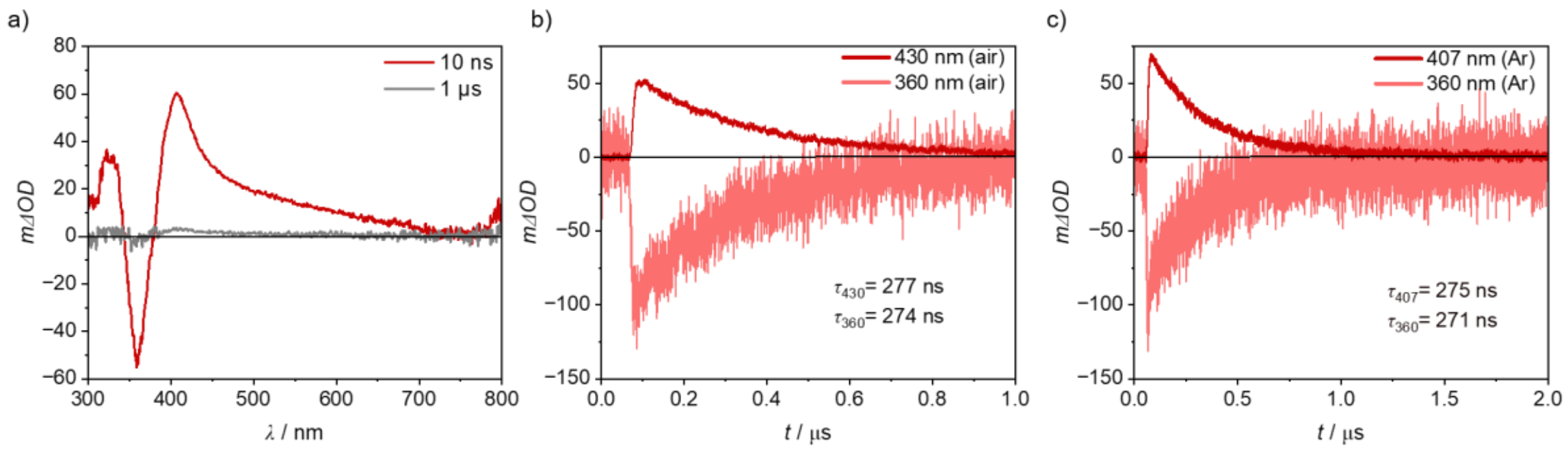

Figure S22: UV-Vis transient absorption measurements for **Cr_2-ref** (50 µM) in acetonitrile at 20 °C under ns-pulsed laser excitation at 355 nm. a) Transient absorption spectra of **Cr_2-ref** in aerated acetonitrile with different time delays after laser excitation, b) transient absorption decay kinetics of the sample from a) recorded at 360 nm (light red traces) and 430 nm (dark red traces), c) transient absorption decay kinetics of **Cr_2-ref** in deaerated acetonitrile recorded at 407 nm (dark red traces) and 360 nm (light red traces). All decay kinetics are fitted monoexponentially, giving time constant of ~275 ns that shows minor sensitivity to oxygen.

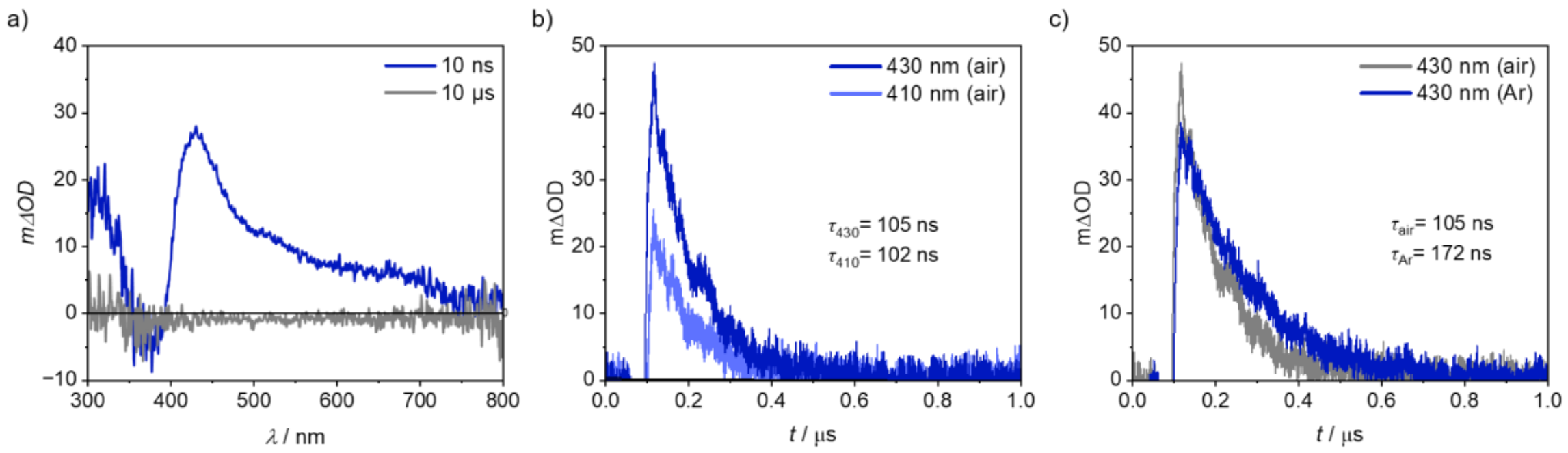

Figure S23: UV-Vis transient absorption measurements for **Cr_2-An** (50 µM) in acetonitrile at 20 °C under ns-pulsed laser excitation at 355 nm. a) Transient absorption spectra of **Cr_2-An** in aerated acetonitrile with different time delays after laser excitation, b) transient absorption decay kinetics of the sample from a) recorded at 410 nm (light blue traces) and 430 nm (dark blue traces), c) transient absorption decay kinetics of **Cr_2-An** in aerated (gray traces) and deaerated (blue traces) acetonitrile recorded at 430 nm. All decay kinetics are fitted monoexponentially, giving time constants of ~105 ns in aerated and ~170 ns in deaerated acetonitrile.

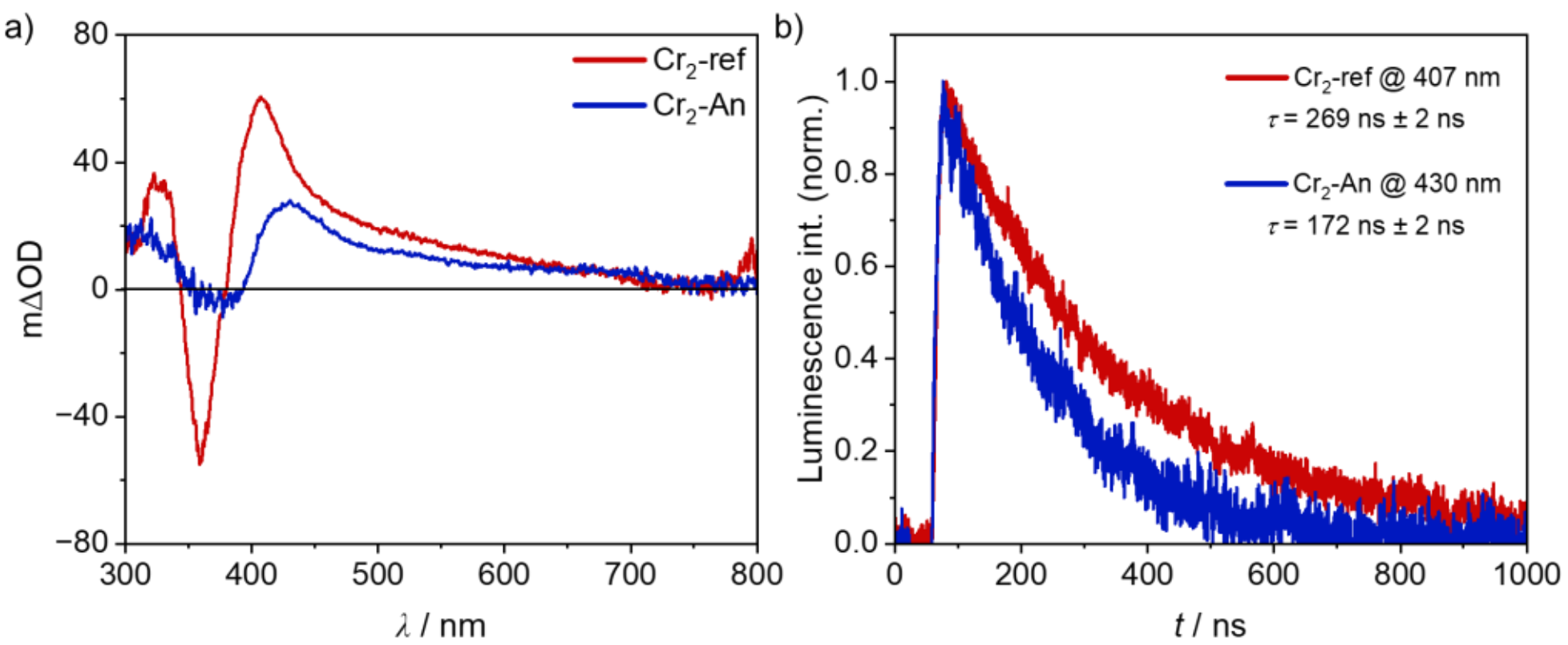

Figure S24: a) UV-Vis transient absorption spectra of **Cr_2-ref** (red, 50 µM) and **Cr_2-An** (blue, 50 µM) in aerated acetonitrile at 20 °C at a fixed time delay of 10 ns after excitation with a ns-pulsed laser at 355 nm. b) Normalized decays of the transient absorption signals from a) under deaerated conditions at their respective maximum.

The ns-TA spectrum of **Cr_2-ref** with 10 ns delay after excitation at 355 nm essentially shows the same spectral features as the non-decaying state in the species associated spectra for the fs-TA experiments. TA signal decays measured at wavelengths corresponding to the ground state bleach at 360 nm and excited state absorption show a lifetime of ~275 ns under both aerated and deaerated conditions. This lifetime is in excellent agreement with the luminescence lifetimes measured for **Cr_2-ref** (section 6.1). This indicates that the final state observed in the fs-TA experiments corresponds to the luminescent $^2T_1{}^4A_2/^2E^4A_2$ states.

For **Cr_2-An**, the ns-TA spectrum after 355 nm excitation also shows good agreement with the non-decaying state observed in the fs-TA experiments. The TA signal decays measured at the characteristic excited state absorption band at 430 nm show a clear oxygen sensitivity, due to the triplet nature of $^3PA^*$. The corresponding TA lifetimes under both aerated and deaerated conditions closely the luminescence lifetimes (section 6.2). A comparison of the TA signal decays of **Cr_2-An** measured at 410 nm and 430 nm shows the same kinetics for both wavelengths. As the band at 410 nm corresponds to the $^2T_1{}^4A_2/^2E^4A_2$ state in **Cr_2-ref**, the identical kinetics suggest an excited state equilibrium of $^2T_1{}^4A_2/^2E^4A_2$ and $^3PA^*$ in **Cr_2-An**. Low-temperature luminescence measurements further evidence the thermal equilibrium between these excited states (Figure S8 and Figure S13). At room temperature, significant population seems to locate on $^3PA^*$, since no clear luminescence signal from the spin-flip excited states is observed under these conditions (section 6.2).

Based on the spectroscopic investigation discussed above, the simplified Jablonski diagrams for **Cr_2-ref** and **Cr_2-An** are shown in Figure S25.

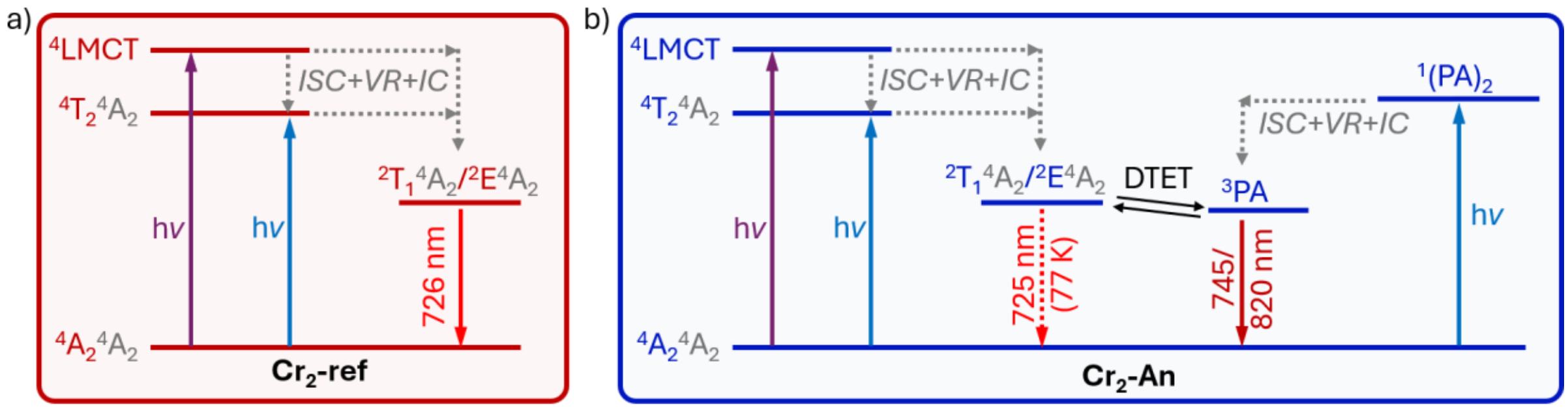

Figure S25: Simplified Jablonski diagrams for **Cr_2-ref** (a) and **Cr_2-An** (b). ISC: intersystem crossing, VR: vibrational relaxation, IC: internal conversion, DTET: doublet-triplet energy transfer.

9. Triplet-triplet annihilation upconversion

9.1 Stern-Volmer studies

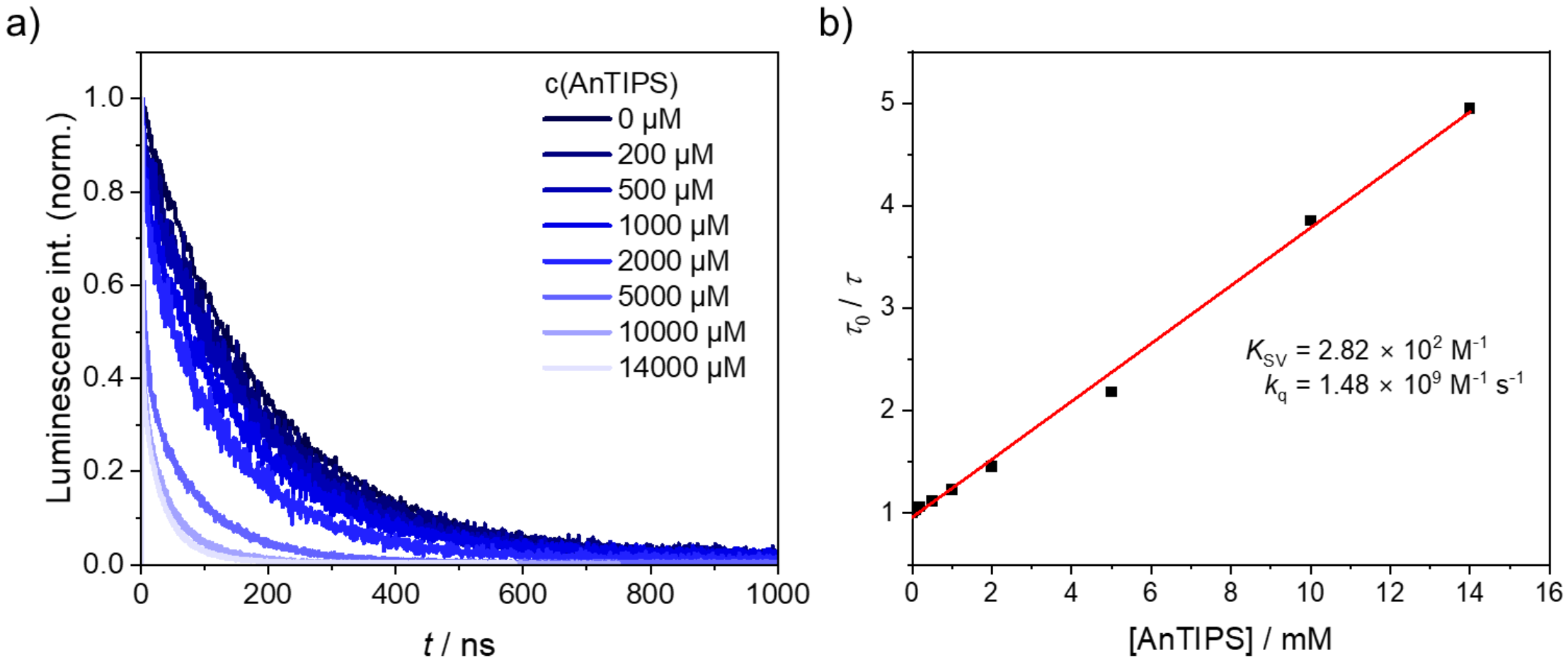

Figure S26: Luminescence lifetime-based Stern-Volmer quenching of **Cr_2-An** (100 µM) by AnTIPS in deaerated 1,2-dichloroethane at 20 °C. a) Normalized luminescence decay of **Cr_2-An** at 745 nm after excitation at 485 nm with varying concentrations of AnTIPS. b) Corresponding Stern-Volmer plot derived from the luminescence lifetime values in a). A linear fit gives the Stern-Volmer constant K_{SV} from the slope. The quenching rate constant k_q can be calculated from $k_q = K_{SV} / \tau_0$ (initial lifetime τ_0 = 190 ns from a)).

9.2 Control experiments

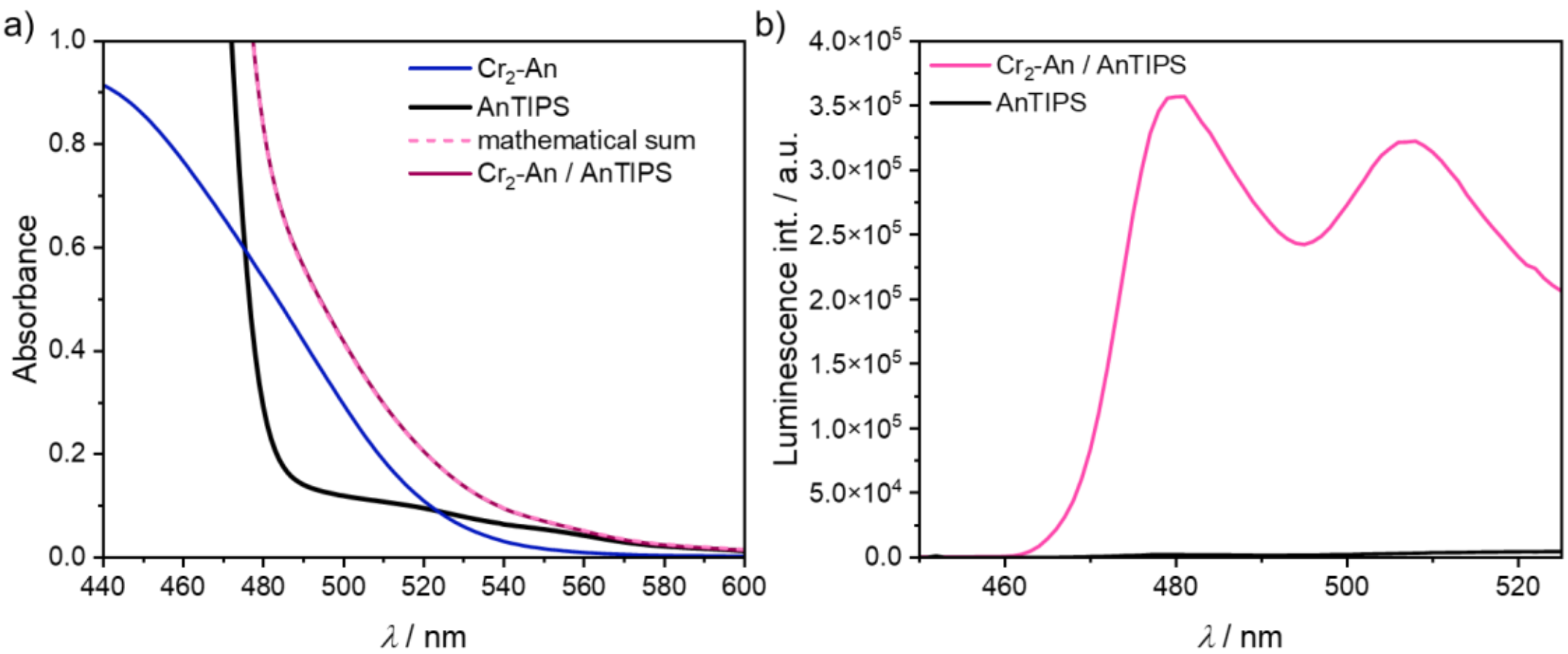

Figure S27: a) UV-Vis absorption spectra of **Cr_2-An** (100 µM, blue trace), AnTIPS (20 mM, black trace), the mathematical sum of these two spectra (dashed pink trace) and the upconversion sample (magenta trace) containing **Cr_2-An** (100 µM) and AnTIPS (20 mM). All spectra were recorded in 1,2-dichloroethane at 20 °C. b) Luminescence spectra of the upconversion sample (100 µM **Cr_2-An**, 20 mM AnTIPS, pink trace) and AnTIPS (20 mM, black trace) upon excitation at 532 nm with a CW-laser (200 mW). Both spectra were measured in deaerated 1,2-dichloroethane at 20 °C.

9.3 Upconversion experiments

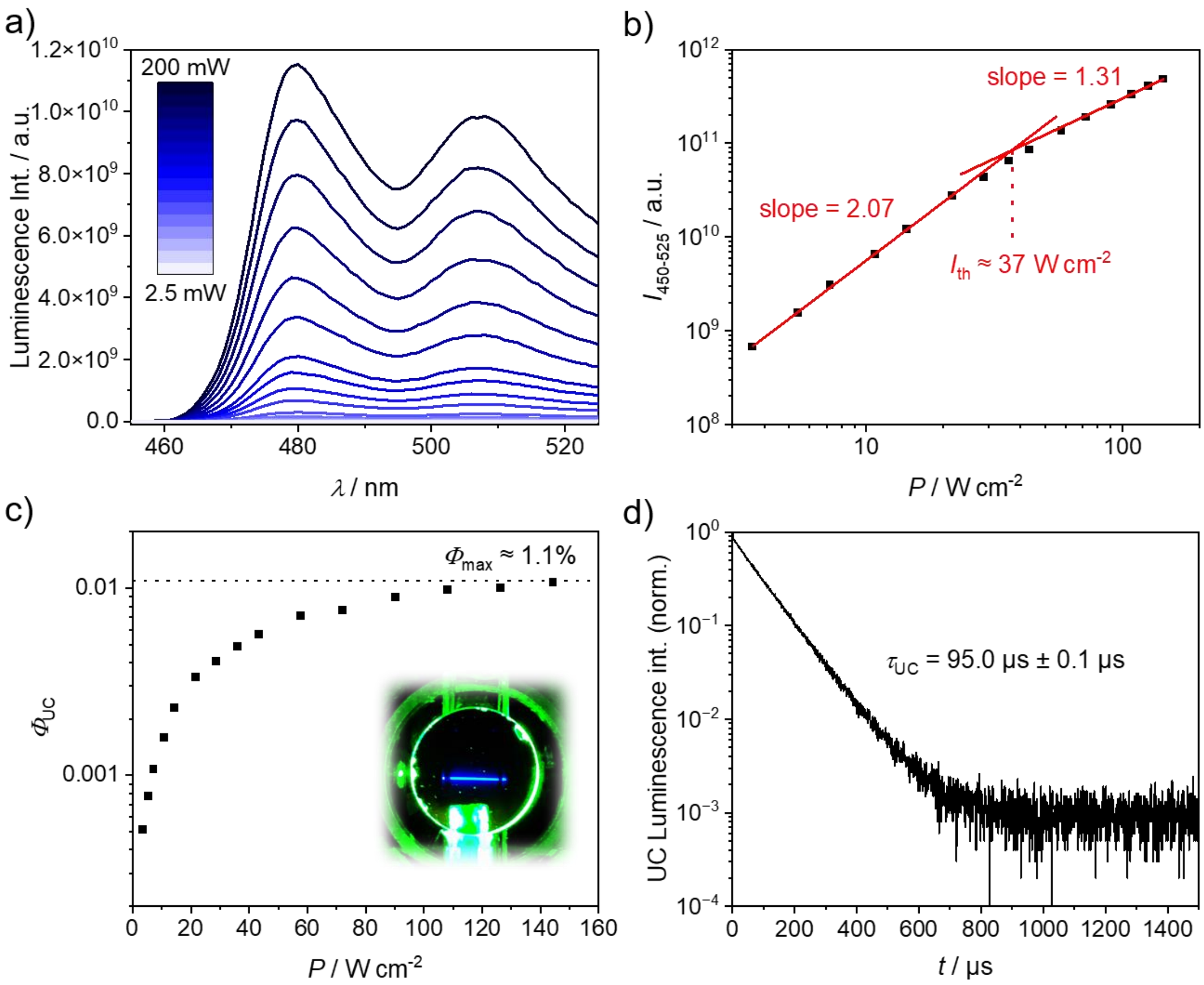

Figure S28: a) Upconversion luminescence spectra of **Cr_2-An** (100 µM) / AnTIPS (20 mM) in deaerated 1,2-dichloroethane at 20 °C upon excitation at 532 nm with a CW-laser at different laser powers (2.5 mW – 200 mW) b) Bi-logarithmic plot of the upconversion luminescence (integrated from 450 to 525 nm, form a)) in dependence of the excitation power density. At the crosspoint of the linear fits in the biphotonic regime (slope = 2.07) and the near saturated regime (slope = 1.31), the excitation power density is determined to 37 W cm^{-1}. The corresponding threshold value I_{th} is expected to be slightly higher than this value. c) Upconversion luminescence quantum yield of the sample from a) as a function of the respective excitation power density. The quantum yield was determined by relative luminescence intensity measurements from a) with a reference sample of $[Ru(bpy)_3]Cl_2$ (15 µM in deaerated water); inset: image of the sample from a) under 532 nm excitation (144 W cm^{-2}) with a 480 nm shortpass filter placed in front of the sample. d) Normalized upconversion luminescence decay of the sample from a), detected at 475 nm upon excitation with a 520 nm CW-laser that is monitored in a pulsed mode (121 mW, 700 µs pulse width).

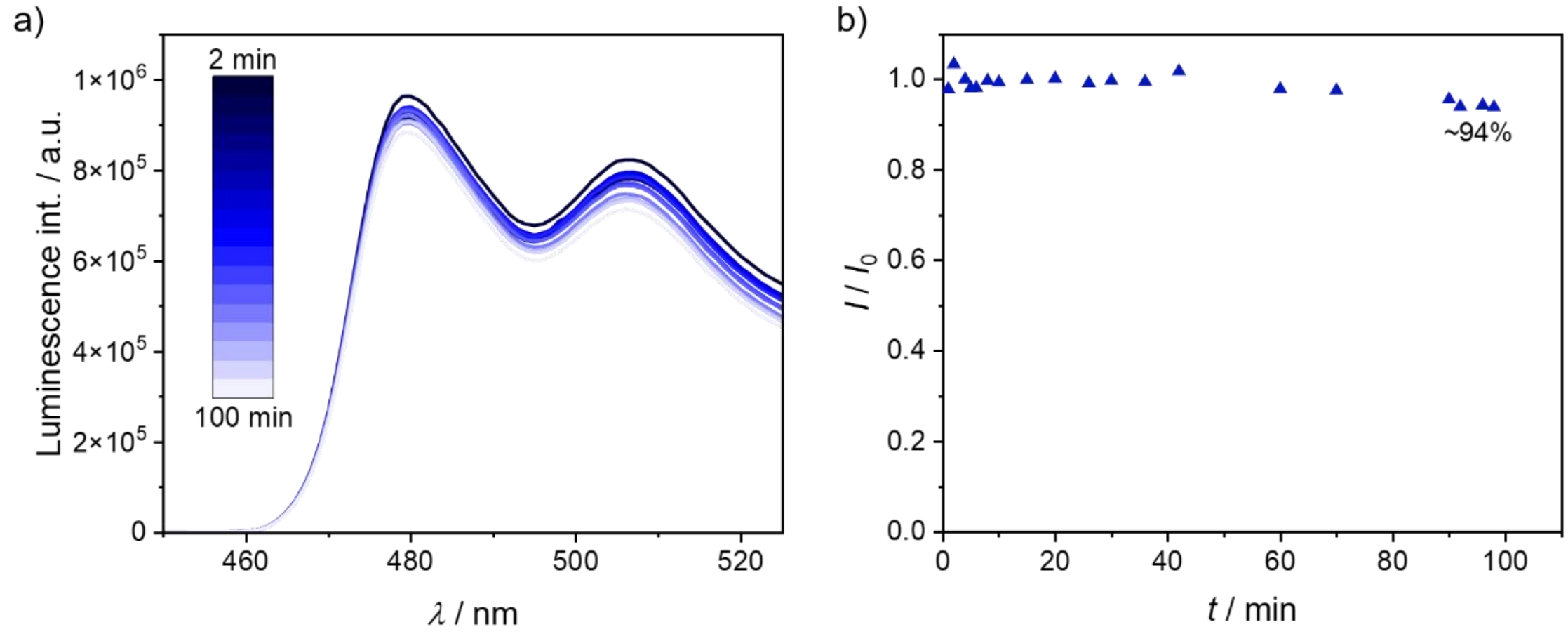

Figure S29: a) Upconversion luminescence spectra of **Cr$_2$-An** (100 µM) / AnTIPS (20 mM) in deaerated 1,2-dichloroethane at 20 °C measured at multiple time points (0 – 100 min) under continuous CW-laser irradiation (144 W cm^{-2}) at 532 nm. b) Integrated luminescence intensity ($I_{450\text{-}525}$) from a) in relation to the initial luminescence intensity at time zero I_0, plotted as a function of the irradiation time. The luminescence intensity remains ~ 94% of the initial intensity after 100 min of continuous laser irradiation, suggesting a high photostability of our upconversion system.

UV-Vis absorption spectra of the upconversion sample (100 µM **Cr$_2$-An** / 20 mM AnTIPS) as well as **Cr$_2$-An** and AnTIPS show that the absorption of the upconversion sample can be expressed as the linear combination of the individual components. This means that at the wavelength used for the upconversion experiments (532 nm), only about half of the incident light is absorbed by the sensitizer **Cr$_2$-An**. This needs to be taken into account for the calculation of the upconversion luminescence quantum yield. As the direct excitation of AnTIPS with the 532 nm laser used for the upconversion experiments only yields about 1% of the luminescence compared to the upconversion sample, this was not taken into account for the quantum yield calculation due to its miniscule effect.

The upconversion luminescence spectra were recorded with increasing laser power. Noticeably, the emission lacks a band at 450 nm, which would be expected from ^{1}AnTIPS fluorescence. This can be attributed to the inner filter effect due to re-absorption of the light at such high concentrations of AnTIPS.[26, 50] A plot of the integrated upconversion luminescence intensity against the laser power density shows a linear trend with a slope of 2.07 at lower power densities. This is indicative of the biphotonic nature of the upconversion process. The reduction of the slope at higher power densities indicates saturation of the upconversion system and usually leads to a slope of 1. As the slope only reaches a value of 1.31, the system did not achieve the fully saturated regime at the highest power setting of the utilized laser.

Based on the measured upconversion luminescence spectra, the upconversion luminescence spectra quantum yield was determined as described in section 1 by a relative method with $[Ru(bpy)_3]Cl_2$ as a reference. The equation for calculating the upconversion luminescence quantum yield Φ_{UC} was adjusted due to the discussed non-negligible absorption of AnTIPS at 532 nm:

$$\Phi_{\mathrm{UC}} = \Phi_{\mathrm{ref}} \cdot \frac{A_{\mathrm{ref}}}{A_{\mathrm{UC}} - A_{\mathrm{AnTIPS}}} \cdot \frac{I_{\mathrm{UC}}}{I_{\mathrm{ref}}} \cdot \left(\frac{\eta_{\mathrm{UC}}}{\eta_{\mathrm{ref}}}\right)^2 \qquad eq.(S3)$$

A_{ref}, A_{UC} and A_{AnTIPS} represent the absorbance of the reference, upconversion and AnTIPS sample at 532 nm respectively. I_{ref} and I_{UC} indicate the integrated emission intensities of the reference and

the sample, with η_{ref} and η_{UC} representing the refractive indices of the solvents used for the reference and the sample, respectively. The calculation is based on the literature-known luminescence quantum yield Φ_{ref} of the reference sample [Ru(bpy)$_3$]Cl$_2$ (Φ_{ref} = 6.3% in deaerated water).[7]

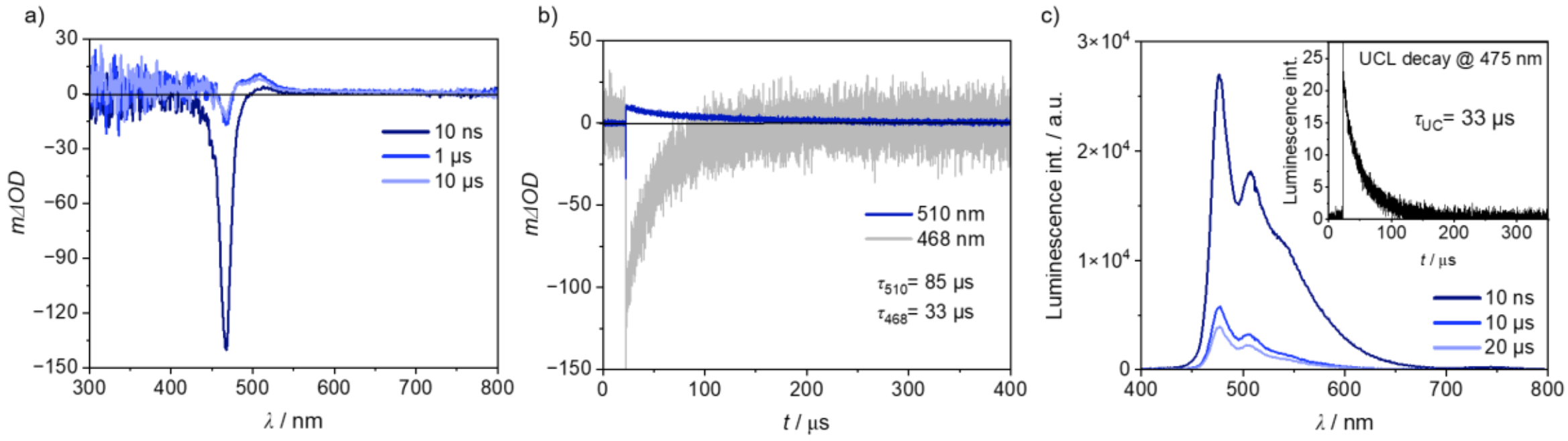

Figure S30: a) UV-Vis transient absorption spectra of Cr$_2$-An (100 µM) / AnTIPS (20 mM) in deaerated dichloroethane at 20 °C with different time delays after ns-pulsed laser excitation at 532 nm (pulse length 4 ns). b) Transient absorption decay kinetics of the sample from a) recorded at 510 nm (blue traces) and 468 nm (gray traces), and the corresponding time constants obtained from their monoexponential fit. c) Time-gated luminescence spectra of the sample from a) recorded with different time delays after ns-laser excitation at 532 nm; inset: luminescence decay kinetics at 475 nm with the monoexponentially fitted lifetime value.

The ns-TA spectra of the upconversion sample (100 µM **Cr$_2$-An** / 20 mM AnTIPS) after 532 nm excitation show two distinct features relative to those obtained without AnTIPS (Figure S23). Firstly, an excited state absorption band is detected around 510 nm, which is characteristic for the triplet state of AnTIPS.[25] Secondly, a strong negative TA signal can be observed around 470 nm. Due to the spectral position, this band arises most likely from the stimulated emission (SE) of the upconversion luminescence. Decay kinetics measurements of these TA signals show that the stimulated emission decays about twice as fast (33 µs) as the excited state absorption band (85 µs), which is the theoretical relation between the time constants for the delayed fluorescence and the triplet state of the annihilator in upconversion samples.[51] Delayed luminescence spectra of the upconversion sample under identical conditions also show the same upconversion luminescence lifetime (33 µs), confirming our assignment of the negative TA signal to the stimulated emission. These time constants depend strongly on the excitation conditions. Replacing the ns-pulsed laser with a 520 nm CW-laser with substantially longer pulse length (700 µs), the upconversion luminescence shows a substantially longer time constant of 95 µs (Figure S28d). This discrepancy arises from the different power density and pulse duration of the excitation sources, as reported by others and us. [26, 52]

10. Photocatalysis via $^1O_2$ generation

10.1 General

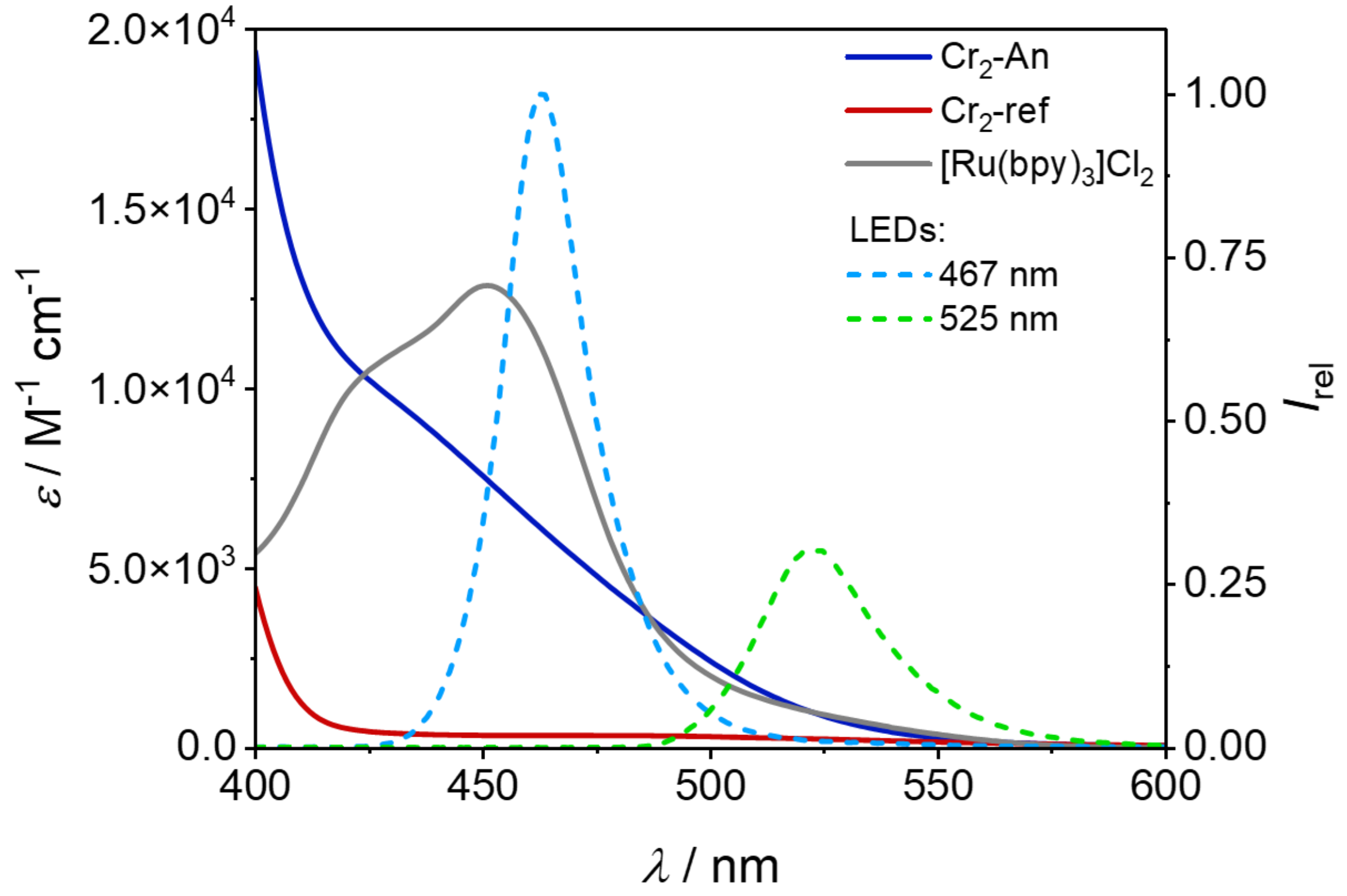

Figure S31: UV-Vis absorption spectra of **Cr_2-An** (dark blue trace), **Cr_2-ref** (red trace) and $[Ru(bpy)_3]Cl_2$ (gray trace) measured at 50 μM in acetonitrile at 20 °C. Dashed traces represent the relative emission profiles of the LEDs used in the photocatalytic experiments (467 nm, blue and 525 nm, green).

For any given photocatalytic experiments, four equivalent samples with different photocatalysts were prepared and filled in NMR tubes. The tubes were placed side by side in a beaker filled with water. A blue or green LED was fixed in front of the beaker and the whole setup was covered with aluminum foil. To follow the reaction kinetics, ^1H-NMR spectra were measured after the indicated irradiation time periods. Conversions and yields were referenced to an internal standard.

10.2 Oxidation of 1-naphthol

The substrate 1-naphthol (15 µmol, 1 eq.), the photocatalyst (1 mol% **Cr_2-An**, **Cr_2-ref** or $[Ru(bpy)_3]Cl_2$) and the internal standard hexamethylcyclotrisiloxane (2.5 µmol, ⅙ eq.) were dissolved in 0.6 mL deuterated acetonitrile and added to NMR tubes. A control sample containing both the substrate and the internal standard but no photocatalyst was prepared as well. The samples were irradiated with a Kessil PR160L 525 nm LED with a 495 nm longpass filter placed between the LED and the samples.

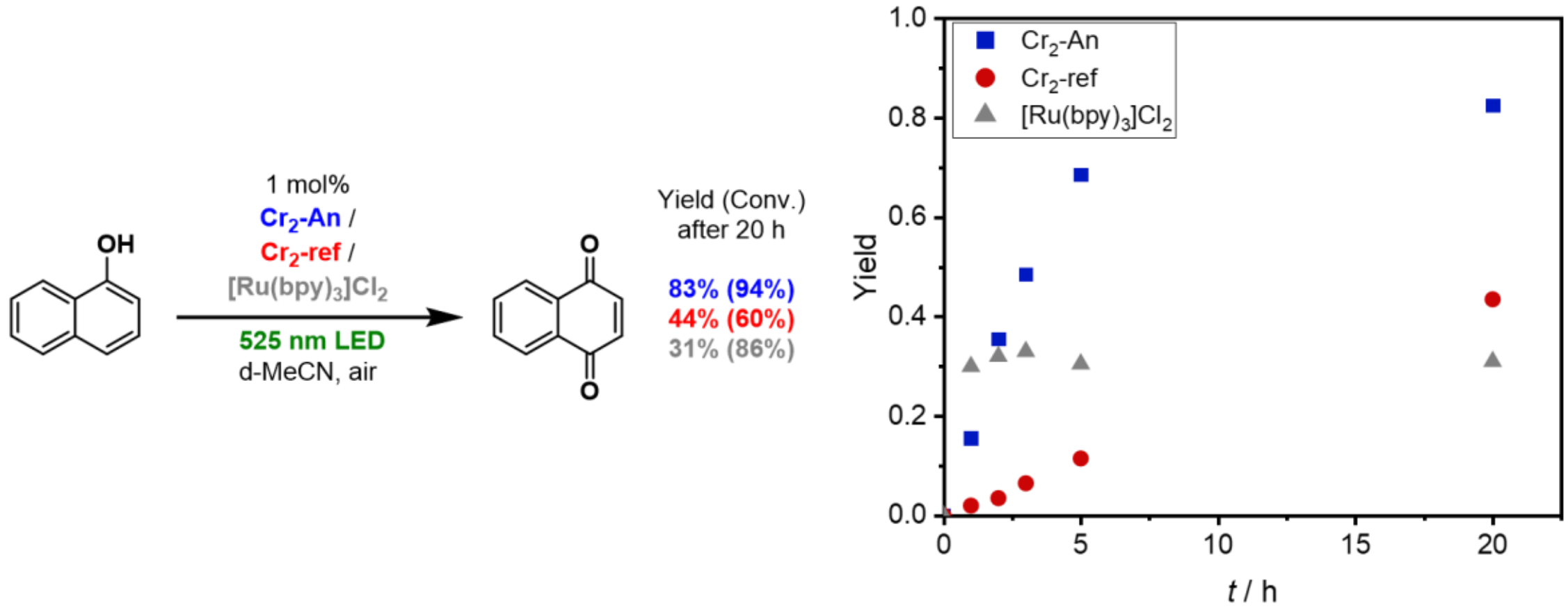

Figure S32: Reaction scheme for the oxidation of 1-naphthol and a plot of the reaction yield determined by ^{1}H-NMR spectroscopy after given irradiation time periods with **Cr_2-An** (blue squares), **Cr_2-ref** (red circles) or $[Ru(bpy)_3]Cl_2$ (gray triangles) as the photocatalyst.

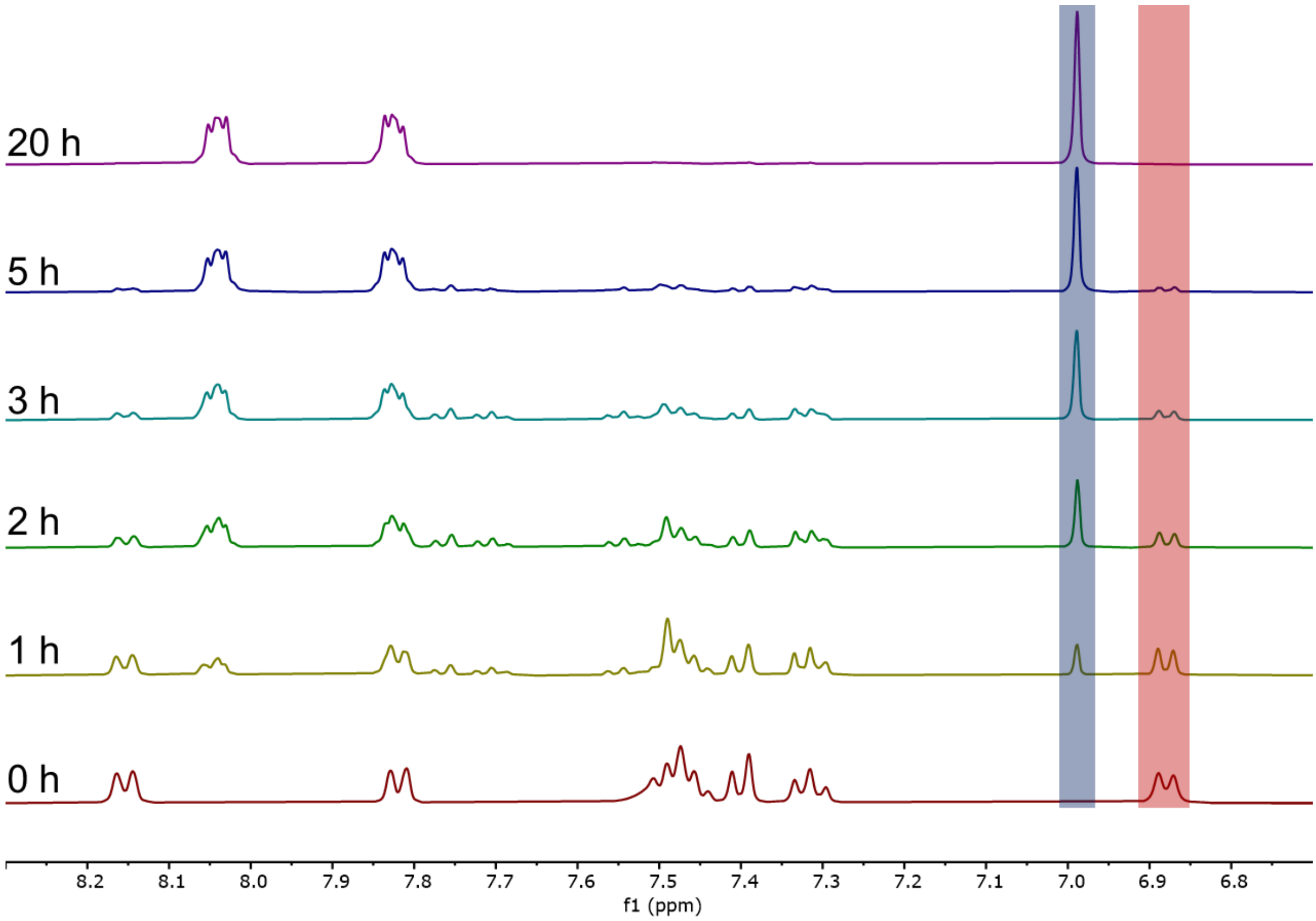

Figure S33: Excerpt of the ^{1}H-NMR spectra (400 MHz, MeCN-d_3) for the oxidation of 1-naphthol (25 mM) with **Cr₂-An** (1 mol%) as the photocatalyst and hexamethylcyclotrisiloxane (4 mM) as an internal standard. The sample was irradiated with a 525 nm LED for the indicated time. Conversion and yield were determined by integrating the highlighted signals corresponding to the substrate (red) and the product (blue) and referencing them to the signal of the internal standard at 0.14 ppm.

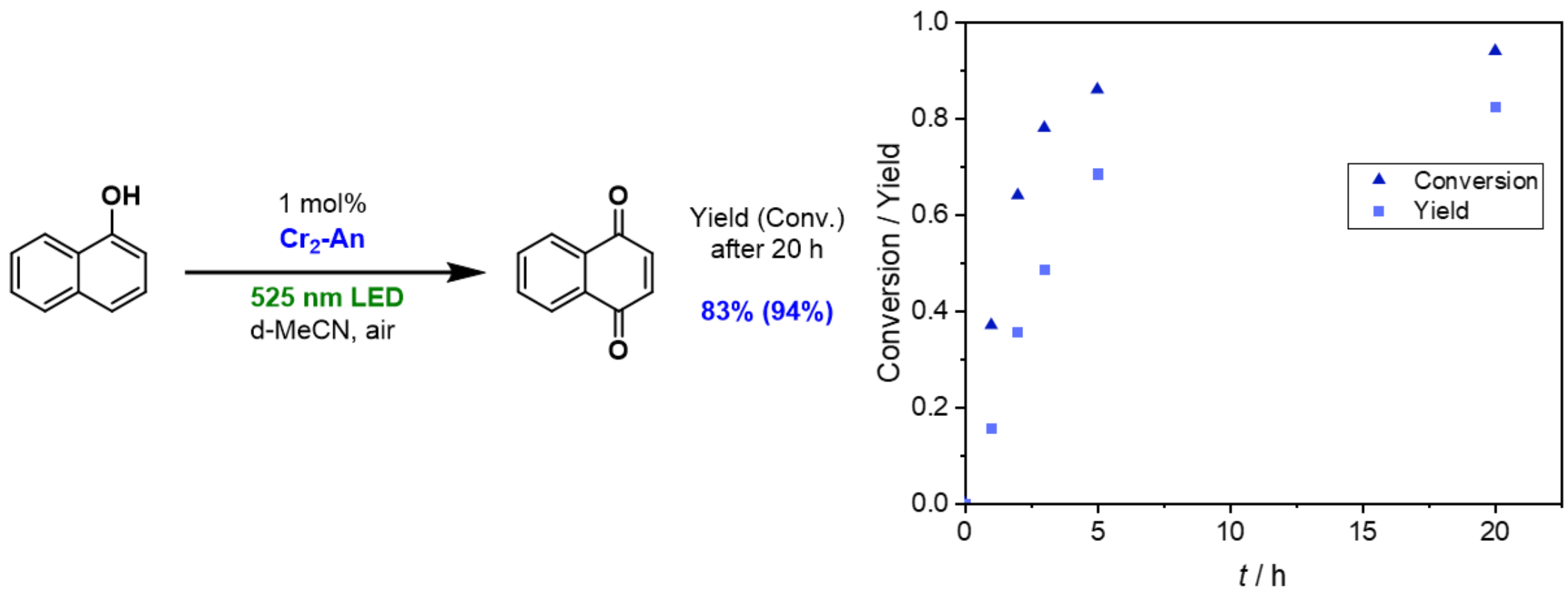

Figure S34: Reaction scheme for the oxidation of 1-naphthol and a plot of the conversion (dark blue triangles) and reaction yield (light blue squares) determined by ^{1}H-NMR spectroscopy after given irradiation time periods with **Cr₂-An** as the photocatalyst.

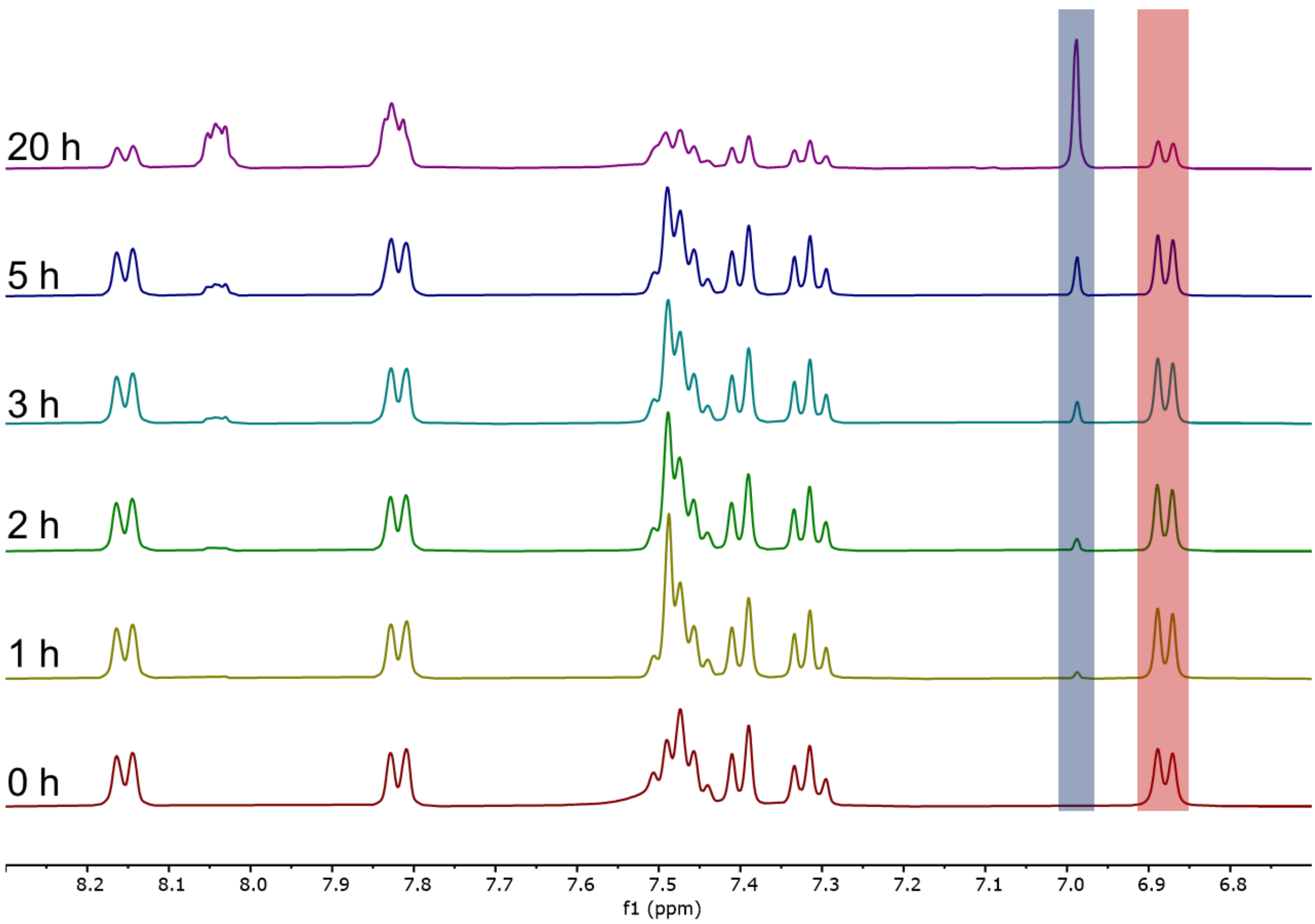

Figure S35: Excerpt of the ^{1}H-NMR spectra (400 MHz, MeCN-d_3) for the oxidation of 1-naphthol (25 mM) with **Cr_2-ref** (1 mol%) as the photocatalyst and hexamethylcyclotrisiloxane (4 mM) as an internal standard. The sample was irradiated with a 525 nm LED for the indicated time. Conversion and yield were determined by integrating the highlighted signals corresponding to the substrate (red) and the product (blue) and referencing them to the signal of the internal standard at 0.14 ppm.

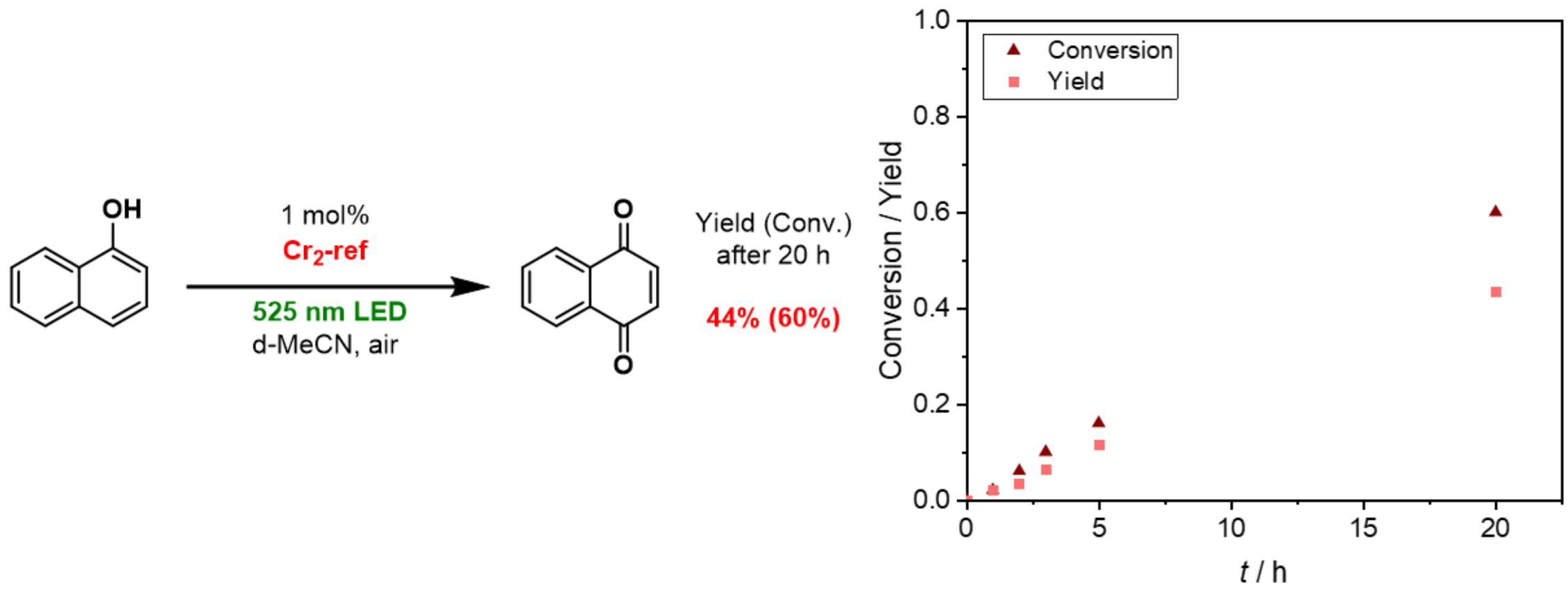

Figure S36: Reaction scheme for the oxidation of 1-naphthol and a plot of the conversion (dark red triangles) and reaction yield (light red squares) determined by ^{1}H-NMR spectroscopy after given irradiation time periods with **Cr_2-ref** as the photocatalyst.

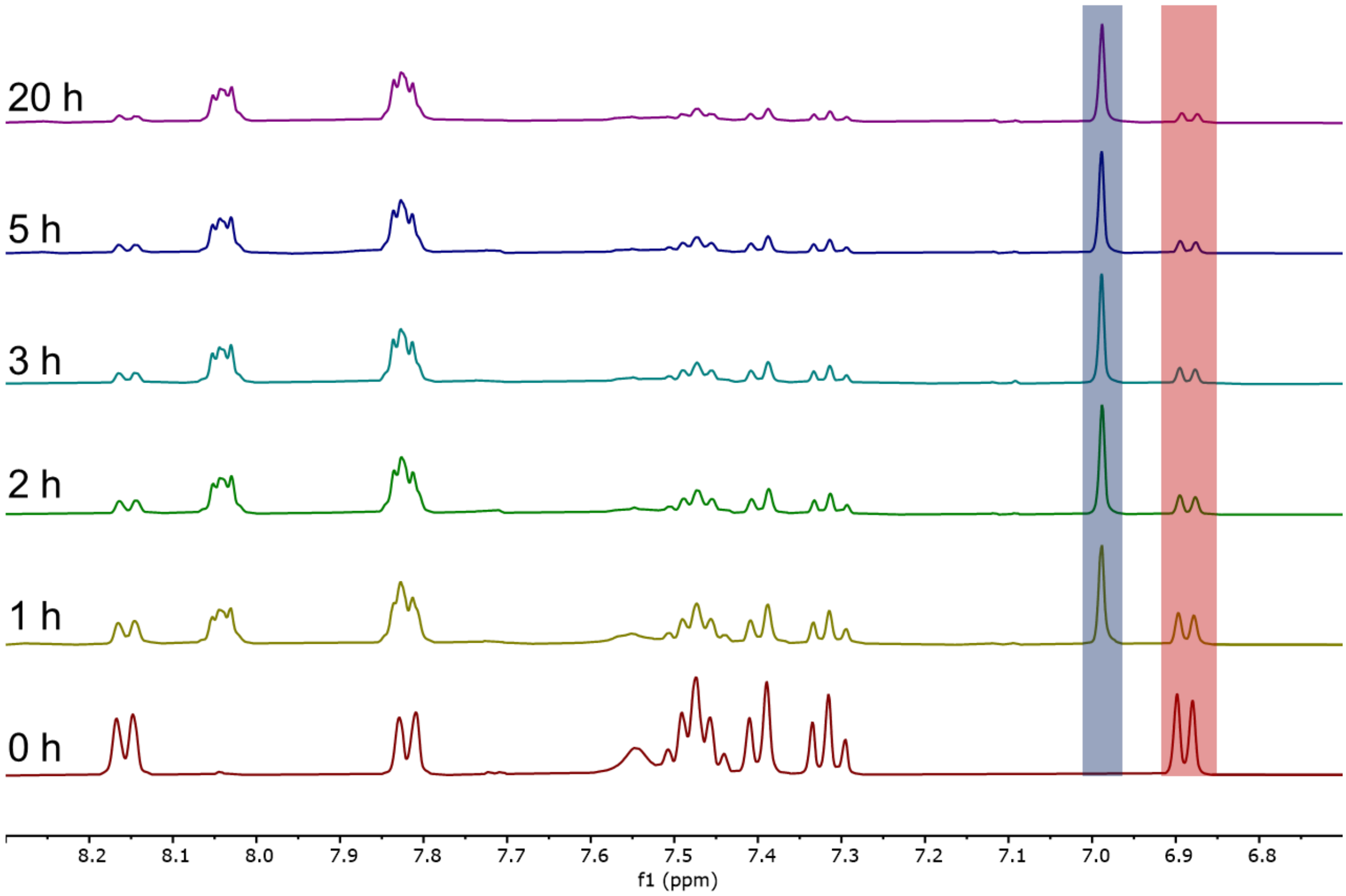

Figure S37: Excerpt of the ^{1}H-NMR spectra (400 MHz, MeCN-d_3) for the oxidation of 1-naphthol (25 mM) with $[Ru(bpy)_3]Cl_2$ (1 mol%) as the photocatalyst and hexamethylcyclotrisiloxane (4 mM) as an internal standard. The sample was irradiated with a 525 nm LED for the indicated time. Conversion and yield were determined by integrating the highlighted signals corresponding to the substrate (red) and the product (blue) and referencing them to the signal of the internal standard at 0.14 ppm.

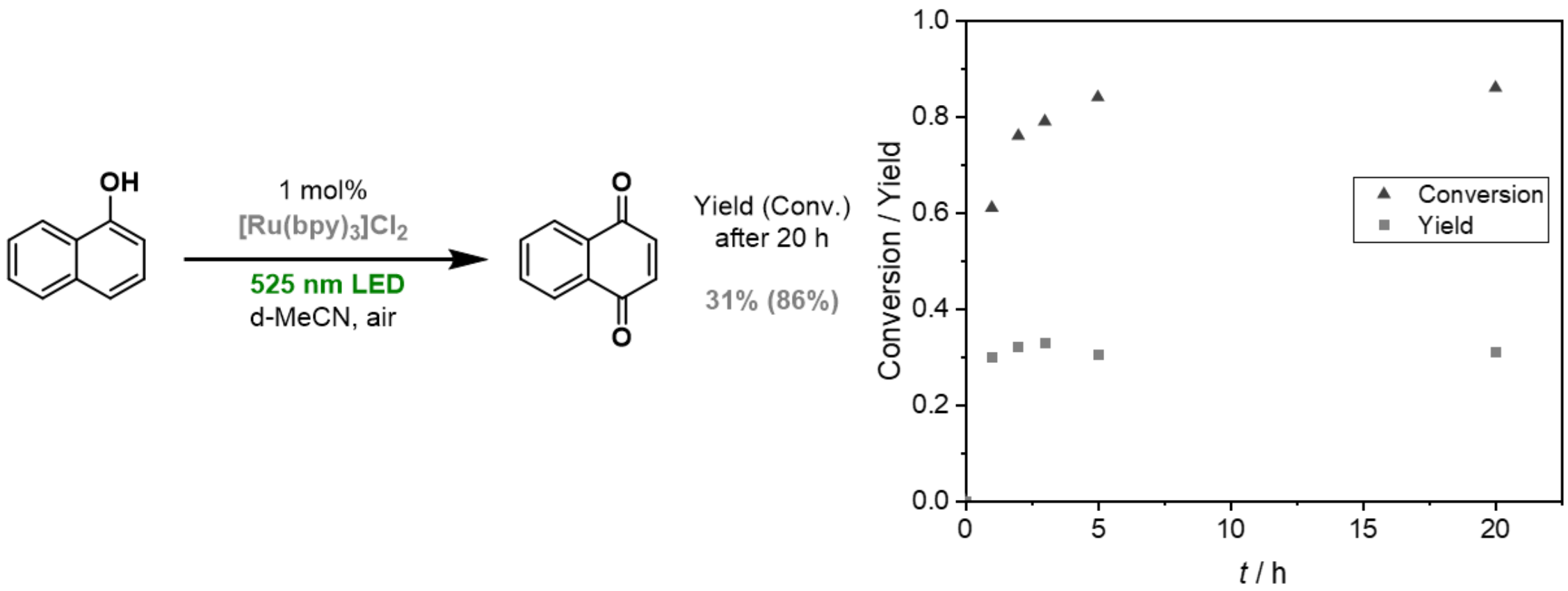

Figure S38: Reaction scheme for the oxidation of 1-naphthol and a plot of the conversion (dark gray triangles) and reaction yield (light gray squares) determined by ^{1}H-NMR spectroscopy after given irradiation time periods with $[Ru(bpy)_3]Cl_2$ as the photocatalyst.

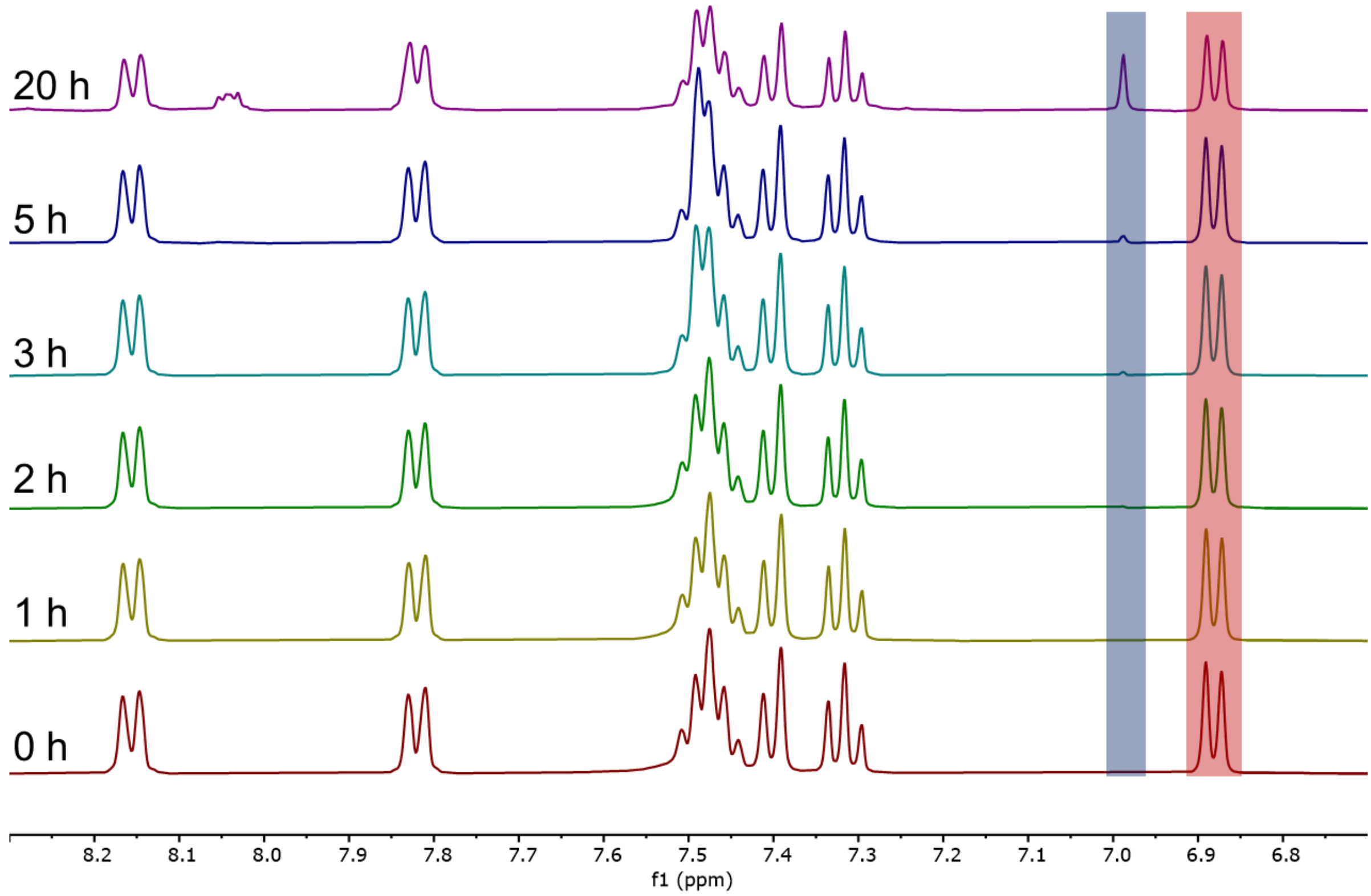

Figure S39: Excerpt of the ^{1}H-NMR spectra (400 MHz, MeCN-d_3) for the control reaction of 1-naphthol (25 mM) without a photocatalyst and hexamethylcyclotrisiloxane (4 mM) as an internal standard. The sample was irradiated with a 525 nm LED for the indicated time. Conversion and yield were determined by integrating the highlighted signals corresponding to the substrate (red) and the product (blue) and referencing them to the signal of the internal standard at 0.14 ppm.

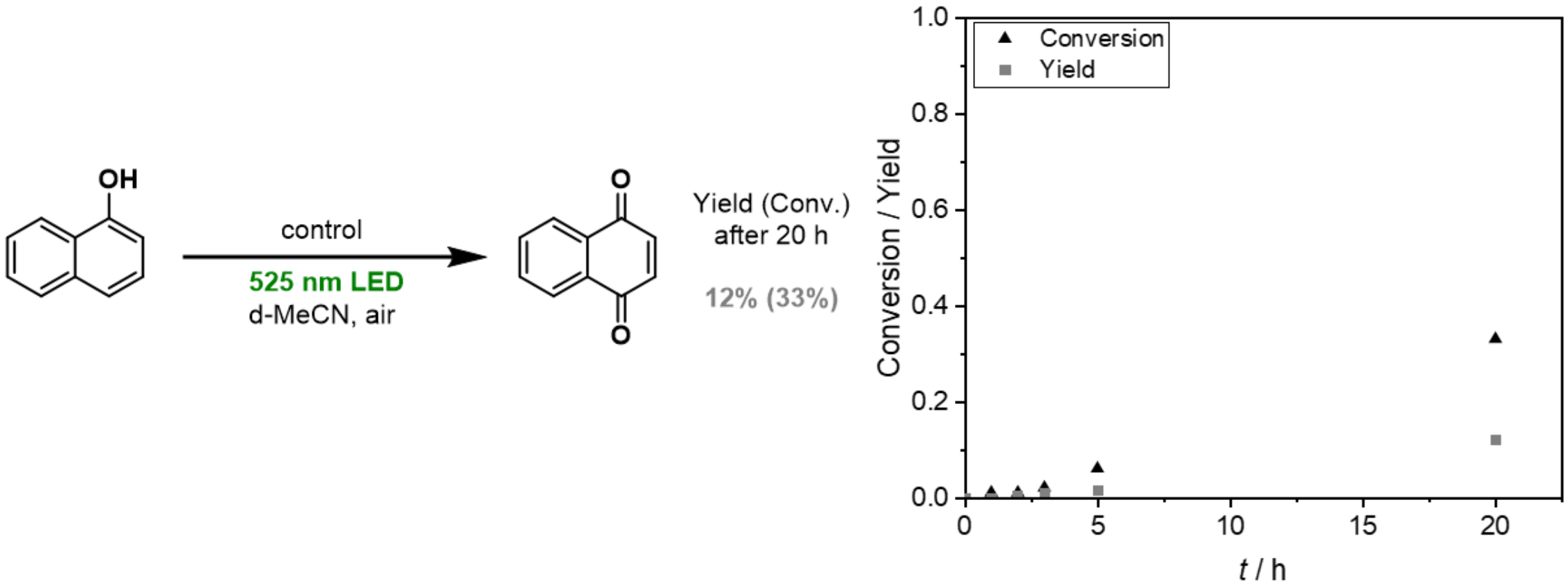

Figure S40: Reaction scheme for the control reaction of 1-naphthol without a photocatalyst and a plot of the conversion (black triangles) and reaction yield (gray squares) determined by ^{1}H-NMR spectroscopy after given irradiation time periods.

For the control reaction of 1-naphthol oxidation in the absence of any photocatalyst, minimal amounts of product formation (1.5% after 5 h, 12% after 20 h) were observed without any photocatalyst present. This might be due to marginal light absorption of the substrate itself or any impurities present in the reaction mixture. This effect was deemed insignificant for the catalyzed reactions as under these conditions, the vast majority of light was absorbed by the photocatalysts.

10.3 Oxidation of α-terpinene

The substrate α-terpinene (15 µmol, 1 eq.), the photocatalyst (1 mol% **Cr_2-An**, **Cr_2-ref** or $[Ru(bpy)_3]Cl_2$) and the internal standard hexamethylcyclotrisiloxane (2.5 µmol, ⅙ eq.) were dissolved in 0.6 mL deuterated acetonitrile and added to NMR tubes. A control sample containing both the substrate and the internal standard but no photocatalyst was prepared as well. The samples were irradiated with a Kessil PR160L 467 nm LED with a 435 nm longpass filter placed between the LED and the samples.

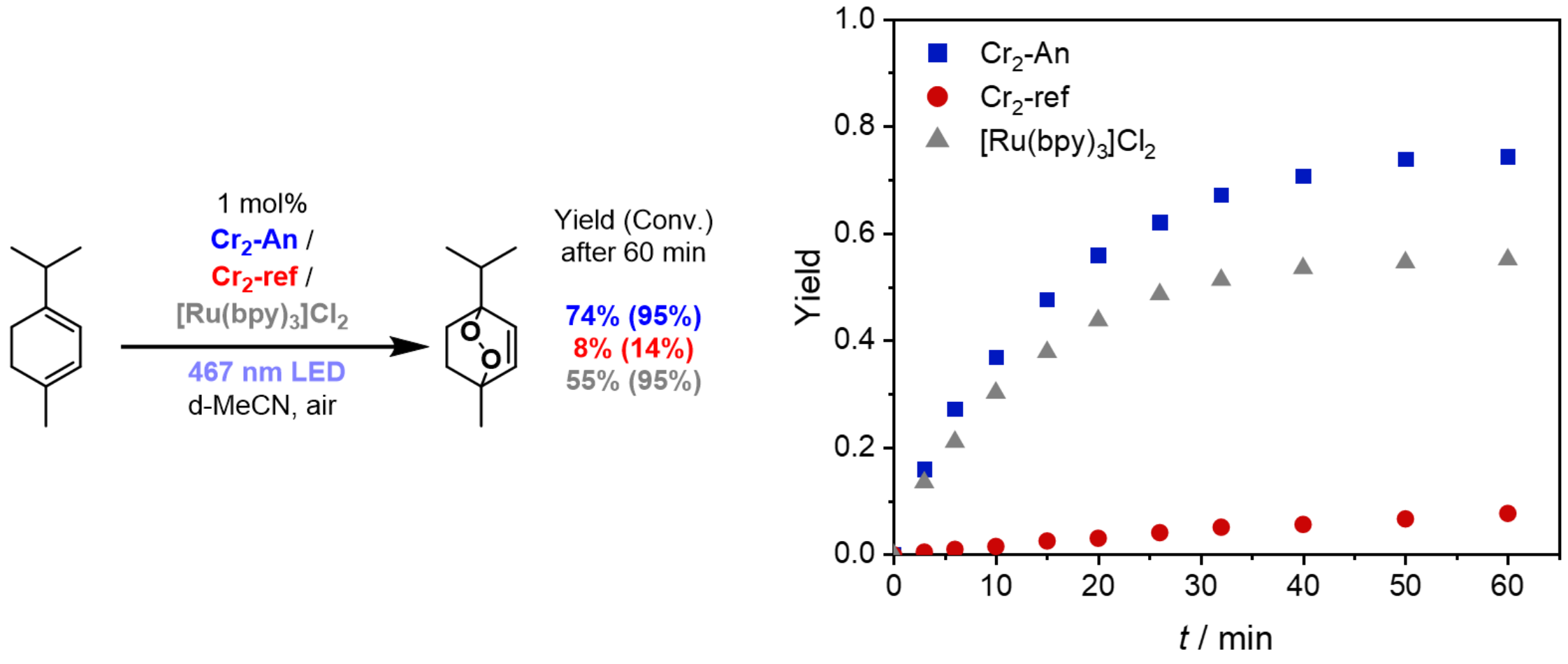

Figure S41: Reaction scheme for the oxidation of α-terpinene and a plot of the reaction yield determined by ^{1}H-NMR spectroscopy after given irradiation time periods with **Cr_2-An** (blue squares), **Cr_2-ref** (red circles) or $[Ru(bpy)_3]Cl_2$ (gray triangles) as the photocatalyst.

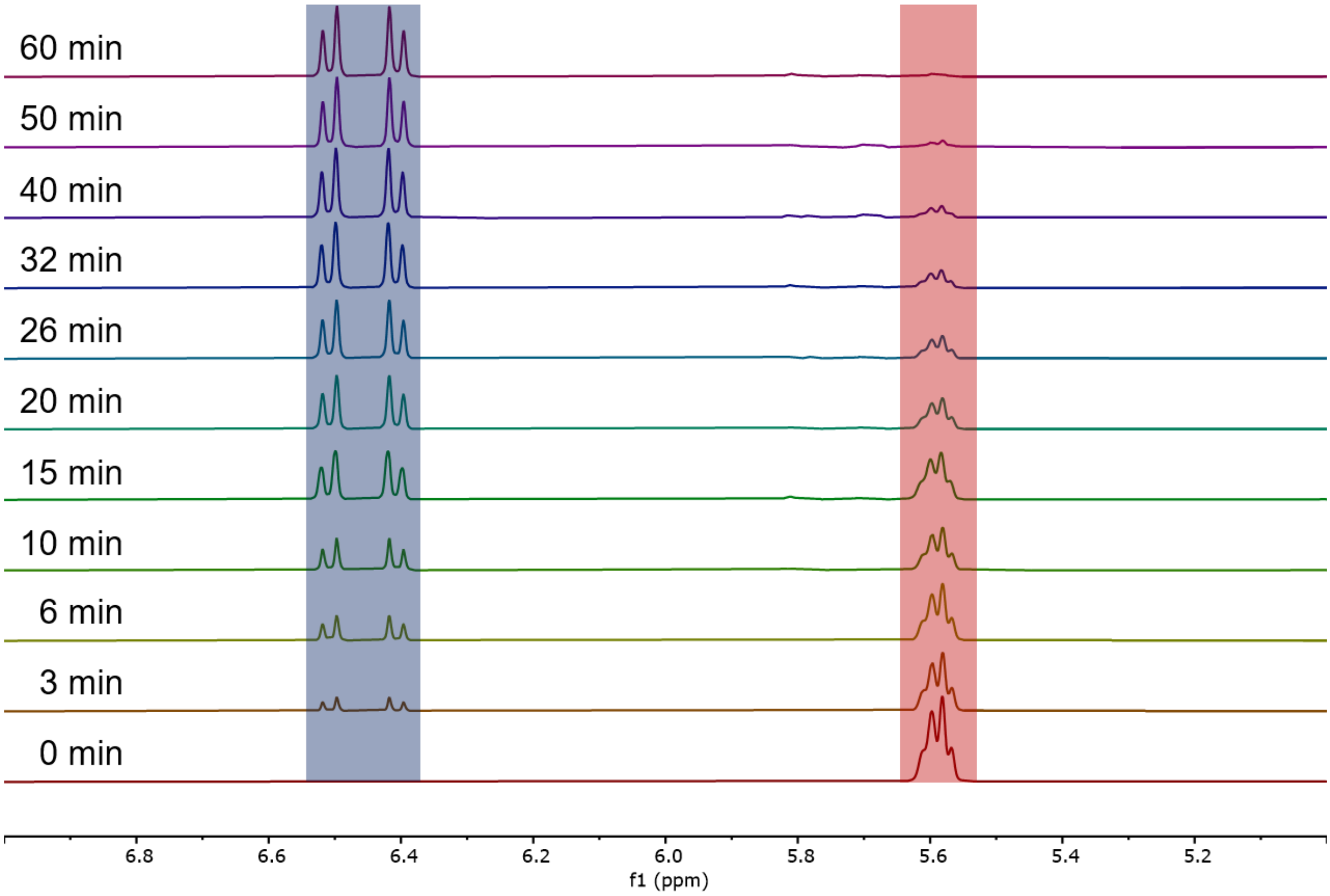

Figure S42: Excerpt of the ^{1}H-NMR spectra (400 MHz, MeCN-d_3) for the oxidation of α-terpinene (25 mM) with **Cr_2-An** (1 mol%) as the photocatalyst and hexamethylcyclotrisiloxane (4 mM) as an internal standard. The sample was irradiated with a 467 nm LED for the indicated time. Conversion and yield were determined by integrating the highlighted signals corresponding to the substrate (red) and the product (blue) and referencing them to the signal of the internal standard at 0.14 ppm.

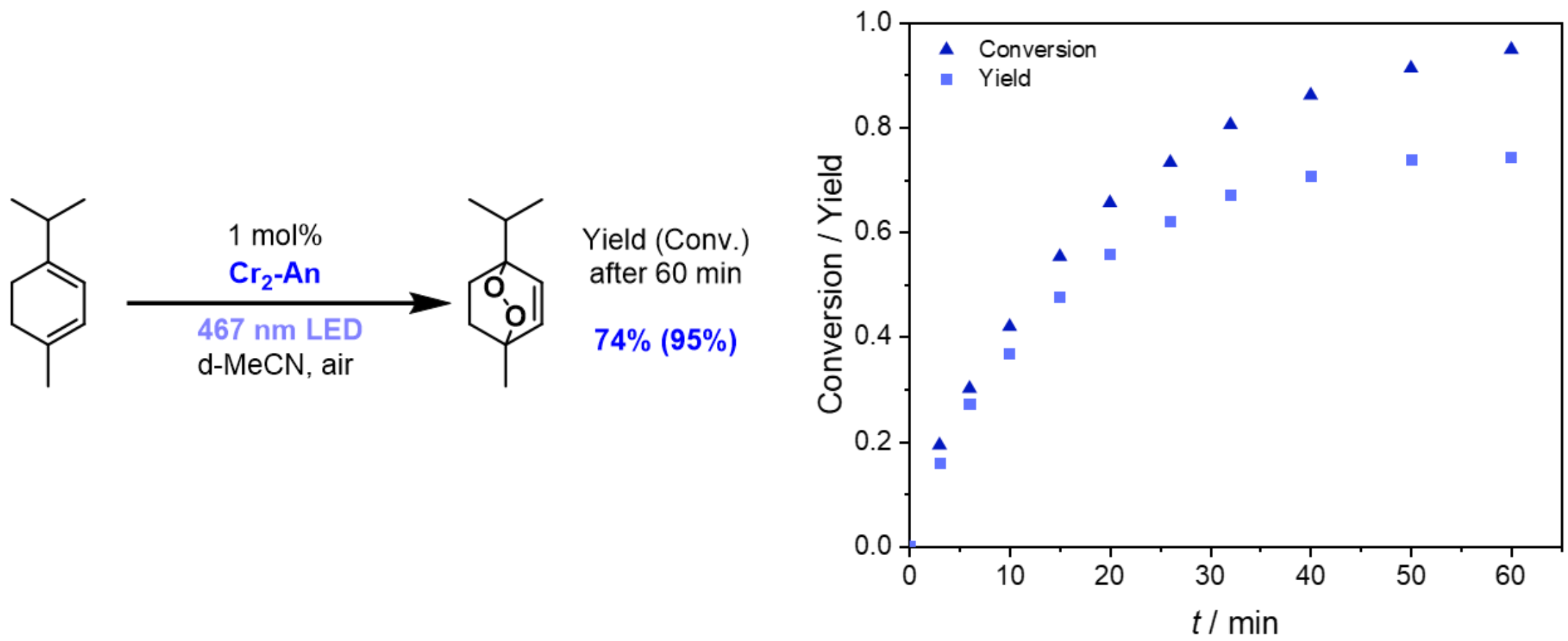

Figure S43: Reaction scheme for the oxidation of α-terpinene and a plot of the conversion (dark blue triangles) and reaction yield (light blue squares) determined by ^{1}H-NMR spectroscopy after given irradiation time periods with **Cr_2-An** as the photocatalyst.

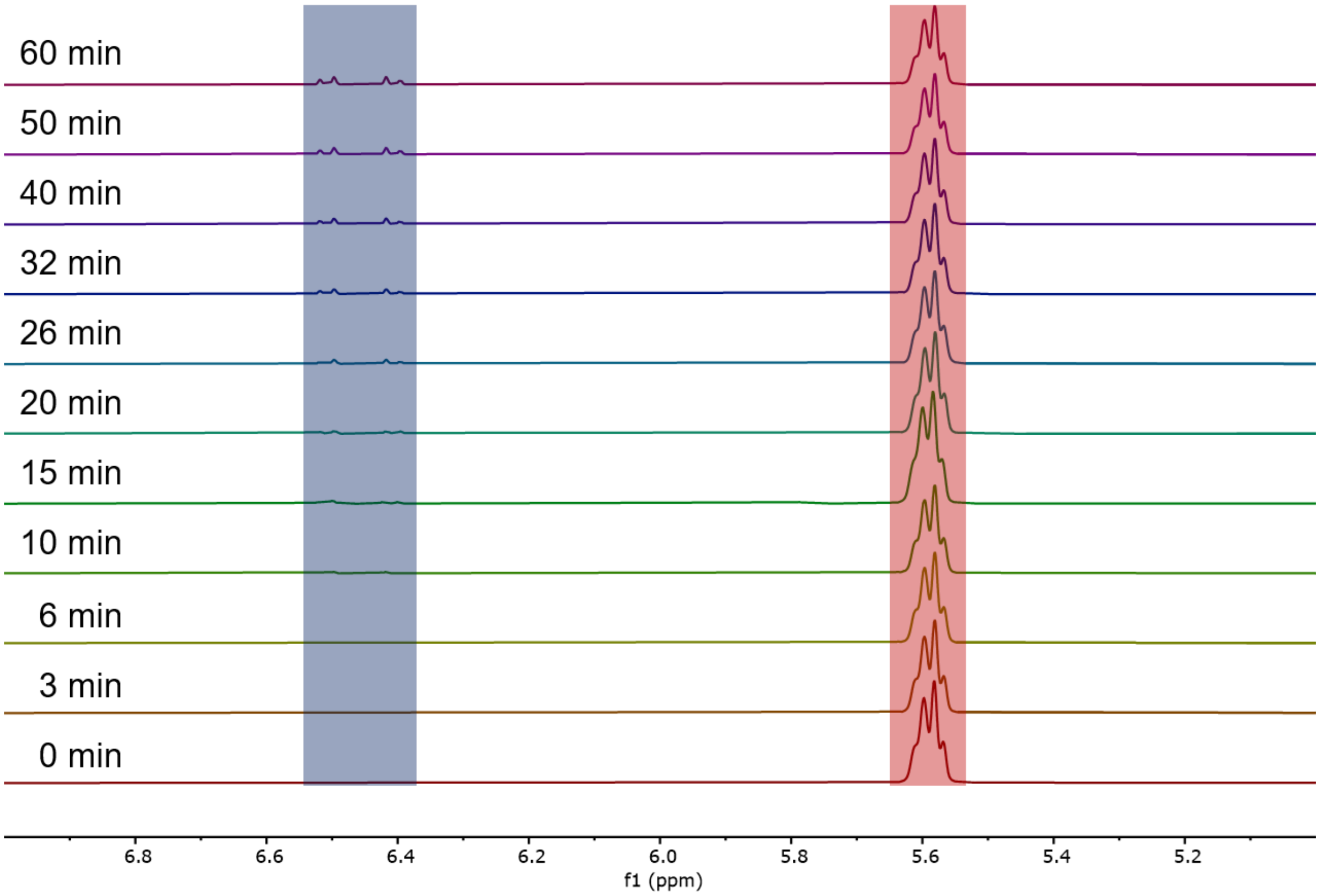

Figure S44: Excerpt of the ^{1}H-NMR spectra (400 MHz, MeCN-d_3) for the oxidation of α-terpinene (25 mM) with **Cr_2-ref** (1 mol%) as the photocatalyst and hexamethylcyclotrisiloxane (4 mM) as an internal standard. The sample was irradiated with a 467 nm LED for the indicated time. Conversion and yield were determined by integrating the highlighted signals corresponding to the substrate (red) and the product (blue) and referencing them to the signal of the internal standard at 0.14 ppm.

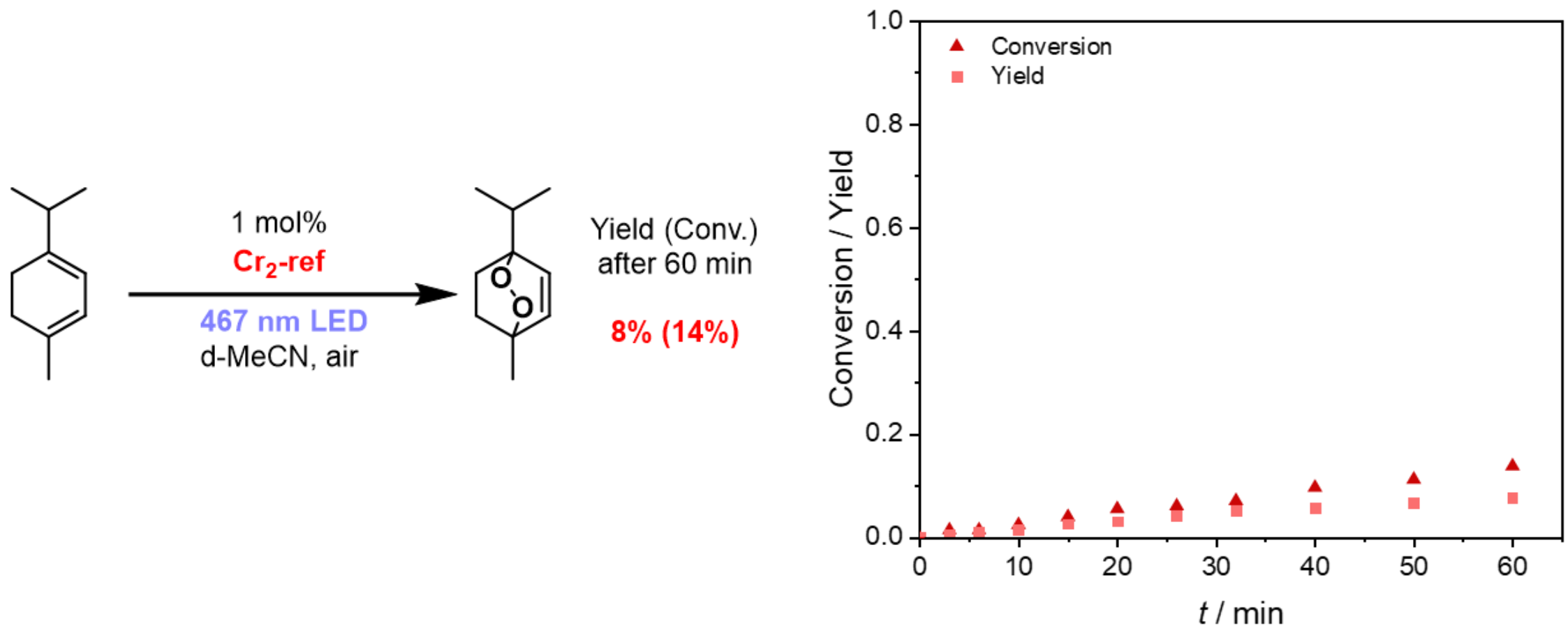

Figure S45: Reaction scheme for the oxidation of α-terpinene and a plot of the conversion (dark red triangles) and reaction yield (light red squares) determined by ^{1}H-NMR spectroscopy after given irradiation time periods with **Cr_2-ref** as the photocatalyst.

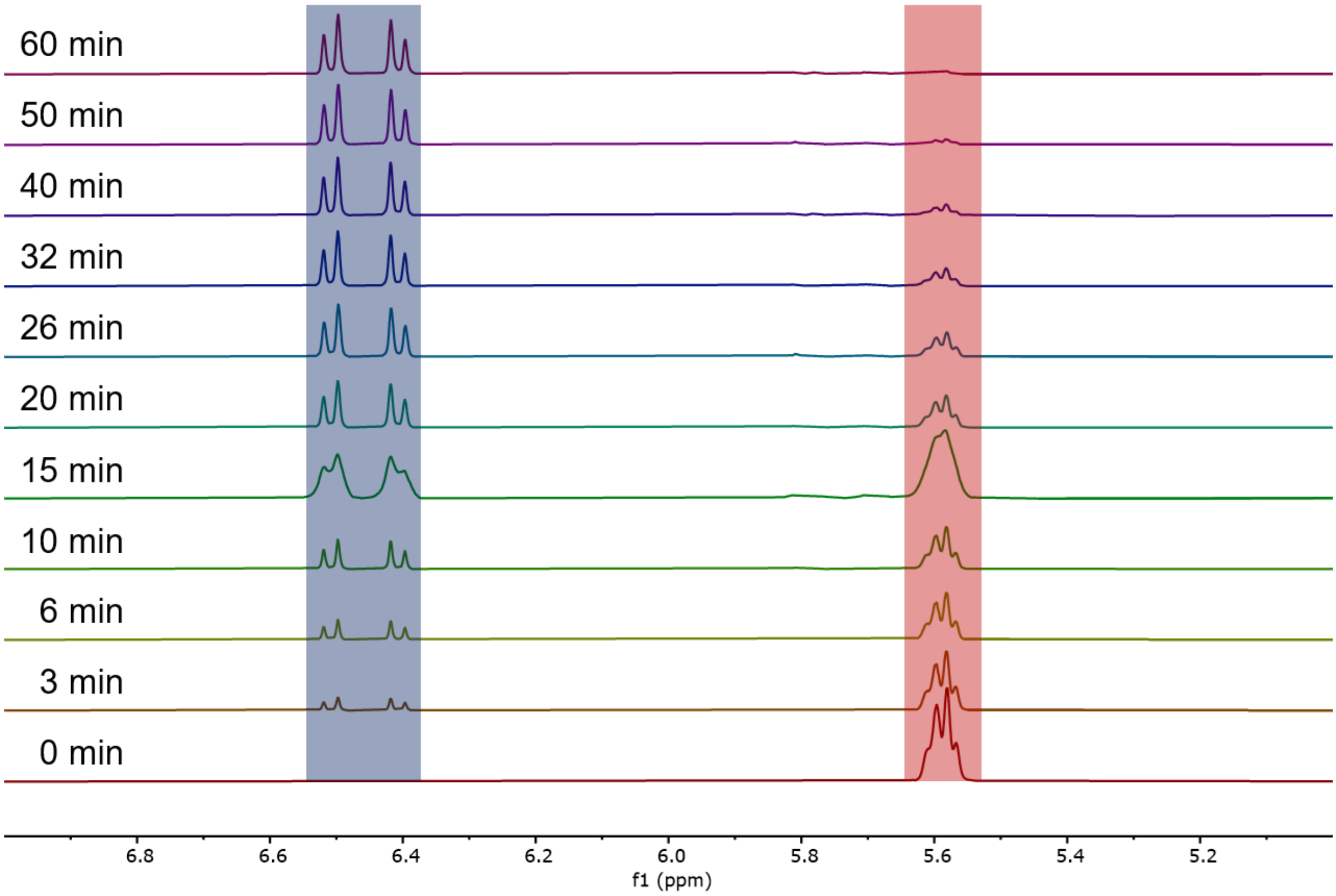

Figure S46: Excerpt of the ^{1}H-NMR spectra (400 MHz, MeCN-d_3) for the oxidation of α-terpinene (25 mM) with $[Ru(bpy)_3]Cl_2$ (1 mol%) as the photocatalyst and hexamethylcyclotrisiloxane (4 mM) as an internal standard. The sample was irradiated with a 467 nm LED for the indicated time. Conversion and yield were determined by integrating the highlighted signals corresponding to the substrate (red) and the product (blue) and referencing them to the signal of the internal standard at 0.14 ppm.

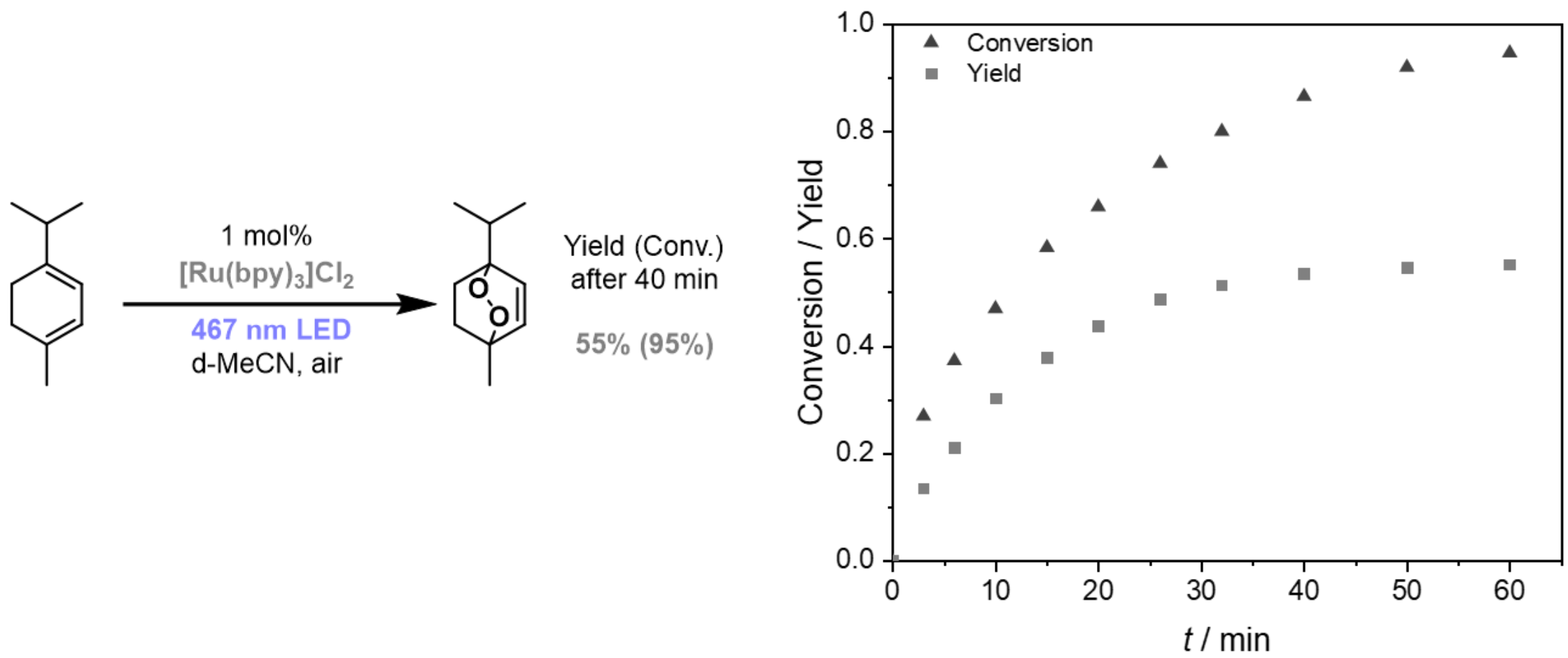

Figure S47: Reaction scheme for the oxidation of α-terpinene and a plot of the conversion (dark gray triangles) and reaction yield (light gray squares) determined by ^{1}H-NMR spectroscopy after given irradiation time periods with $[Ru(bpy)_3]Cl_2$ as the photocatalyst.

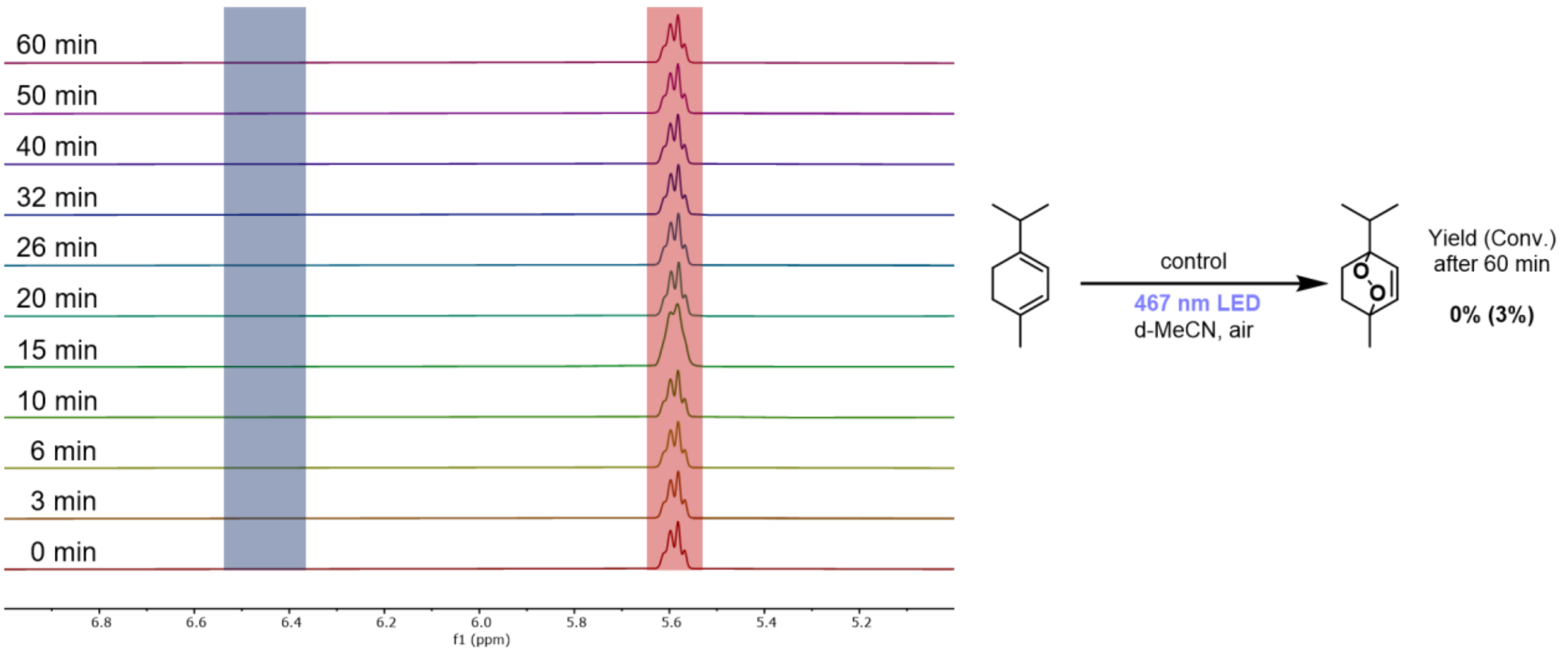

Figure S48: Excerpt of the ^{1}H-NMR spectra (400 MHz, MeCN-d_3) and the corresponding reaction scheme for the control reaction of α-terpinene (25 mM) without a photocatalyst and hexamethylcyclotrisiloxane (4 mM) as an internal standard. The sample was irradiated with a 467 nm LED for the indicated time. Conversion and yield were determined by integrating the highlighted signals corresponding to the substrate (red) and the product (blue) and referencing them to the signal of the internal standard at 0.14 ppm.

10.4 Direct quenching of photocatalysts by substrates

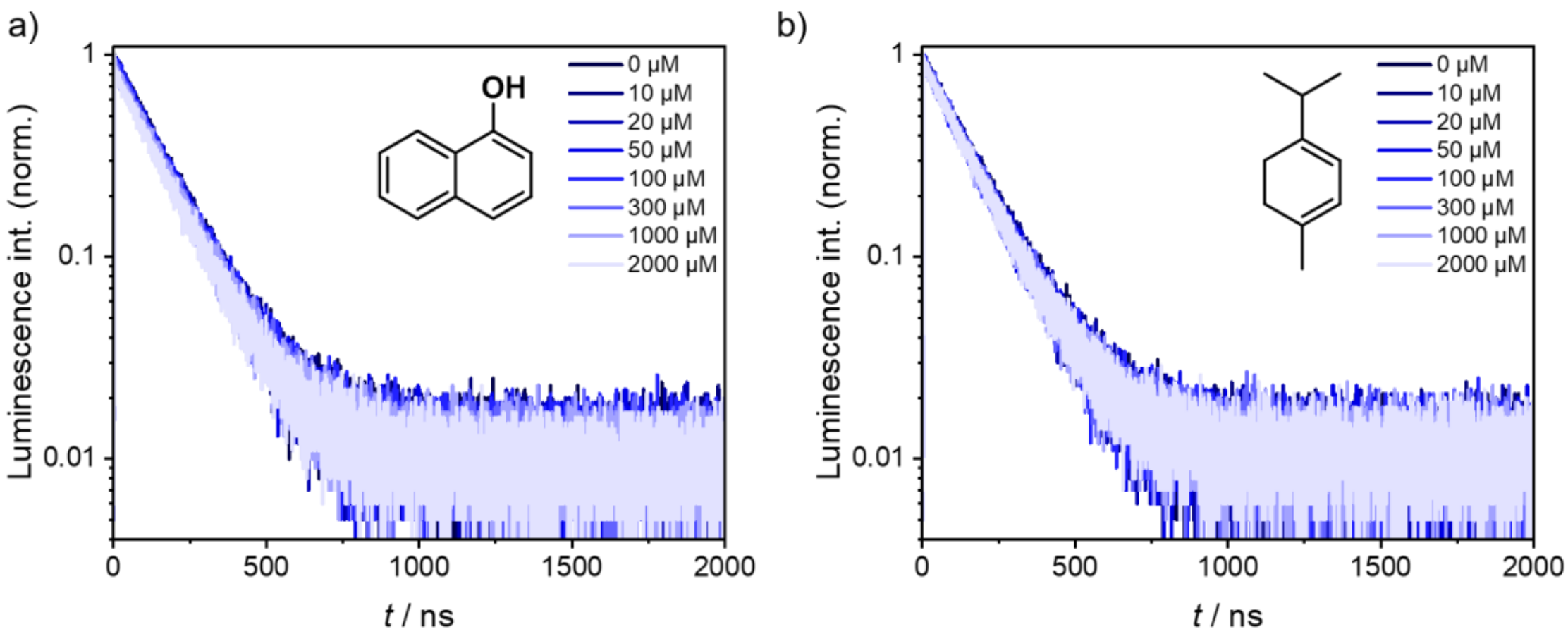

Figure S49: Luminescence lifetime measurements of **Cr_2-An** (50 µM) in deaerated acetonitrile at 20 °C with increasing concentrations of 1-naphthol (a) or α-terpinene (b). The samples were excited at 450 nm and the luminescence was detected at 745 nm.

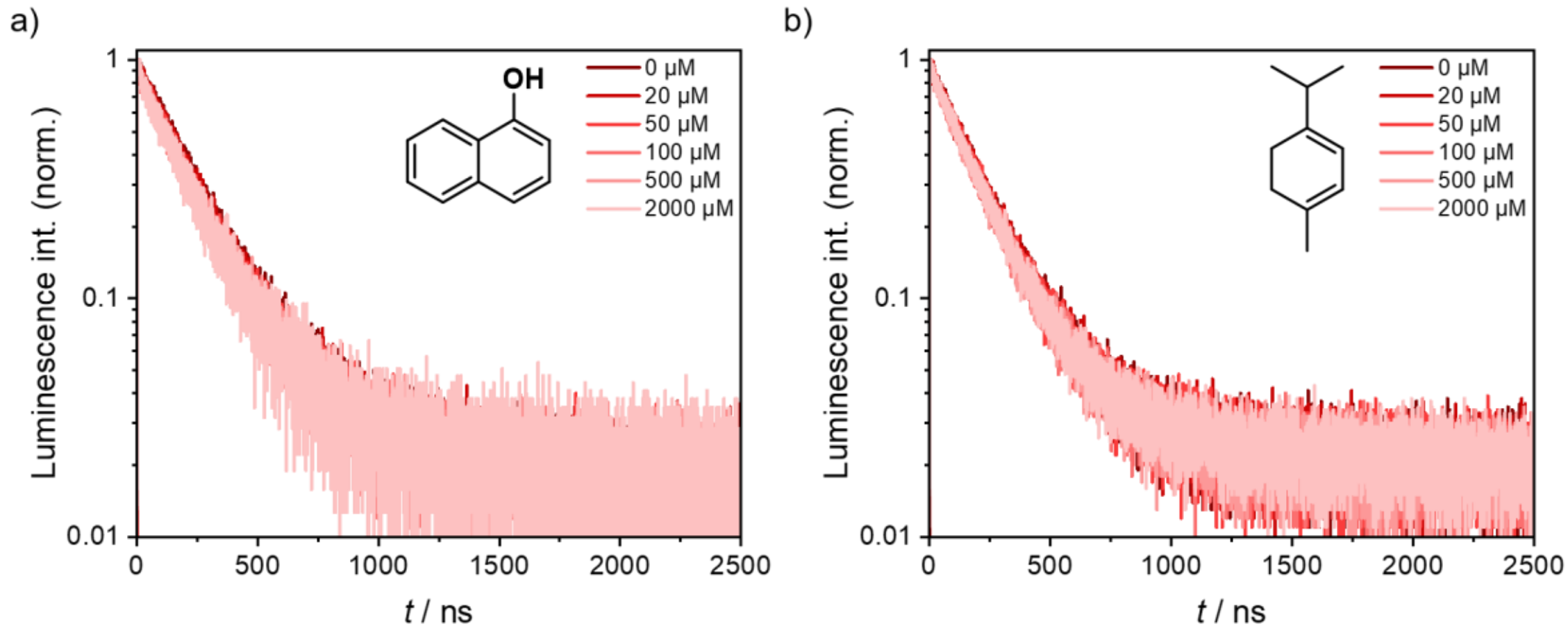

Figure S50: Luminescence lifetime measurements of **Cr_2-ref** (50 µM) in deaerated acetonitrile at 20 °C with increasing concentrations of 1-naphthol (a) or α-terpinene (b). The samples were excited at 375 nm and the luminescence was detected at 726 nm.

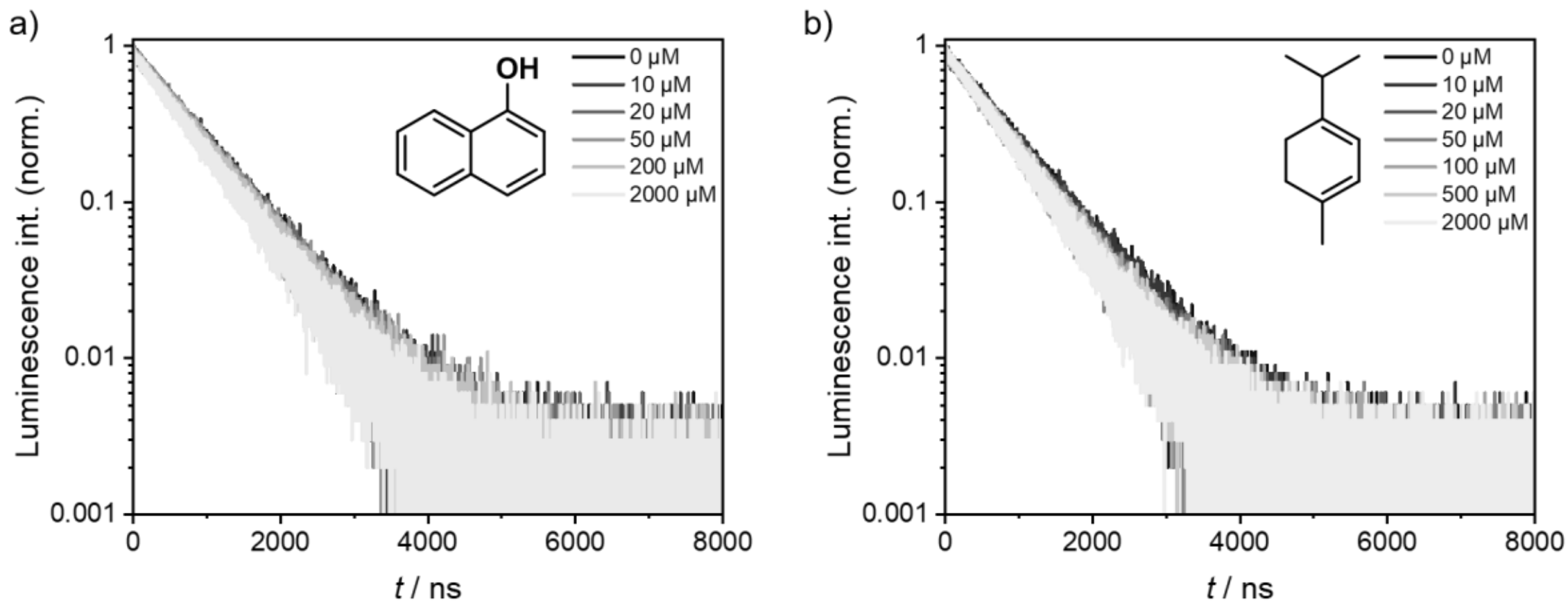

Figure S51: Luminescence lifetime measurements of $[Ru(bpy)_3]Cl_2$ (50 µM) in deaerated acetonitrile at 20 °C with increasing concentrations of 1-naphthol (a) or α-terpinene (b). The samples were excited at 450 nm and the luminescence was detected at 615 nm.

Based on the excited state energies (Table 1, main paper) and the redox potentials for both Cr(III) dimers (Figure S4), the excited state reduction potentials are estimated to be 0.83 V and 0.78 V vs SCE for **Cr_2-An** and **Cr_2-ref** respectively. This is similar to the known value for $[Ru(bpy)_3]^{2+}$ (0.77 V vs SCE).[53] With the oxidation potentials of 1-naphthol and α-terpinene being 1.35 V and 1.2 V vs SCE,[54, 55] photoinduced electron transfer reactions would be endergonic by 0.37-0.58 eV and therefore thermodynamically not feasible. This is further evidenced by the luminescence quenching experiments shown above, in which no obvious quenching is observed for our Cr(III) dimers or the Ru(II) complex upon addition of the substrates (Figures S49 - S51). This suggests that photoinduced electron transfer does not play an observable role under our conditions. However, the formed 1,4-naphthoquinone from the oxidation of 1-naphthol is a known electron acceptor.[56] This might undergo oxidative quenching with excited $[Ru(bpy)_3]^{2+}$, which could reduce the yield of the initially formed desired product and account for the large discrepancy between the conversion and the yield.

11. NMR spectra

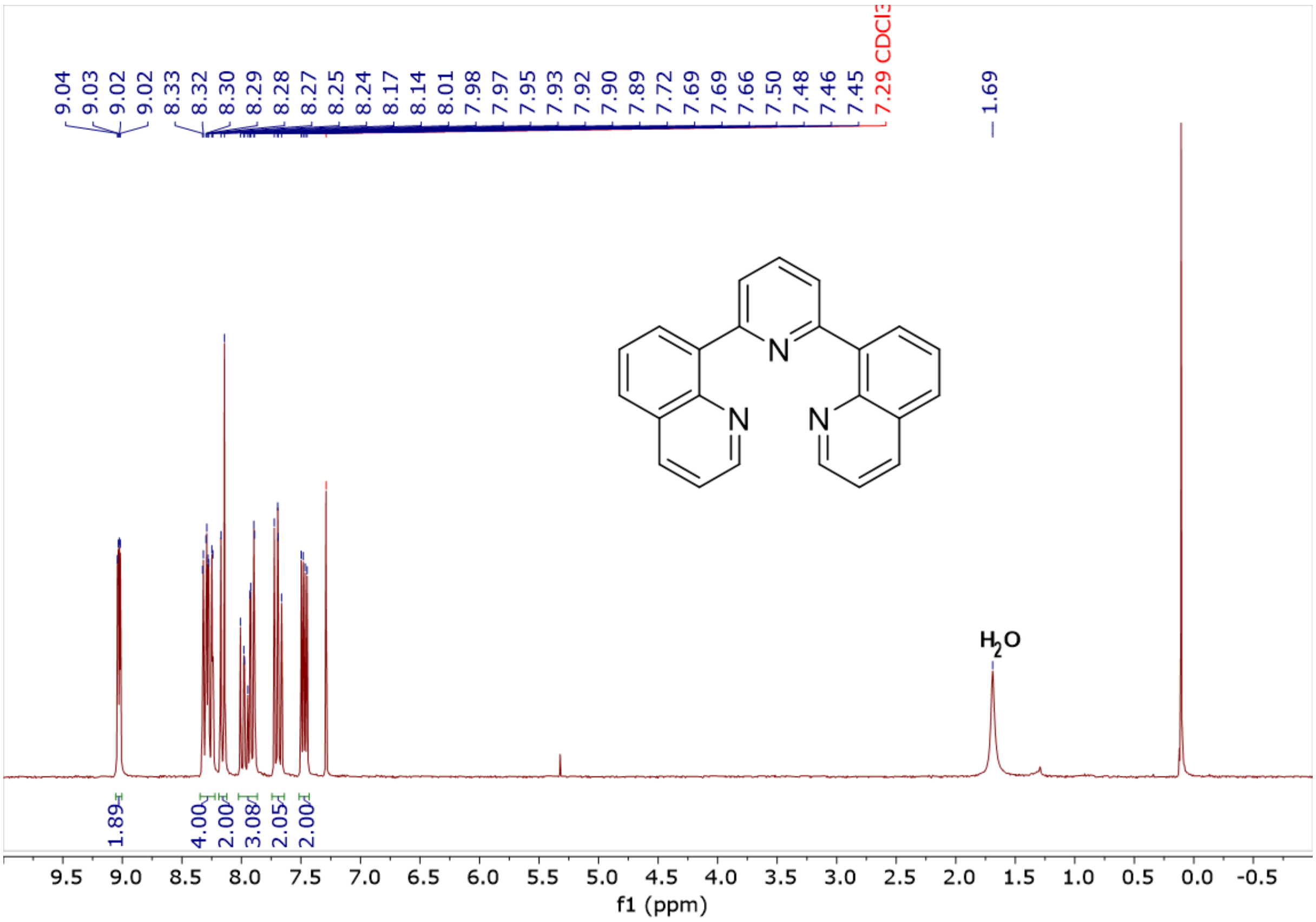

Figure S52: ^{1}H-NMR spectrum of dqp in $CDCl_3$.

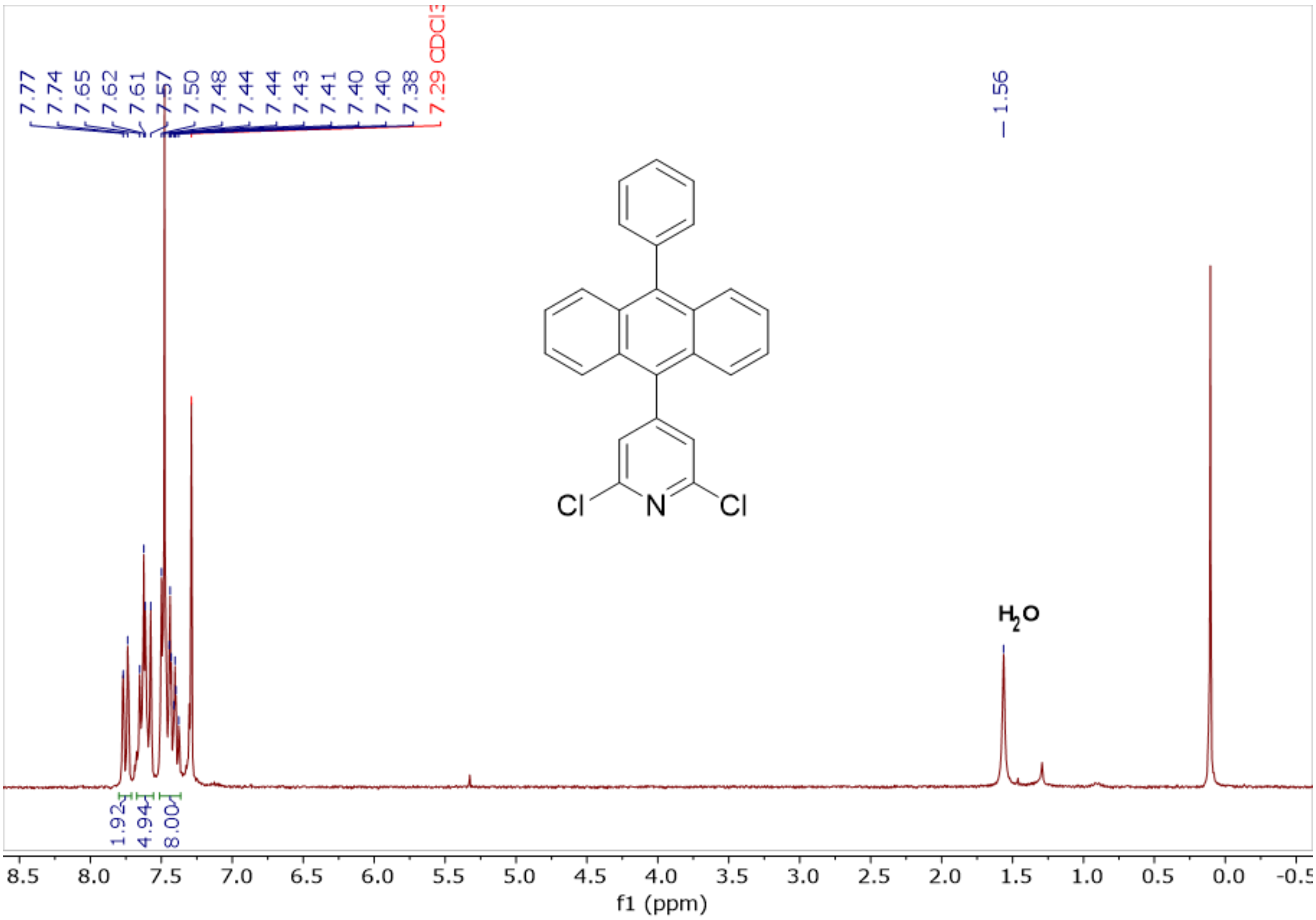

Figure S53: ^{1}H-NMR spectrum of Py-PA in $CDCl_3$.

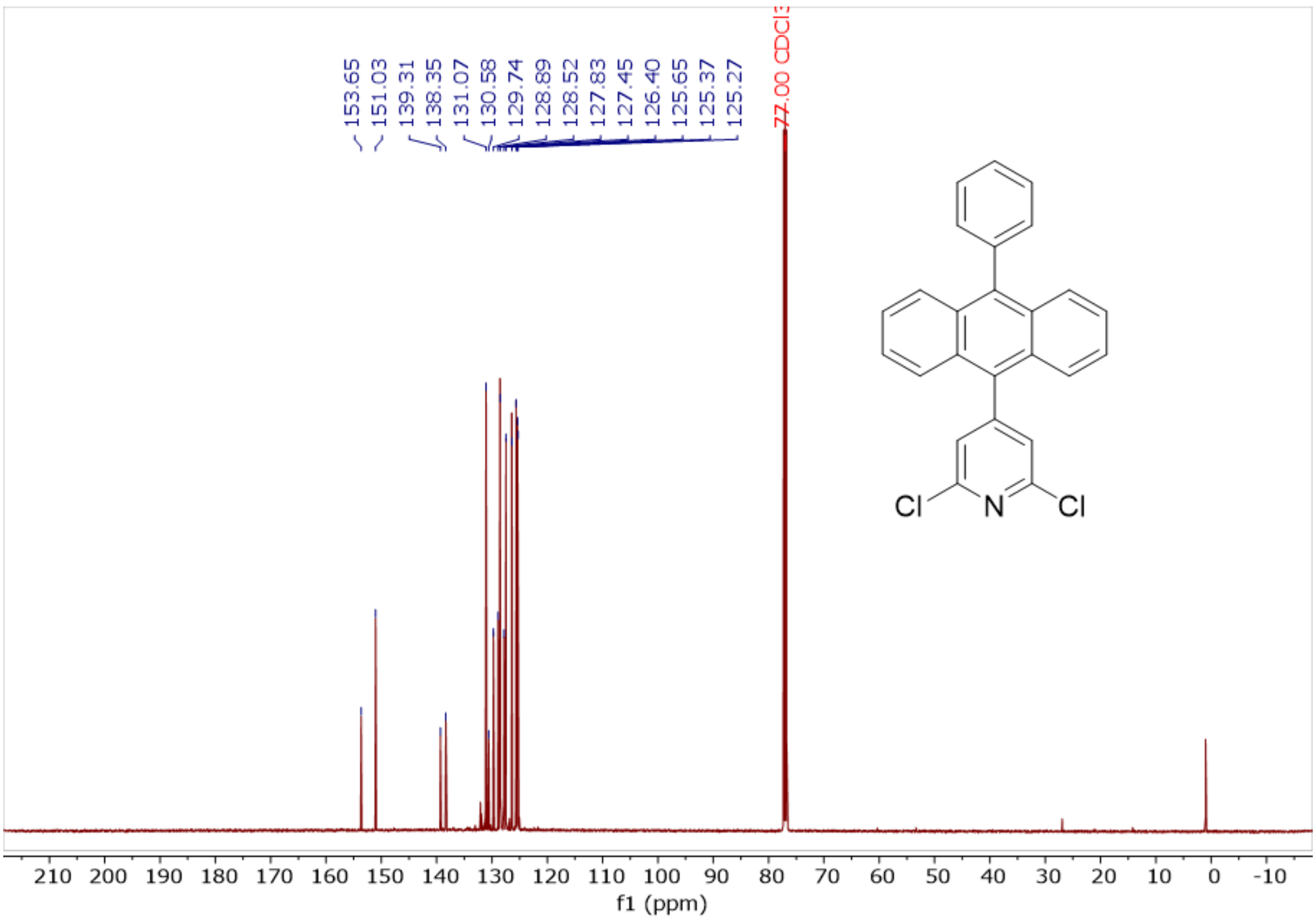

Figure S54: ^{13}C-NMR spectrum of Py-PA in $CDCl_3$

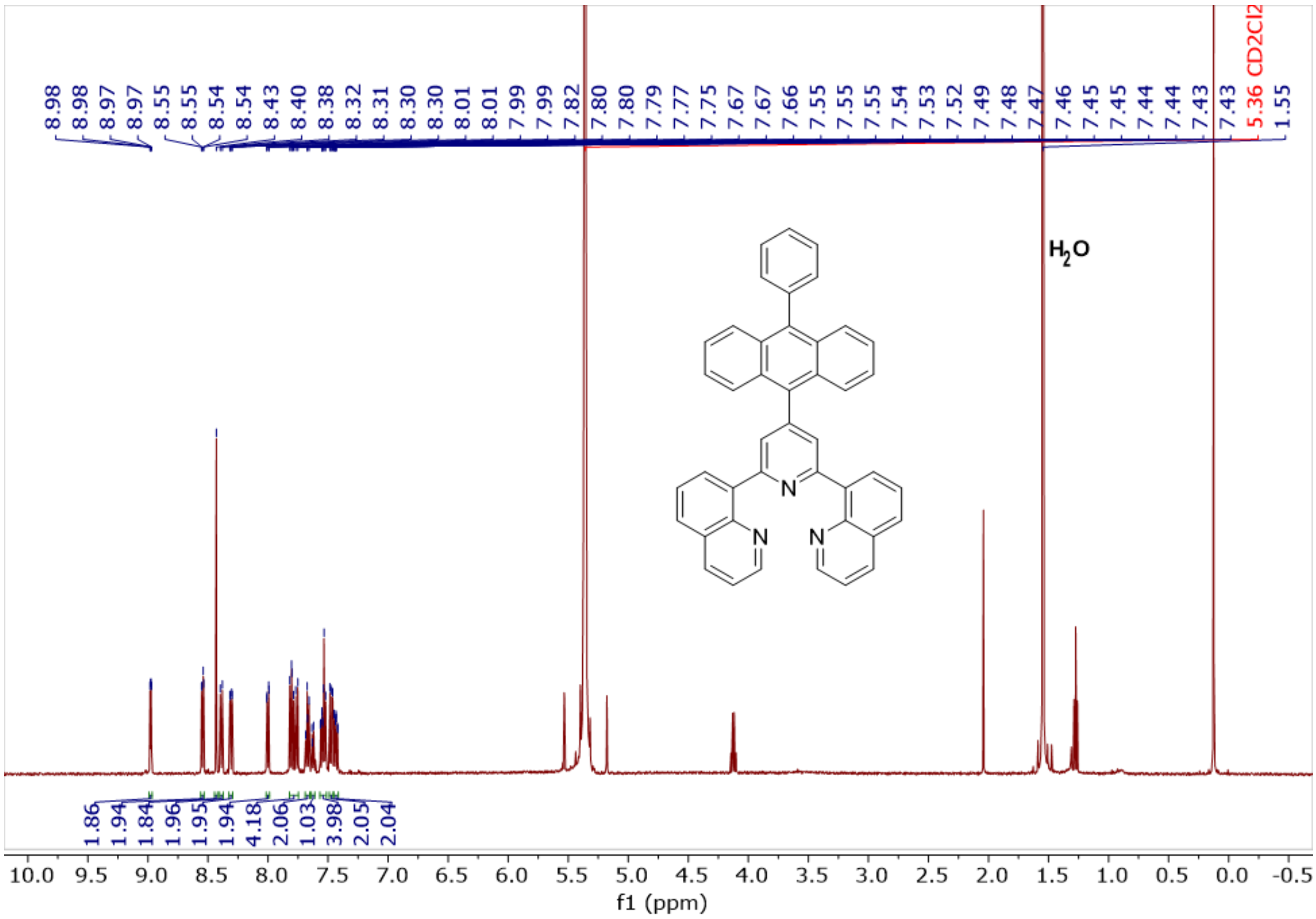

Figure S55: ^{1}H-NMR spectrum of dqp-PA in CD_2Cl_2.

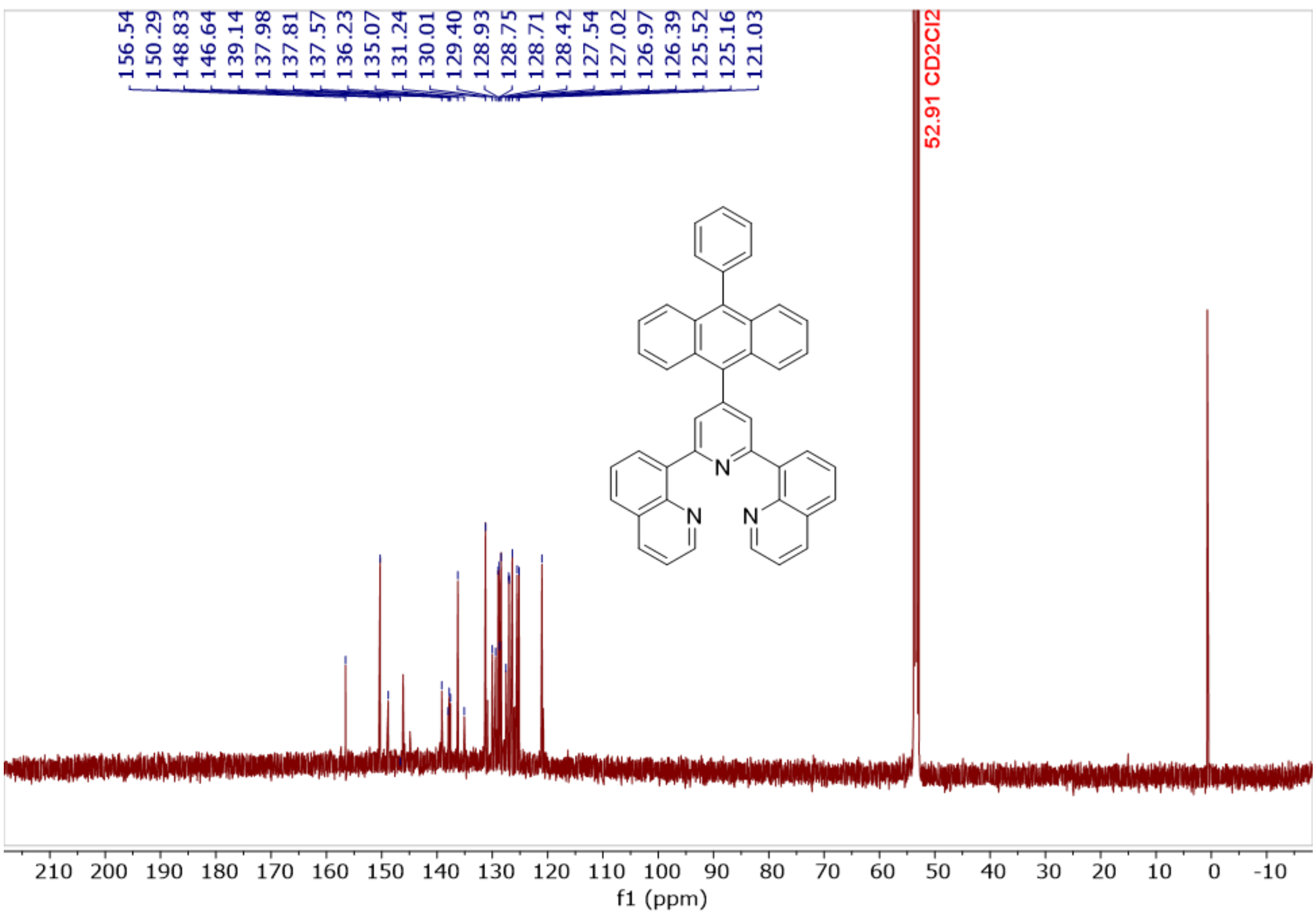

Figure S56: ^{13}C-NMR spectrum of dqp-PA in CD_2Cl_2.

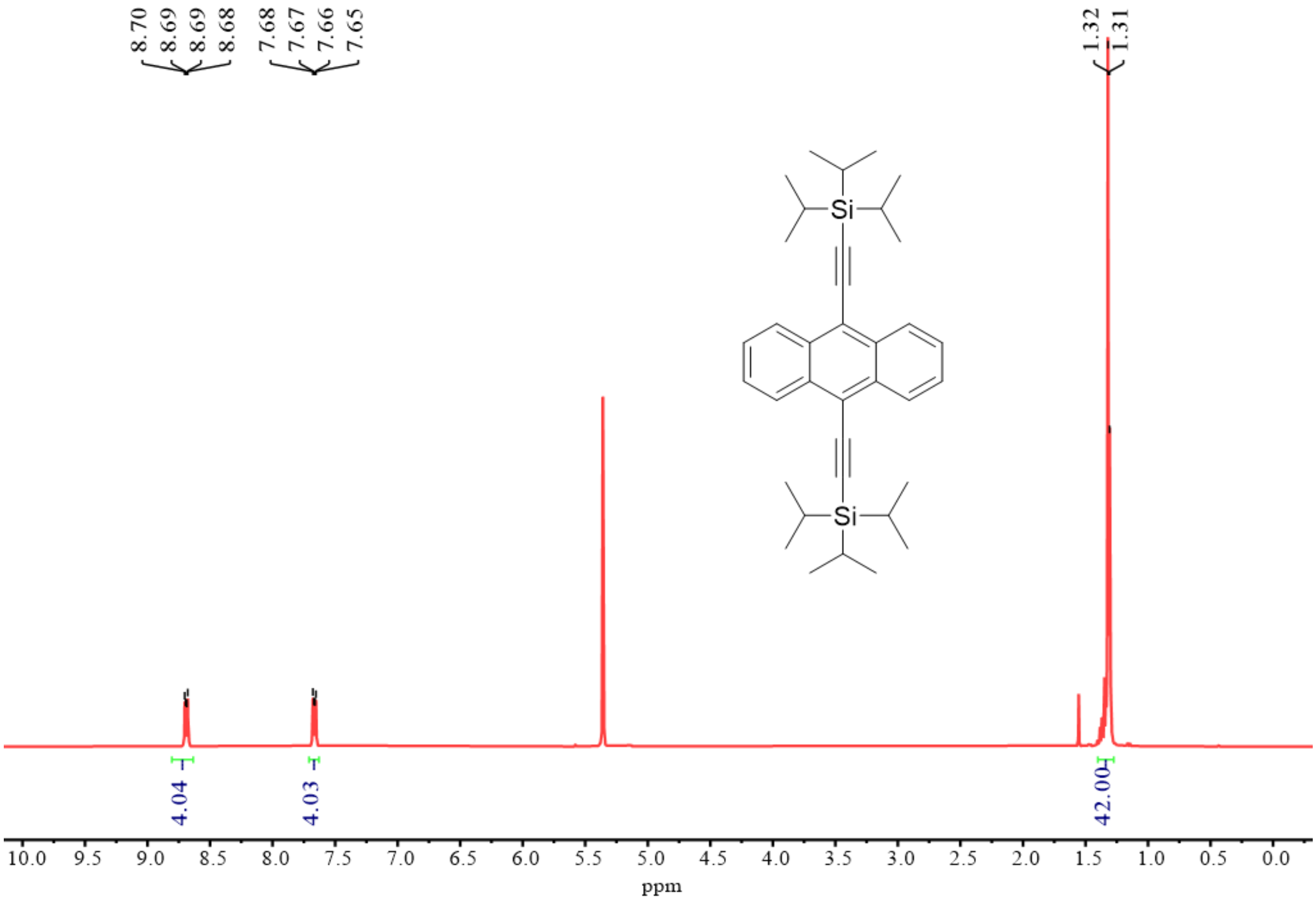

Figure S57: ^{1}H-NMR spectrum of AnTIPS in CD_2Cl_2.

12. References

(1) Coppens, P. The Evaluation of Absorption and Extinction in Single-Crystal Structure Analysis. In *Crystallographic Computing*, Ahmed, F. R. Ed.; Munksgaard, 1970; pp 255–270.
(2) Koziskova, J.; Hahn, F.; Richter, J.; Kožíšek, J. Comparison of different absorption corrections on the model structure of tetrakis(μ2-acetato)-diaqua-di-copper(II). *Acta Chim. Slov.* **2016**, *9*, 136–140, DOI: 10.1515/acs-2016-0023.
(3) Dolomanov, O. V.; Bourhis, L. J.; Gildea, R. J.; Howard, J. A. K.; Puschmann, H. OLEX2: a complete structure solution, refinement and analysis program. *J. Appl. Crystallogr.* **2009**, *42*, 339–341, DOI: 10.1107/S0021889808042726.
(4) Sheldrick, G. SHELXT - Integrated space-group and crystal-structure determination. *Acta Crystallogr. A* **2015**, *71*, 3–8, DOI: 10.1107/S2053273314026370.
(5) Kratzert, D.; Holstein, J. J.; Krossing, I. DSR: enhanced modelling and refinement of disordered structures with SHELXL. *J. Appl. Crystallogr.* **2015**, *48*, 933–938, DOI: 10.1107/S1600576715005580.
(6) Bill, E. julX. Max-Planck Institute for Chemical Energy Conversion: Mülheim/Ruhr, Germany, 2008.
(7) Suzuki, K.; Kobayashi, A.; Kaneko, S.; Takehira, K.; Yoshihara, T.; Ishida, H.; Shiina, Y.; Oishi, S.; Tobita, S. Reevaluation of absolute luminescence quantum yields of standard solutions using a spectrometer with an integrating sphere and a back-thinned CCD detector. *Phys. Chem. Chem. Phys.* **2009**, *11*, 9850–9860, DOI: 10.1039/B912178A.
(8) Frisch, M. J.; Trucks, G. W.; Schlegel, H. B.; Scuseria, G. E.; Robb, M. A.; Cheeseman, J. R.; Scalmani, G.; Barone, V.; Petersson, G. A.; Nakatsuji, H.; Li, X.; Caricato, M.; Marenich, A. V.; Bloino, J.; Janesko, B. G.; Gomperts, R.; Mennucci, B.; Hratchian, H. P.; Ortiz, J. V.; Izmaylov, A. F.; Sonnenberg, J. L.; Williams; Ding, F.; Lipparini, F.; Egidi, F.; Goings, J.; Peng, B.; Petrone, A.; Henderson, T.; Ranasinghe, D.; Zakrzewski, V. G.; Gao, J.; Rega, N.; Zheng, G.; Liang, W.; Hada, M.; Ehara, M.; Toyota, K.; Fukuda, R.; Hasegawa, J.; Ishida, M.; Nakajima, T.; Honda, Y.; Kitao, O.; Nakai, H.; Vreven, T.; Throssell, K.; Montgomery Jr., J. A.; Peralta, J. E.; Ogliaro, F.; Bearpark, M. J.; Heyd, J. J.; Brothers, E. N.; Kudin, K. N.; Staroverov, V. N.; Keith, T. A.; Kobayashi, R.; Normand, J.; Raghavachari, K.; Rendell, A. P.; Burant, J. C.; Iyengar, S. S.; Tomasi, J.; Cossi, M.; Millam, J. M.; Klene, M.; Adamo, C.; Cammi, R.; Ochterski, J. W.; Martin, R. L.; Morokuma, K.; Farkas, O.; Foresman, J. B.; Fox, D. J. Gaussian 16 Rev. C.01. **2016**.
(9) Vosko, S. H.; Wilk, L.; Nusair, M. Accurate spin-dependent electron liquid correlation energies for local spin density calculations: a critical analysis. *Can. J. Phys.* **1980**, *58*, 1200–1211, DOI: 10.1139/p80-159.
(10) Becke, A. D. Density-functional thermochemistry. III. The role of exact exchange. *J. Chem. Phys.* **1993**, *98*, 5648–5652, DOI: 10.1063/1.464913.

(11) Stephens, P. J.; Devlin, F. J.; Chabalowski, C. F.; Frisch, M. J. Ab Initio Calculation of Vibrational Absorption and Circular Dichroism Spectra Using Density Functional Force Fields. *J. Chem. Phys.* **1994**, *98*, 11623–11627, DOI: 10.1021/j100096a001.
(12) Lee, C.; Yang, W.; Parr, R. G. Development of the Colle-Salvetti correlation-energy formula into a functional of the electron density. *Phys. Rev. B* **1988**, *37*, 785, DOI: 10.1103/PhysRevB.37.785.
(13) Weigend, F.; Ahlrichs, R. Balanced basis sets of split valence, triple zeta valence and quadruple zeta valence quality for H to Rn: Design and assessment of accuracy. *Phys. Chem. Chem. Phys.* **2005**, *7*, 3297–3305, DOI: 10.1039/B508541A.
(14) Marenich, A. V.; Cramer, C. J.; Truhlar, D. G. Universal Solvation Model Based on Solute Electron Density and on a Continuum Model of the Solvent Defined by the Bulk Dielectric Constant and Atomic Surface Tensions. *J. Phys. Chem. B* **2009**, *113*, 6378–6396, DOI: 10.1021/jp810292n.
(15) O'Boyle, N. M.; Tenderholt, A. L.; Langner, K. M. A library for package-independent computational chemistry algorithms. *J. Comput. Chem.* **2008**, *29*, 839–845, DOI: 10.1002/jcc.20823.
(16) Hanwell, M. D.; Curtis, D. E.; Lonie, D. C.; Vandermeersch, T.; Zurek, E.; Hutchison, G. R. Avogadro: an advanced semantic chemical editor, visualization, and analysis platform. *J. Cheminform.* **2012**, *4*, 17, DOI: 10.1186/1758-2946-4-17.
(17) Tange, O. GNU Parallel - The Command-Line Power Tool. *The USENIX Magazine* **2011**, *36*, 42–47.
(18) Humphrey, W.; Dalke, A.; Schulten, K. VMD: visual molecular dynamics. *J. Mol. Graph.* **1996**, *14*, 33–38, 27–38, DOI: 10.1016/0263-7855(96)00018-5.
(19) Povray.org. (accessed October 2024).
(20) Müller, C.; Pascher, T.; Eriksson, A.; Chabera, P.; Uhlig, J. KiMoPack: A python Package for Kinetic Modeling of the Chemical Mechanism. *J. Phys. Chem. A* **2022**, *126*, 4087–4099, DOI: 10.1021/acs.jpca.2c00907.
(21) Abrahamsson, M.; Jäger, M.; Österman, T.; Eriksson, L.; Persson, P.; Becker, H.-C.; Johansson, O.; Hammarström, L. A 3.0 μs Room Temperature Excited State Lifetime of a Bistridentate RuII−Polypyridine Complex for Rod-like Molecular Arrays. *J. Am. Chem. Soc.* **2006**, *128*, 12616–12617, DOI: 10.1021/ja064262y.
(22) Hammecke, H.; Fritzler, D.; Vashistha, N.; Jin, P.; Dietzek-Ivanšić, B.; Wang, C. 100 μs Luminescence Lifetime Boosts the Excited State Reactivity of a Ruthenium(II)-Anthracene Complex in Photon Upconversion and Photocatalytic Polymerizations with Red Light. *Chem. Eur. J.* **2024**, *30*, e202402679, DOI: 10.1002/chem.202402679.
(23) Trippmacher, S.; Demeshko, S.; Prescimone, A.; Meyer, F.; Wenger, O. S.; Wang, C. Ferromagnetically Coupled Chromium(III) Dimer Shows Luminescence and Sensitizes Photon Upconversion. *Chem. Eur. J.* **2024**, *30*, e202400856, DOI: 10.1002/chem.202400856.
(24) Sirven, A. M.; Garbage, R.; Qiao , Y.; Kammerer , C.; Rapenne , G. Synthesis of Functionalized Mono-, Bis-, and Trisethynyltriptycenes for One-Dimensional Self-Assembly on Surfaces. *Chem. Eur. J.* **2015**, *21*, 15013–15019, DOI: 10.1002/chem.201502195.
(25) Wang, C.; Wegeberg, C.; Wenger, O. S. First-Row d6 Metal Complex Enables Photon Upconversion and Initiates Blue Light-Dependent Polymerization with Red Light. *Angew. Chem. Int. Ed.* **2023**, *62*, e202311470, DOI: 10.1002/anie.202311470.
(26) Jin, P.; Xu, X.; Hübner, D.; Yan, Y.; Wang, C. Beyond the Diffusion Limit: Preassociation Enhanced Photon Upconversion and Photocatalysis Sensitized by Iron Complex. *ACS Catal.* **2026**, *16*, 4040–4052, DOI: 10.1021/acscatal.6c00039.
(27) Spek, A. L. PLATON SQUEEZE: a tool for the calculation of the disordered solvent contribution to the calculated structure factors. *Acta Crystallogr. C* **2015**, *71*, 9–18, DOI: 10.1107/S2053229614024929.
(28) Duncan Lyngdoh, R. H.; Schaefer, H. F., III; King, R. B. Metal–Metal (MM) Bond Distances and Bond Orders in Binuclear Metal Complexes of the First Row Transition Metals Titanium Through Zinc. *Chem. Rev.* **2018**, *118*, 11626–11706, DOI: 10.1021/acs.chemrev.8b00297.
(29) Fraser, H. W. L.; Smythe, L.; Dey, S.; Nichol, G. S.; Piligkos, S.; Rajaraman, G.; Brechin, E. K. A simple methodology for constructing ferromagnetically coupled Cr(III) compounds. *Dalton Trans.* **2018**, *47*, 8100–8109, DOI: 10.1039/C8DT01963K.
(30) Fritzler, D.; Wang, C. Luminescent Dinuclear Chromium(III) Complexes with Ferromagnetic Exchange Interactions. *ChemPhotoChem* **2025**, *9*, e202500094, DOI: 10.1002/cptc.202500094.
(31) Janiak, C. A critical account on π–π stacking in metal complexes with aromatic nitrogen-containing ligands. *J. Chem. Soc., Dalton Trans.* **2000**, 3885–3896, DOI: 10.1039/B003010O.
(32) Börner, M.; Klose, J.; Gutierrez Suburu, M. E.; Strassert, C. A.; Yang, F.; Monakhov, K. Y.; Abel, B.; Kersting, B. Synthesis and Characterisation of Luminescent $[Cr^{III}{}_2L(\mu\text{-carboxylato})]^{3+}$ Complexes with High-Spin S=3 Ground States (L=N_6S_2 donor ligand). *Chem. Eur. J.* **2021**, *27*, 14899–14910, DOI: 10.1002/chem.202102079.
(33) Treiling, S.; Wang, C.; Förster, C.; Reichenauer, F.; Kalmbach, J.; Boden, P.; Harris, J. P.; Carrella, L. M.; Rentschler, E.; Resch-Genger, U.; Reber, C.; Seitz, M.; Gerhards, M.; Heinze, K. Luminescence and Light-Driven Energy and Electron Transfer from an Exceptionally Long-Lived Excited State of a Non-Innocent Chromium(III) Complex. *Angew. Chem. Int. Ed.* **2019**, *58*, 18075–18085, DOI: 10.1002/anie.201909325.
(34) Abdel-Shafi, A. A.; Beer, P. D.; Mortimer, R. J.; Wilkinson, F. Photosensitized Generation of Singlet Oxygen from (Substituted Bipyridine)ruthenium(II) Complexes. *Helv. Chim. Acta* **2001**, *84*, 2784–2795, DOI: 10.1002/1522-2675(20010919)84:9<2784::AID-HLCA2784>3.0.CO;2-U.
(35) Franco, C.; Olmsted, J. Photochemical determination of the solubility of oxygen in various media. *Talanta* **1990**, *37*, 905–909, DOI: 10.1016/0039-9140(90)80251-A.

(36) Achord, J. M.; Hussey, C. L. Determination of dissolved oxygen in nonaqueous electrochemical solvents. *Anal. Chem.* **2002**, *52*, 601–602, DOI: 10.1021/ac50053a061.
(37) Jiménez, J.-R.; Doistau, B.; Cruz, C. M.; Besnard, C.; Cuerva, J. M.; Campaña, A. G.; Piguet, C. Chiral Molecular Ruby $[Cr(dqp)_2]^{3+}$ with Long-Lived Circularly Polarized Luminescence. *J. Am. Chem. Soc.* **2019**, *141*, 13244–13252, DOI: 10.1021/jacs.9b06524.
(38) Otto, S.; Grabolle, M.; Förster, C.; Kreitner, C.; Resch-Genger, U.; Heinze, K. $[Cr(ddpd)_2]^{3+}$: A Molecular, Water-Soluble, Highly NIR-Emissive Ruby Analogue. *Angew. Chem. Int. Ed.* **2015**, *54*, 11572–11576, DOI: 10.1002/anie.201504894.
(39) Reichenauer, F.; Wang, C.; Förster, C.; Boden, P.; Ugur, N.; Báez-Cruz, R.; Kalmbach, J.; Carrella, L. M.; Rentschler, E.; Ramanan, C.; Niedner-Schatteburg, G.; Gerhards, M.; Seitz, M.; Resch-Genger, U.; Heinze, K. Strongly Red-Emissive Molecular Ruby $[Cr(bpmp)_2]^{3+}$ Surpasses $[Ru(bpy)_3]^{2+}$. *J. Am. Chem. Soc.* **2021**, *143*, 11843–11855, DOI: 10.1021/jacs.1c05971.
(40) Partanen, I.; Hsu, C.-H.; Shi, E. H.-C.; Maisuls, I.; Eskelinen, T.; Karttunen, A. J.; Saarinen, J. J.; Strassert, C. A.; Belyaev, A.; Chou, P.-T.; Koshevoy, I. O. Organic Room-Temperature near-IR Phosphorescence Harvested by Intramolecular Through-Space Sensitization in Composite Molecules. *Angew. Chem. Int. Ed.* **2025**, *64*, e202503327, DOI: 10.1002/anie.202503327.
(41) Reineke, S.; Baldo, M. A. Room temperature triplet state spectroscopy of organic semiconductors. *Sci. Rep.* **2014**, *4*, 3797, DOI: 10.1038/srep03797.
(42) Montalti, M.; Credi, A.; Prodi, L.; Gandolfi, M. T. *Handbook of Photochemistry*; Taylor and Francis, 2006.
(43) Pfeil, A. Quenching of the phosphorescence of some chromium(III) complexes by molecular oxygen. *J. Am. Chem. Soc.* **1971**, *93*, 5395–5398, DOI: 10.1021/ja00750a013.
(44) Doettinger, F.; Sagaya, J.; Morselli, G.; Wenger, O. S. Homomolecular Photon Upconversion in a Perylene-Decorated Iron(III) Complex. *J. Am. Chem. Soc.* **2025**, *147*, 43013–43028, DOI: 10.1021/jacs.5c16091.
(45) Doettinger, F.; Wenger, O. S. Unimolecular Doublet-Triplet Annihilation Upconversion in Iron(III) Coordination Compounds. *J. Am. Chem. Soc.* **2026**, *148*, 19399–19411, DOI: 10.1021/jacs.6c05446.
(46) Shen, Y.; Liu, H.; Cao, J.; Zhang, S.; Li, W.; Yang, B. Unusual temperature-sensitive excimer fluorescence from discrete π-π dimer stacking of anthracene in a crystal. *Phys. Chem. Chem. Phys.* **2019**, *21*, 14511–14515, DOI: 10.1039/c9cp02656h.
(47) Otto, S.; Nauth, A. M.; Ermilov, E.; Scholz, N.; Friedrich, A.; Resch-Genger, U.; Lochbrunner, S.; Opatz, T.; Heinze, K. Photo-Chromium: Sensitizer for Visible-Light-Induced Oxidative C−H Bond Functionalization—Electron or Energy Transfer? *ChemPhotoChem* **2017**, *1*, 344–349, DOI: 10.1002/cptc.201700077.
(48) Glaser, F.; Beneventi, G. M.; Cadranel, A.; Troian-Gautier, L. Beyond Common Energy Transfer: Intramolecular Electron Transfer Cascade Controls Triplet Population of a Long-Lived Iron-Anthracene Molecular Dyad. *ACS Cent. Sci.* **2025**, *11*, 1870–1881, DOI: 10.1021/acscentsci.5c01040.
(49) Jin, T.; Lazarevski, B.; Wellauer, J.; Morselli, G.; Wenger, O. S. Energy Storage in the Outer Coordination Sphere of a Chromium(0) Complex Enhances Photophysical and Photochemical Properties. *J. Am. Chem. Soc.* **2026**, *148*, 15971–15982, DOI: 10.1021/jacs.5c23284.
(50) Gray, V.; Dzebo, D.; Lundin, A.; Alborzpour, J.; Abrahamsson, M.; Albinsson, B.; Moth-Poulsen, K. Photophysical characterization of the 9,10-disubstituted anthracene chromophore and its applications in triplet–triplet annihilation photon upconversion. *J. Mater. Chem. C* **2015**, *3*, 11111–11121, DOI: 10.1039/C5TC02626A.
(51) Parker, C. A.; Hatchard, C. G. Delayed fluorescence from solutions of anthracene and phenanthrene. *Proc. R. Soc. Lond. A* **1962**, *269*, 574–584, DOI: 10.1098/rspa.1962.0197.
(52) Edhborg, F.; Olesund, A.; Albinsson, B. Best practice in determining key photophysical parameters in triplet–triplet annihilation photon upconversion. *Photochem. Photobiol. Sci.* **2022**, *21*, 1143–1158, DOI: 10.1007/s43630-022-00219-x.
(53) Bock, C. R.; Connor, J. A.; Gutierrez, A. R.; Meyer, T. J.; Whitten, D. G.; Sullivan, B. P.; Nagle, J. K. Estimation of excited-state redox potentials by electron-transfer quenching. Application of electron-transfer theory to excited-state redox processes. *J. Am. Chem. Soc.* **2002**, *101*, 4815–4824, DOI: 10.1021/ja00511a007.
(54) Salah, N. B.; Matoussi, F. Electrochemical study of the anodic oxidation of terpinenes at the electrode and by homogeneous redox catalysis. *J. Electroanal. Chem.* **2005**, *583*, 1–7, DOI: 10.1016/j.jelechem.2004.08.025.
(55) Tiruye, H. M.; Economopoulos, S.; Jørgensen, K. B. Synthesis of polycyclic aromatic quinones by continuous flow electrochemical oxidation: anodic methoxylation of polycyclic aromatic phenols (PAPs). *Beilstein J. Org. Chem.* **2024**, *20*, 1746–1757, DOI: 10.3762/bjoc.20.153.
(56) Ashnagar, A.; Bruce, J. M.; Dutton, P. L.; Prince, R. C. One- and two-electron reduction of hydroxy-1,4-naphthoquinones and hydroxy-9,10-anthraquinones: The role of internal hydrogen bonding and its bearing on the redox chemistry of the anthracycline antitumour quinones. *Biochim. Biophys. Acta* **1984**, *801*, 351–359, DOI: 10.1016/0304-4165(84)90138-7.
